# Supplementary material for: Experiences and well-being of healthcare professionals working in the field of ultrasound in obstetrics and gynaecology as the SARS-CoV-2 pandemic were evolving: a cross-sectional survey study
Source: BMJ Open. 2022 Feb 4;12(2):e051700. doi: 10.1136/bmjopen-2021-051700 (PMC8819548; doi:10.1136/bmjopen-2021-051700)
Supplement: Supplementary data [file bmjopen-2021-051700supp001.pdf]

SUPPLEMENTARY MATERIAL

The experiences and wellbeing of healthcare professionals working in the field of ultrasound in Obstetrics and Gynecology as the SARS-CoV-2 pandemic was evolving: a cross-sectional survey study

Authors

Tom Bourne[1,2], Christopher Kyriacou[1], Harsha Shah[1], Jolien Ceusters[3], Jessica Preisler[4,5], Ulrike Metzger[6], Chiara Landolfo[7], Christoph Lees[8], Dirk Timmerman[2,9]

Affiliations

1. Early pregnancy and Acute Gynaecology Unit, Department of Obstetrics and Gynaecology, Queen Charlotte's and Chelsea Hospital, Imperial College, London, UK.
2. Department of Development and Regeneration, KU Leuven, Leuven, Belgium.
3. Department of Oncology, Laboratory of Tumour Immunology and Immunotherapy, KU Leuven, Leuven, Belgium.
4. Facultad de Medicina, Clínica Alemana – Universidad del Desarrollo, Santiago, Chile
5. Hospital Clínico de la Universidad de Chile, Santiago, Chile.
6. Centre d'Échographie de l'Odéon, Paris, France.
7. Dipartimento Scienze della Salute della Donna, del Bambino e di Sanità Pubblica, Fondazione Policlinico Universitario Agostino Gemelli, IRCCS, Rome, Italy.
8. Centre for Fetal Care, Department of Obstetrics and Gynaecology, Queen Charlotte's and Chelsea Hospital, Imperial College, London, UK.
9. Department of Obstetrics and Gynaecology, University Hospitals Leuven, Leuven, Belgium.

Correspondence to:
Professor Tom Bourne
Queen Charlotte's and Chelsea Hospital, Imperial College London
Du Cane Road, London, W12 0HS, UK
t.bourne@imperial.ac.uk

BMJ Publishing Group Limited (BMJ) disclaims all liability and responsibility arising from any reliance placed on this supplemental material which has been supplied by the author(s)

Supplement 1: The International Society of Ultrasound in Obstetrics and Gynecology (ISUOG) SARS-CoV-2 Questionnaire for healthcare professionals working with ultrasound in Obstetrics and Gynecology.

Informed consent

This questionnaire is fully anonymous - answers cannot be traced back to participants. The anonymised data will be collected for analysis in order to provide a report and publication at a later date.

1. **Please click 'yes' to participate in this survey. Otherwise, click 'no': (please choose one option)**

Yes
No

Introduction

2. **What title best describes your job: (please choose one option)**

Sonographer – Obstetrics
Sonographer – Gynecology
Sonographer – Obstetrics and Gynecology
Obstetrician
Gynecologist
Obstetrician and Gynecologist
Radiologist
Doctor in training – Obstetrics
Doctor in training – Gynecology
Doctor in training – Obstetrics and Gynecology
Doctor in training – Radiologist
Doctor in research – Obstetrics
Doctor in research – Gynecology
Doctor in research – Obstetrics and Gynecology
Doctor in research – Radiology
Nurse – Obstetrics
Nurse – Gynecology
Nurse – Obstetrics and Gynecology
Nurse – Early pregnancy only
Midwife – Obstetrics
Midwife – Early pregnancy only
Retired

3. **In what country do you practice? (please state below)**

Afghanistan
Albania
Algeria
Andorra
Angola

1 Antigua and Barbuda
2 Argentina
3 Armenia
4 Australia
5 Austria
6 Azerbaijan
7 The Bahamas
8 Bahrain
9 Bangladesh
10 Barbados
11 Belarus
12 Belgium
13 Belize
14 Benin
15 Bhutan
16 Bolivia
17 Bosnia and Herzegovina
18 Botswana
19 Brazil
20 Brunei
21 Bulgaria
22 Burkina Faso
23 Burundi
24 Cabo Verde
25 Cambodia
26 Cameroon
27 Canada
28 Central African Republic
29 Chad
30 Chile
31 China
32 Colombia
33 Comoros
34 Congo, Democratic Republic of the
35 Congo, Republic of the
36 Costa Rica
37 Côte d’Ivoire
38 Croatia
39 Cuba
40 Cyprus
41 Czech Republic
42 Denmark
43 Djibouti
44 Dominica
45 Dominican Republic
46 East Timor (Timor-Leste)
47 Ecuador

Egypt
El Salvador
Equatorial Guinea
Eritrea
Estonia
Eswatini
Ethiopia
Fiji
Finland
France
Gabon
The Gambia
Georgia
Germany
Ghana
Greece
Grenada
Guatemala
Guinea
Guinea-Bissau
Guyana
Haiti
Honduras
Hungary
Iceland
India
Indonesia
Iran
Iraq
Ireland
Israel
Italy
Jamaica
Japan
Jordan
Kazakhstan
Kenya
Kiribati
Korea, North
Korea, South
Kosovo
Kuwait
Kyrgyzstan
Laos
Latvia
Lebanon
Lesotho

Liberia
Libya
Liechtenstein
Lithuania
Luxembourg
Madagascar
Malawi
Malaysia
Maldives
Mali
Malta
Marshall Islands
Mauritania
Mauritius
Mexico
Micronesia, Federated States of
Moldova
Monaco
Mongolia
Montenegro
Morocco
Mozambique
Myanmar (Burma)
Namibia
Nauru
Nepal
Netherlands
New Zealand
Nicaragua
Niger
Nigeria
North Macedonia
Norway
Oman
Pakistan
Palau
Panama
Papua New Guinea
Paraguay
Peru
Philippines
Poland
Portugal
Qatar
Romania
Russia
Rwanda

Saint Kitts and Nevis
Saint Lucia
Saint Vincent and the Grenadines
Samoa
San Marino
Sao Tome and Principe
Saudi Arabia
Senegal
Serbia
Seychelles
Sierra Leone
Singapore
Slovakia
Slovenia
Solomon Islands
Somalia
South Africa
Spain
Sri Lanka
Sudan
Sudan, South
Suriname
Sweden
Switzerland
Syria
Taiwan
Tajikistan
Tanzania
Thailand
Togo
Tonga
Trinidad and Tobago
Tunisia
Turkey
Turkmenistan
Tuvalu
Uganda
Ukraine
United Arab Emirates
United Kingdom
United States
Uruguay
Uzbekistan
Vanuatu
Vatican City
Venezuela
Vietnam

Yemen
Zambia
Zimbabwe

4. **Are you an ISUOG member? (please choose one option)**

Yes
No

5. **If you answered no, which society or professional body did you receive this survey from?**

Open box answer

Demographics

6. **How old are you? (please state number of years)**

Numbers offered

7. **What sex were you assigned at birth? (please choose one option)**

Male
Female
Intersex
Prefer not to say

8. **What gender do you most identify with? (please choose one option)**

Male
Female
Prefer not to say
Other (please specify)

Underlying medical conditions

9. **Do you have any underlying medical conditions that place you at increased risk during the SARS-CoV-2 pandemic? (please tick all that apply)**

None
Heart disease
Hypertension
Diabetes
Asthma
Other lung disease
Kidney disease
Cancer

Medication

10. Are you taking any of the following medications? (please tick all that apply)

None
Immunosuppressant medication - steroids
Immunosuppressant medication - methotrexate
Immunosuppressant medication - biological agents
Aspirin
Indomethacin
Any other non-steroidal anti-inflammatory drugs (NSAIDS) e.g. ibuprofen, diclofenac, naproxen
Angiotensin converting enzyme inhibitors (ACEi) blood pressure medication - any ending in -pril (e.g. enalapril)
Any other blood pressure medication e.g. angiotensin receptor blockers (ARB), alpha blockers, alpha-2 receptor agonists, beta blockers, combined alpha and beta blockers, calcium channel blockers (CCB), diuretics, other

Smoking and BMI

11. Do you smoke? (please choose one option)

Yes
Previously – now stopped
No

12. What was your last measured weight? (please choose one option)

In kilograms (kg) – numbers offered

13. What is your height? (please choose one option)

In centimetres (cm) – numbers offered

Role, shift pattern, PPE

14. How has your role and shift pattern been affected by the SARS-CoV-2 pandemic? (please choose one option)

Unable to work for the duration of the pandemic
Reduced duties with no patient contact
Reduced duties with reduced patient contact
Reduced duties with similar patient contact
Reduced duties with increased patient contact
Similar duties with no patient contact
Similar duties with reduced patient contact
Similar duties with similar patient contact
Similar duties with increased patient contact
Increased duties with no patient contact
Increased duties with reduced patient contact

Increased duties with similar patient contact
Increased duties with increased patient contact

15. Where are you working during the SARS-CoV-2 pandemic? (please tick all that apply)

Obstetrics – Labor ward
Obstetrics – Birth center
Obstetrics – Antenatal ward
Obstetrics – Postnatal ward
Obstetrics – Maternity triage
Obstetrics – Antenatal clinic
Obstetrics – Maternity helplines
Obstetrics – Community clinics
Obstetrics – Ultrasound
Gynecology – Acute gynecology
Gynecology – Benign gynecology
Gynecology – Oncology
Gynecology – Ultrasound
Early pregnancy unit – Ultrasound and assessment
Radiology – Ultrasound
Radiology – non-ultrasound
Obstetrics/Gynecology/Radiology – From home

16. Do you interact with patients confirmed or suspected to have SARS-CoV-2? (please choose one option)

Yes – every day
Yes – every week
Yes – every so often
I don't know
Not yet
No

17. Have you been using personal protective equipment (PPE) since the SARS-CoV-2 outbreak? (please choose one option)

No
Not yet
Yes – for aerosol generating procedures only
Yes – for suspected/positive patients only
Yes – for all patients

18. When you perform an ultrasound scan please state what PPE you use: (please tick all that apply)

None
Gloves
Surgical mask
N95/FFP3 mask
Gown

Visor
Surgical hat

19. **Have there been any concerns regarding PPE provision or shortages since the SARS-CoV-2 outbreak? (note – Full PPE includes a N95/FFP3 mask, visor, surgical hat, gown and gloves) (please tick all that apply)**

No issues with provision
Yes – supply issues with gloves
Yes – supply issues with surgical masks
Yes – supply issues with N95/FFP3 masks
Yes – supply issues with gowns
Yes – supply issues with visors
Yes – supply issues with surgical hats
Yes – supply issues with providing patients with a surgical mask

20. **Have you been asked to re-use or recycle any PPE? (please tick all that apply option)**

No
Yes - Gloves
Yes - Surgical mask
Yes - N95/FFP3 mask
Yes - Gown
Yes - Visor
Yes - Surgical hat

21. **Do you ask patients to wear a surgical mask? (please choose one option)**

Yes
No

SARS-CoV-2 diagnosis

22. **Have you had or do you have SARS-CoV-2 based on clinical symptoms and/or a SARS-CoV-2 swab (PCR) diagnosis? (please choose one option)**

No – no test or symptoms suggestive of SARS-CoV-2
Yes – suspected based on symptoms but have not had PCR swab testing
Yes – suspected based on symptoms, had PCR testing, which was negative
Yes – confirmed on PCR swab testing but have had no symptoms
Yes – suspected based on symptoms and confirmed on PCR swab testing

23. **With regards to having a SARS-CoV-2 swab test, please choose one option:**

Not been offered
Offered as experiencing symptoms
Offered as in contact with confirmed or probable SARS-CoV-2 patients
Offered as living with someone who has probable or confirmed SARS-CoV-2
Not applicable

24. **What symptoms have you experienced or are you experiencing? (please tick all that apply)**

None
High fever
Persistent dry cough
Productive cough
Shortness of breath/Difficulty breathing
Fatigue
Headache
Loss of sense of smell and/or taste
Sore throat
Chest pain
Diarrhoea
Myalgia
Loss of appetite
Hoarse voice
Abdominal pain

Personal SARS-CoV-2 pathway

25. **At what point were you offered a SARS-CoV-2 swab? (please state number of days into your illness with day 1 being the day you started to have symptoms. If you have not had symptoms – please choose 'not had symptoms'. If you were not offered a test, please choose 'not offered a test')**

Not had symptoms
Not offered a test
Not applicable
Numbers offered

26. **How severe was/is your infection? (please choose one option)**

Mild COVID-19 (T< 37.8 oC, tiredness, muscle aches, headache, no breathlessness, normal appetite)
Moderate COVID-19 (T> 37.8 oC, troublesome cough, breathless on exercise, tired but no chest or abdominal pain)
Severe COVID-19 (T> 37.8 oC , noticeably breathless even when sitting still, unable to complete sentences when talking, chest or abdominal pain, reduced appetite)
Critical COVID-19 (development of severe acute respiratory syndrome in need of ventilator or other respiratory support)
Not applicable

27. **How long were you or have you been symptomatic for? (please state number of days. If you have not had symptoms – please choose 'not had symptoms')**

Not had symptoms
Numbers offered

28. How long did you stay at home before going back to work, or if current infection, how long have you been at home for? (please state number of days. If you have not had symptoms – please choose 'not had symptoms')

Not had symptoms
Numbers offered

29. Did you or do you feel under pressure from your employers to return to work before making a full recovery? (please choose one option)

Yes
No
Not applicable

30. If you have been unwell with presumed or confirmed SARS-CoV-2, how long were you advised to stay at home by your employers before going back to work? (please state number of days. If you have not had symptoms – please choose 'not had symptoms')

Not had symptoms
Numbers offered

31. Have you had a SARS-CoV-2 antibody test? (please choose one option)

No – no test or symptoms suggestive of SARS-CoV-2
No – not offered
No – offered and declined
Yes – negative
Yes – positive

<u>Household</u>

32. Have you needed to, or are you currently self-isolating due to a member of your household having suspected or confirmed SARS-CoV-2? (please choose one option)

Yes
No

33. How long did you need or have you needed to self-isolate due to a member of your household having suspected or confirmed SARS-CoV-2? (please state number of days. If your household has been well - please choose 'household has been well')

Household has been well
Numbers offered

Colleagues

34. **How many colleagues do you work with in your unit? (please state number of each group. Select 'unsure' if you do not know)**

Sonographers
Consultants
Doctors in training
Doctors in research
Nurses
Midwifes
Unsure
Numbers offered

35. **To your knowledge, how many colleagues are you aware of in your unit that have had a presumed diagnosis of SARS-CoV-2 based on symptoms or a positive swab? (please state number of each group. Select 'unsure' if you have no information)**

Sonographers
Consultants
Doctors in training
Doctors in research
Nurses
Midwifes
Unsure
Numbers offered

36. **To your knowledge, how many colleagues have needed to self-isolate or are self-isolating due to a member of their household having suspected or confirmed SARS-CoV-2? (please state number of each group. Select 'unsure' if you have no information)**

Sonographers
Consultants
Doctors in training
Doctors in research
Nurses
Midwifes
Unsure
Numbers offered

37. **What is the longest period of time any of your colleagues have taken off in isolation because they or a household member have suspected or confirmed SARS-CoV-2? (please state number of days. Select 'unsure' if you have no information)**

Unsure
Numbers offered

Support

38. **Have you felt or do you feel adequately supported by your unit during the SARS-CoV-2 pandemic? (please choose one option)**

Yes
No

39. **How do you rate the response and support that was provided by your country's Ministry of Health or Public Health officials? (please answer using the scale below)**

0 - Extremely poor
50 - Adequate
100 - Excellent

40. **Please tell us, if you wish, your views regarding how well clinician safety has been protected by your employers (please state in the text box below. If you choose not to answer, please write 'not applicable')**

Open box answer

Hospital Anxiety and Depression Scale (HADS)

41. **Thank you for filling out this questionnaire.**

Optional: Hospital Anxiety and Depression Scale (HADS). If you are willing to proceed, please click yes. Otherwise, click no to end the survey.

Yes
No

Hospital Anxiety and Depression Scale (HADS)

42. **Choose the reply that is closest to how you have been feeling in the past week. Don't take too long over your replies: your immediate is best.**

I feel tense or 'wound up':

Most of the time
A lot of the time
From time to time, occasionally
Not at all

43. **Choose the reply that is closest to how you have been feeling in the past week. Don't take too long over your replies: your immediate is best.**

I still enjoy the things I used to enjoy:

Definitely as much
Not quite as much

Only a little
Hardly at all

44. Choose the reply that is closest to how you have been feeling in the past week. Don't take too long over your replies: your immediate is best.

I get a sort of frightened feeling as if something awful is about to happen:
Very definitely and quite badly
Yes, but not too badly
A little, but it doesn't worry me
Not at all

45. Choose the reply that is closest to how you have been feeling in the past week. Don't take too long over your replies: your immediate is best.

I can laugh and see the funny side of things:
As much as I always could
Not quite so much now
Definitely not so much now
Not at all

46. Choose the reply that is closest to how you have been feeling in the past week. Don't take too long over your replies: your immediate is best.

Worrying thoughts go through my mind:
A great deal of the time
A lot of the time
From time to time, but not too often
Only occasionally

47. Choose the reply that is closest to how you have been feeling in the past week. Don't take too long over your replies: your immediate is best.

I feel cheerful:
Not at all
Not often
Sometimes
Most of the time

48. Choose the reply that is closest to how you have been feeling in the past week. Don't take too long over your replies: your immediate is best.

I can sit at ease and feel relaxed:
Definitely
Usually
Not often
Not at all

49. Choose the reply that is closest to how you have been feeling in the past week. Don’t take too long over your replies: your immediate is best.

I feel as if I am slowed down:

Nearly all the time
Very often
Sometimes
Not at all

50. Choose the reply that is closest to how you have been feeling in the past week. Don’t take too long over your replies: your immediate is best.

I get a sort of frightened feeling like 'butterflies' in the stomach:

Not at all
Occasionally
Quite often
Very often

51. Choose the reply that is closest to how you have been feeling in the past week. Don’t take too long over your replies: your immediate is best.

I have lost interest in my appearance:

Definitely
I don't take as much care as I should
I may not take quite as much care
I take just as much care as ever

52. Choose the reply that is closest to how you have been feeling in the past week. Don’t take too long over your replies: your immediate is best.

I feel restless as I have to be on the move:

Very much indeed
Quite a lot
Not very much
Not at all

53. Choose the reply that is closest to how you have been feeling in the past week. Don’t take too long over your replies: your immediate is best.

I look forward with enjoyment to things:

As much as I ever did
Rather less than I used to
Definitely less than I used to
Hardly at all

54. Choose the reply that is closest to how you have been feeling in the past week. Don’t take too long over your replies: your immediate is best.

I get sudden feelings of panic:
Very often indeed
Quite often
Not very often
Not at all

55. Choose the reply that is closest to how you have been feeling in the past week. Don’t take too long over your replies: your immediate is best.

I can enjoy a good book or radio or TV program:
Often
Sometimes
Not often
Very seldom

56. Thank you for filling out this questionnaire. Please complete last question to end. Please tell us, if you wish, how you have felt emotionally during the SARS-CoV-2 pandemic? (please state in the text box below. If you choose not to answer, please write 'not applicable')
Open box answer

Supplement 2: Original study protocol

Title: An international survey of the impact of COVID19 on healthcare professionals carrying out ultrasonography, service provision and mental state in Obstetrics and Gynecology

Date: 19th April 2020

Main Sponsor: UZ Leuven Belgium
Carried out in collaboration with: International Society of Ultrasound in Obstetrics and Gynecology (ISUOG)
Version number: 1
Principal Investigators**:** Professor Tom Bourne and Professor Dirk Timmerman
Co-Investigators: Dr Christopher Kyriacou, Dr Harsha Shah
Protocol to be assessed by: EC Onderzoek UZ Leuven, Belgium.

Design: Prospective cohort observational survey study.

Aim: To assess the services and psychological impact of the COVID-19 pandemic to healthcare professionals carrying out ultrasonography within the Obstetrics and Gynecology specialty.

Hypothesis:

1. Service provision to support healthcare professionals during the COVID-19 pandemic varies considerably in every unit globally;
2. Healthcare professionals will have experienced COVID-19 first-hand;
3. The COVID-19 pandemic has caused significant psychological distress to healthcare professionals which can be assessed using a validated psychological tool.

Outcome measures:

1. A questionnaire to healthcare professionals detailing medical history, role, area of work, personal protective equipment (PPE), COVID-19 patient contact and personal COVID-19 experience will allow data to be collected in relation to the physical impact of the COVID-19 pandemic;
2. Use of the validated Hospital Anxiety and Depression Scale (HADS) will allow psychological morbidity of healthcare professionals to be assessed.

Eligibility: Members of ISUOG that fill in the questions via an online survey platform. The questionnaire will in English, Spanish, Greek, Hindu, Mandarin and Cantonese.

Duration: The survey will be conducted over a period of two weeks in order to allow time for the questions to be answered, two reminder emails will be sent – one at a week before closure and one 24 hours before closure

Consent: Consent will be implied when the questionnaire is filled in and there will be a specific question asking for consent to take part in the survey, however all information is anonymized and cannot be traced to an individual. No data will be requested that is in breach of General Data Protection Regulation (GDPR).

Background: The current coronavirus (SARS-CoV-2) pandemic carries a number of new challenges to local, national and international healthcare agencies in order to protect patients and healthcare professionals (physicians, sonographers, allied healthcare professionals). Guidance varies from nation to nation with issues arising from redeployment of clinicians, prioritization of particular services and provision of PPE. This is important as those carrying out ultrasound scans are in close proximity to patients and are at high risk of SARS-CoV-2 infection[1].

Anxiety and depression are recognized complications for healthcare professionals working in a high-stress environment, particularly one that now encompasses a novel disease for which we have yet to fully understand[2].

Methods: This is a prospective cohort study and will recruit via the dissemination of a survey online through the ISUOG mailing list. Consent will be presumed when the form is filled in and submitted. This will allow healthcare professionals globally to answer. The questionnaire has been devised to assess the service provision in units around the world as well as question personal COVID-19 experience. Following on from this will be an optional HADS. The HADS is a 14-part questionnaire with 7 questions exploring symptoms of anxiety and depression, respectively. Each question is answered with a scale of 0-3 to represent severity of symptoms, with an independent maximum score of 21 for depression and anxiety. A score $\geq$11 is deemed moderate, whilst $\geq$16 represents severe symptoms[3].

Statistics and data analysis: Sample size will be dependent on the number of online responders. We hope that there will be significant uptake from a number of countries globally. The recruitment will be online so no breach to social distancing rules will apply. To compare differences in binary variables we will use the likelihood ratio, chi-squared or Fisher's exact test and will consider a p value <0.05 as statistically significant. Ninety-five per cent confidence intervals for rates of psychological morbidity will be created using the Wilson method.

Dissemination: Following the collection and analysis of the data obtained from the questionnaire, we aim to present the findings to the ISUOG membership and publish in peer-reviewed journals – preferably Ultrasound in Obstetrics and Gynecology. The need to understand the experience of the pandemic by frontline workers is extremely important, not only to support them now, but to prepare for any future international healthcare emergency.

References

1. Abramowicz JS, Basseal JM, Brezinka C, Dall'Asta A, Deng J, Harrison G, Marsal K, Lee JCS, Lim A, Miloro P, Poon LC, Salvesen KJ, Sande R, ter Haar G, Westerway SC, Xie MX, Lees C. ISUOG Safety Committee Position Statement on use of personal protective equipment and hazard mitigation in relation to SARS-CoV-2 for practitioners undertaking obstetric and gynecological ultrasound. *ISUOG*. 2020 March 28. Available from: https://www.isuog.org/uploads/assets/b8dde768-08a2-424c-a4615551637515e9/ISUOG-Safety-Committee-statement-on-COVID19-and-PPE.pdf
2. Neto MLR, Almeida HG, Esmeraldo JD, Nobre CB, Pinheiro WR, de Oliveira CRT, Sousa IDC, Lima OMML, Lima NNR, Moreira MM, Lima CKT, Junior JG, da Silva CGL.

When health professionals look death in the eye: the mental health of professionals who deal daily with the 2019 coronavirus outbreak. *Psychiatry Res*. 2020 April 13;288:112972. doi:10.1016/j.psychres.2020.112972.

3. Zigmond AS, Snaith RP. The hospital anxiety and depression scale. Acta psychiatrica scandinavica. 1983 Jun;67(6):361-70.

BMJ Publishing Group Limited (BMJ) disclaims all liability and responsibility arising from any reliance placed on this supplemental material which has been supplied by the author(s)

Supplement 3: Breakdown of healthcare professional (HCP) roles for analysis.

**Job title**	
Doctor in research – Gynecology	Split up based on age: trainees/residents and consultants/attending
Doctor in research – Obstetrics	Split up based on age: trainees/residents and consultants/attending
Doctor in research – Obstetrics and Gynecology	Split up based on age: trainees/residents and consultants/attending
Doctor in research – Radiology/Sonologist	Split up based on age: trainees/residents and consultants/attending
Doctor in training – Gynecology	Split up based on age: trainees/residents and consultants/attending
Doctor in training – Obstetrics	Split up based on age: trainees/residents and consultants/attending
Doctor in training – Obstetrics and Gynecology	Split up based on age: trainees/residents and consultants/attending
Doctor in training – Radiologist/Sonologist	Split up based on age: trainees/residents and consultants/attending
Gynecologist	Split up based on age: trainees/residents and consultants/attending
Midwife – Early pregnancy only	Sonographer
Midwife – Obstetrics	Sonographer
Nurse – Early pregnancy only	Sonographer
Nurse – Gynecology	Sonographer
Nurse – Obstetrics	Sonographer
Nurse – Obstetrics and Gynecology	Sonographer
Obstetrician	Split up based on age: trainees/residents and consultants/attending
Obstetrician and Gynecologist	Split up based on age: trainees/residents and consultants/attending
Radiologist/Sonologist	Split up based on age: trainees/residents and consultants/attending
Retired	Exclude from analyses
Sonographer – Gynecology	Sonographer
Sonographer – Obstetrics	Sonographer
Sonographer – Obstetrics and Gynecology	Sonographer

Supplement 4: Breakdown of risk by area of work for analysis.

**Areas of work**	**Risk of COVID-19 infection**
Early pregnancy unit - Ultrasound and assessment	High risk
Gynecology - Acute Gynecology	High risk
Gynecology - Benign Gynecology	Low risk
Gynecology – Oncology	Low risk
Gynecology - Ultrasound	High risk
Obstetrics/Gynecology/Radiology - From home	Low risk
Obstetrics - Antenatal clinic	Moderate risk
Obstetrics - Antenatal ward	Moderate risk
Obstetrics - Birth center/Birthing center	High risk
Obstetrics - Community clinics	Moderate risk
Obstetrics - Labor ward	High risk
Obstetrics - Maternity helplines	Low risk
Obstetrics - Maternity triage/Hospital obstetric triage	High risk
Obstetrics - Postnatal ward	Moderate risk
Obstetrics – Ultrasound	High risk
Radiology - non-Ultrasound	Moderate risk
Radiology - Ultrasound	High risk

Supplement 5: Flowchart summarizing survey participants, differentiating International Society of Ultrasound in Obstetrics and Gynecology (ISUOG) members and non-ISUOG members (n = 3287).

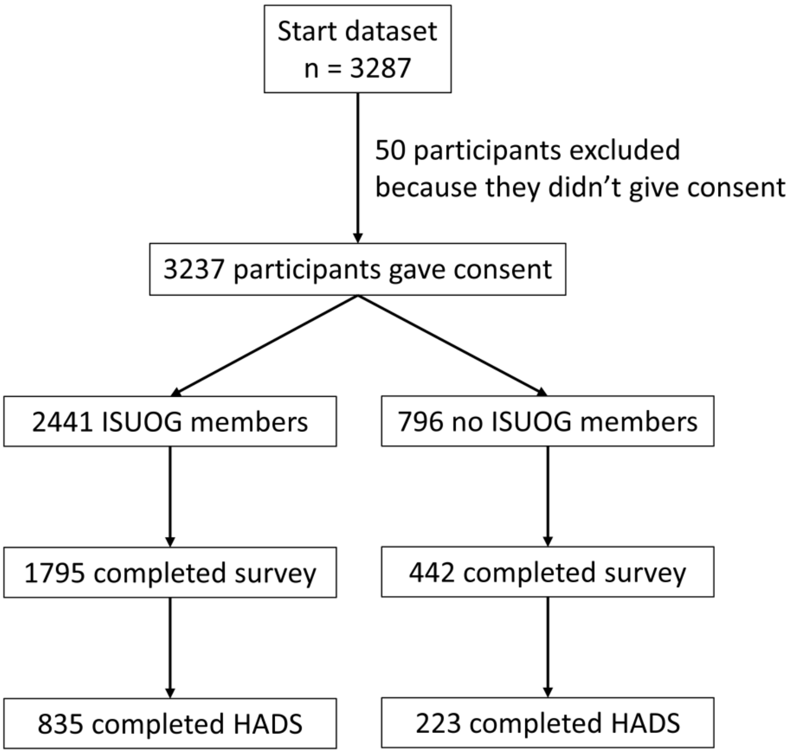

Response rate:
ISUOG members - 23.1% (2441/10589)
Non-ISUOG members - 3.2% (796/24920).
Abbreviations: HADS (Hospital Anxiety and Depression scale)

Supplement 6: Demographics of survey participation, differentiated based on International Society of Ultrasound in Obstetrics and Gynecology (ISUOG) membership (n = 3237).

	ISUOG members (n = 2441)	Others (n = 796)
**Total ISUOG mailing list (n)**	10589	24920
**Response of total ISUOG mailing list (n, %)**	2441 (23%)	796 (3%)
Countries (n)	120	78
Number of respondents in English (n)	1603	357
Number of respondents in Spanish (n)	564	317
Number of respondents in Italian (n)	195	72
Number of respondents in French (n)	79	50
**Completed survey**	**1795**	**442**
**Countries (n)**	110	65
**Age (mean, range)**	47.2 (18-82)	47.7 (25-78)
≤20 years	1 (0%)	0 (0%)
21-30 years	95 (5%)	22 (5%)
31-40 years	454 (25%)	104 (24%)
41-50 years	549 (31%)	137 (31%)
51-60 years	475 (26%)	121 (28%)
61-70 years	201 (11%)	50 (11%)
>70 years	18 (1%)	6 (1%)
**Gender (n, %)**		
Female	1176 (66%)	298 (68%)
Male	613 (34%)	142 (32%)
Intersex	1 (0%)	0 (0%)
Prefer not to say	3 (0%)	0 (0%)
**Prevalence suspected or confirmed SARS-CoV-2 based on symptoms +/- PCR testing (%)**	230 (13%)	60 (14%)
**Is PPE used by respondents (n, %)**		
Not yet	217 (12%)	62 (14%)
Yes - for aerosol generating procedures only	110 (6%)	23 (5%)
Yes - for all patients	1142 (64%)	250 (57%)
Yes - for suspected/positive patients only	324 (18%)	105 (24%)
**What PPE is used by respondents (n, %)**		
None	59 (3%)	17 (4%)
Gloves only	38 (2%)	11 (3%)
Gloves + surgical mask	393 (22%)	104 (24%)
Gloves + surgical mask + visor	116 (6%)	36 (8%)
Gloves + FFP3 mask	83 (5%)	12 (3%)
Gloves + FFP3 mask + visor	39 (2%)	12 (3%)
Gloves + FFP3 mask + visor + gown + surgical hat	70 (4%)	20 (5%)

Patient wearing mask	1482 (83%)	359 (82%)
**Concerns regarding a lack of PPE (n, %)**		
No	475 (26%)	104 (24%)
Gloves	223 (12%)	55 (13%)
Gown	499 (28%)	130 (30%)
N95/FFP3 mask	1014 (57%)	265 (60%)
Surgical hat	187 (10%)	46 (10%)
Surgical mask	657 (37%)	163 (37%)
Visor	458 (26%)	124 (28%)
Surgical mask (patient)	479 (27%)	130 (30%)
**Type of PPE that has been re-used or recycled (n, %)**		
No	690 (38%)	175 (40%)
Gloves	27 (2%)	7 (2%)
Gown	229 (13%)	58 (13%)
N95/FFP3 mask	722 (40%)	164 (37%)
Surgical hat	74 (4%)	18 (4%)
Surgical mask	435 (24%)	130 (30%)
Visor	493 (27%)	128 (29%)
**Anxiety (mean, 95% CI)***	7 (6.7-7.3)	6.6 (6.1-7.1)
0-7 (none)	480 (57%)	140 (63%)
8-10 (mild)	180 (22%)	49 (22%)
11-21 (moderate to severe)	175 (21%)	34 (15%)
**Depression (mean, 95% CI)***	5.4 (5.2-5.7)	4.9 (4.4-5.4)
0-7 (none)	587 (70%)	173 (78%)
8-10 (mild)	171 (20%)	34 (15%)
11-21 (moderate to severe)	77 (9%)	16 (7%)

*Numbers of HADS respondents lower than for main survey (n = 835 ISUOG members, n = 223 Others)

Abbreviations: PCR (Polymerase Chain Reaction); PPE (Personal Protective Equipment)

Supplement 7: Comparison of comorbidity in those with (n = 290) and without suspected or confirmed SARS-CoV-2 (n = 1947) (Total n = 2237).

	Suspected or confirmed SARS-CoV-2	Without suspected or confirmed SARS-CoV-2
Heart disease (n, %)	3 (1%)	43 (2%)
Lung disease (n %)	30 (10%)	160 (8%)
Use of antihypertensives (n, %)	39 (13%)	283 (15%)
Smokers (n, %)	13 (4%)	125 (6%)

Supplement 8: Comparison of Hospital Anxiety and Depression scale (HADS) scores in those with (n = 162) and without suspected or confirmed SARS-CoV-2 (n = 896) (Total n = 1058).

	Suspected or confirmed SARS-CoV-2	Without suspected or confirmed SARS-CoV-2
Anxiety (mean, 95% CI)	7.4 (6.7-8.0)	6.8 (6.6-7.1)
0-7 (none)	86 (53%)	534 (60%)
8-10 (mild)	32 (20%)	197 (22%)
11-21 (moderate to severe)	44 (27%)	165 (18%)
Depression (mean, 95% CI)	5.8 (5.2-6.4)	5.2 (5.0-5.5)
0-7 (none)	105 (65%)	655 (73%)
8-10 (mild)	37 (23%)	168 (19%)
11-21 (moderate to severe)	20 (12%)	73 (8%)

Supplement 9: Hospital Anxiety and Depression scale (HADS) breakdown by gender (n = 1058).

	Female	Male	Prefer not to say
**Number of respondents (n, %)**	754 (71%)	303 (29%)	1 (0%)
**Anxiety (mean, 95% CI)***	6.9 (6.6-7.2)	5.7 (5.3-6.2)	-
0-7 (None) (n, %)	450 (60%)	202 (67%)	1 (100%)
8-10 (Mild) (n, %)	148 (20%)	60 (20%)	0 (0%)
11-21 (Moderate to severe) (n, %)	156 (21%)	41 (14%)	0 (0%)
**Depression (mean, 95% CI)***	5.4 (5.2-5.7)	5.0 (4.6-5.4)	-
0-7 (none) (n, %)	529 (70%)	230 (76%)	1 (100%)
8-10 (mild) (n, %)	146 (19%)	59 (19%)	0 (0%)
11-21 (moderate to severe) (n, %)	79 (10%)	14 (5%)	0 (0%)

Supplement 10: Hospital Anxiety and Depression scale (HADS) breakdown by risk of working area (n = 1058).

	Low risk	Moderate risk	High risk
**Number of respondents (n, %)**	23 (2%)	28 (3%)	1007 (95%)
**Anxiety (mean, 95% CI)***	8.3 (6.3-10.3)	5.1 (3.6-6.6)	6.6 (6.3-6.8)
0-7 (None) (n, %)	13 (57%)	21 (75%)	619 (61%)
8-10 (Mild) (n, %)	3 (13%)	3 (11%)	202 (20%)
11-21 (Moderate to severe) (n, %)	7 (30%)	4 (14%)	186 (18%)
**Depression (mean, 95% CI)***	8.2 (6.0-10.3)	4.3 (2.7-5.8)	5.3 (5.0-5.5)
0-7 (none) (n, %)	12 (52%)	19 (68%)	729 (72%)
8-10 (mild) (n, %)	4 (17%)	6 (21%)	195 (19%)
11-21 (moderate to severe) (n, %)	7 (30%)	3 (11%)	83 (8%)

Supplement 11: Comparison of Hospital Anxiety and Depression scale (HADS) scores in those with (n = 788) and without personal protective equipment (PPE) shortages (n = 270) (Total n = 1058).

	**PPE shortages**	**No PPE shortages**
Anxiety (mean, 95% CI)	7.4 (7.1-7.7)	5.5 (5.0-6.0)
0-7 (none)	430 (55%)	190 (70%)
8-10 (mild)	183 (23%)	46 (17%)
11-21 (moderate to severe)	175 (22%)	34 (13%)
Depression (mean, 95% CI)	5.7 (5.5-6.0)	4.1 (3.7-4.5)
0-7 (none)	540 (69%)	220 (81%)
8-10 (mild)	167 (21%)	38 (14%)
11-21 (moderate to severe)	81 (10%)	12 (4%)
