# Supplementary material for: Experiences and well-being of healthcare professionals working in the field of ultrasound in obstetrics and gynaecology as the SARS-CoV-2 pandemic were evolving: a cross-sectional survey study
Source: BMJ Open. 2022 Feb 4;12(2):e051700. doi: 10.1136/bmjopen-2021-051700 (PMC8819548; doi:10.1136/bmjopen-2021-051700)
Supplement: Author's manuscript [file bmjopen-2021-051700.draft_revisions.pdf]

BMJ Open is committed to open peer review. As part of this commitment we make the peer review history of every article we publish publicly available.

When an article is published we post the peer reviewers' comments and the authors' responses online. We also post the versions of the paper that were used during peer review. These are the versions that the peer review comments apply to.

The versions of the paper that follow are the versions that were submitted during the peer review process. They are not the versions of record or the final published versions. They should not be cited or distributed as the published version of this manuscript.

BMJ Open is an open access journal and the full, final, typeset and author-corrected version of record of the manuscript is available on our site with no access controls, subscription charges or pay-per-view fees (http://bmjopen.bmj.com).

If you have any questions on BMJ Open's open peer review process please email info.bmjopen@bmj.com

BMJ Open

The experiences and wellbeing of healthcare professionals working in the field of ultrasound in Obstetrics and Gynecology as the SARS-CoV-2 pandemic was evolving: a cross-sectional survey study

Journal:	*BMJ Open*
Manuscript ID	bmjopen-2021-051700
Article Type:	Original research
Date Submitted by the Author:	12-Apr-2021

Complete List of Authors:	Bourne, Tom; Imperial College London, Early Pregnancy and Acute Gynaecology Unit, Department of Obstetrics and Gynaecology, Queen Charlotte's and Chelsea Hospital; KU Leuven, Department of Development and Regeneration<br>Kyriacou, Christopher; Imperial College London, Early Pregnancy and Acute Gynaecology Unit, Department of Obstetrics and Gynaecology, Queen Charlotte's and Chelsea Hospital<br>Shah, Harsha; Imperial College London, Early Pregnancy and Acute Gynaecology Unit, Department of Obstetrics and Gynaecology, Queen Charlotte's and Chelsea Hospital<br>Ceusters, Jolien; KU Leuven, Department of Oncology, Laboratory of Tumour Immunology and Immunotherapy<br>Preisler, Jessica; Universidad del Desarrollo, Facultad de Medicina, Clínica Alemana ; Hospital Clínico de la Universidad de Chile José Joaquín Aguirre, Facultad de Medicina, Clínica Alemana<br>Metzger, Ulrike; Centre d'Échographie de l'Odéon<br>Landolfo, Chiara; Fondazione Policlinico Universitario Agostino Gemelli IRCCS, Dipartimento Scienze della Salute della Donna, del Bambino e di Sanità Pubblica<br>Lees, Christoph; Imperial College London, Centre for Fetal Care, Department of Obstetrics and Gynaecology, Queen Charlotte's and Chelsea Hospital<br>Timmerman, Dirk; KU Leuven, Department of Development and Regeneration; KU Leuven University Hospitals Leuven, Department of Obstetrics and Gynaecology

Keywords:	Ultrasound < RADIOLOGY & IMAGING, ULTRASONOGRAPHY, GYNAECOLOGY, Ultrasonography < OBSTETRICS, Public health < INFECTIOUS DISEASES

SCHOLARONE™
Manuscripts

I, the Submitting Author has the right to grant and does grant on behalf of all authors of the Work (as defined in the below author licence), an exclusive licence and/or a non-exclusive licence for contributions from authors who are: i) UK Crown employees; ii) where BMJ has agreed a CC-BY licence shall apply, and/or iii) in accordance with the terms applicable for US Federal Government officers or employees acting as part of their official duties; on a worldwide, perpetual, irrevocable, royalty-free basis to BMJ Publishing Group Ltd ("BMJ") its licensees and where the relevant Journal is co-owned by BMJ to the co-owners of the Journal, to publish the Work in this journal and any other BMJ products and to exploit all rights, as set out in our licence.

The Submitting Author accepts and understands that any supply made under these terms is made by BMJ to the Submitting Author unless you are acting as an employee on behalf of your employer or a postgraduate student of an affiliated institution which is paying any applicable article publishing charge ("APC") for Open Access articles. Where the Submitting Author wishes to make the Work available on an Open Access basis (and intends to pay the relevant APC), the terms of reuse of such Open Access shall be governed by a Creative Commons licence – details of these licences and which Creative Commons licence will apply to this Work are set out in our licence referred to above.

Other than as permitted in any relevant BMJ Author's Self Archiving Policies, I confirm this Work has not been accepted for publication elsewhere, is not being considered for publication elsewhere and does not duplicate material already published. I confirm all authors consent to publication of this Work and authorise the granting of this licence.

For peer review only

TITLE PAGE

The experiences and wellbeing of healthcare professionals working in the field of ultrasound in Obstetrics and Gynecology as the SARS-CoV-2 pandemic was evolving: a cross-sectional survey study

Authors

Tom Bourne[1,2], Chris Kyriacou[1], Harsha Shah[1], Jolien Ceusters[3], Jessica Preisler[4,5], Ulrike Metzger[6], Chiara Landolfo[7], Christoph Lees[8], Dirk Timmerman[2,9]

Affiliations

1. Early pregnancy and Acute Gynaecology Unit, Department of Obstetrics and Gynaecology, Queen Charlotte's and Chelsea Hospital, Imperial College, London, UK.
2. Department of Development and Regeneration, KU Leuven, Leuven, Belgium.
3. Department of Oncology, Laboratory of Tumour Immunology and Immunotherapy, KU Leuven, Leuven, Belgium.
4. Facultad de Medicina, Clínica Alemana – Universidad del Desarrollo, Santiago, Chile
5. Hospital Clínico de la Universidad de Chile, Santiago, Chile.
6. Centre d'Échographie de l'Odéon, Paris, France.
7. Dipartimento Scienze della Salute della Donna, del Bambino e di Sanità Pubblica, Fondazione Policlinico Universitario Agostino Gemelli, IRCCS, Rome, Italy.
8. Centre for Fetal Care, Department of Obstetrics and Gynaecology, Queen Charlotte's and Chelsea Hospital, Imperial College, London, UK.
9. Department of Obstetrics and Gynaecology, University Hospitals Leuven, Leuven, Belgium.

Correspondence to:

Professor Tom Bourne
Queen Charlotte's and Chelsea Hospital, Imperial College London
Du Cane Road, London, W12 0HS, UK
t.bourne@imperial.ac.uk

Word count: 3852

For peer review only

ABSTRACT

Objectives: Assess experience of healthcare professionals (HCP) working with ultrasound in Obstetrics and Gynecology during the evolving SARS-CoV-2 pandemic.

Design: Prospective cross-sectional study.

Setting: Online international survey available in four languages assessing working arrangements, physical and psychological wellbeing via single-best, open box and Hospital and Anxiety Depression Scale (HADS) questions.

Participants: Mailing list members of the International Society of Ultrasound in Obstetrics and Gynecology (ISUOG) were emailed the survey, which was open for two weeks in May 2020. 2237 of 3237 (69.1%) HCPs from 115 countries who consented to participate completed the survey. 1058 (47.3%) completed the HADS.

Primary outcome measures: SARS-CoV-2 prevalence, depression and anxiety.

Results: Confirmed/suspected SARS-CoV-2 prevalence was 13.0%. Concerns about personal protective equipment (PPE) provision were raised by 74.1% of participants; highest amongst trainees/resident physicians (83.9%), and amongst HCPs in Spain (89.7%). Most participants worked in self-perceived high-risk areas for SARS-CoV-2 (67.5-87.0%), with proportionately more trainees interacting with suspected/confirmed infected patients (57.1% versus 24.2-40.6%) and sonographers seeing more patients who did not wear a mask (33.3% versus 13.9-7.9%). The most frequent PPE combination used were gloves and a surgical mask (22.3%). UK and US respondents reported spending less time self-isolating (8.8 days) and lower satisfaction with their national pandemic response (37.0-43.0%). 19.8% and 8.8% of respondents met the criteria for moderate to severe anxiety and depression respectively.

Conclusions: Reported SARS-CoV-2 HCP prevalence is consistent with literature findings. Most respondents used gloves and a surgical mask, with a greater SARS-CoV-2 prevalence compared with those using ‘full’ PPE. HCPs with the least agency (trainees and sonographers) were not only more likely to see high-risk patients but less likely to be protected. A fifth of respondents reported moderate to severe anxiety.

Trial registration: Ethical approval received from the ethics committee at KU Leuven, Belgium (S-64016).

Keywords: SARS-CoV-2, healthcare professional, ultrasound, anxiety, depression.

STRENGTHS AND LIMITATIONS OF THIS STUDY

- Strengths of our study include the sample size, the assessment of HCP responses internationally and the ability to compare responses between different HCPs.
- Four languages enabled greater participant inclusion and responses between ISUOG and non-ISUOG members were compared prior to grouping for analysis.
- Key weaknesses include possible selection bias due to the method of sample selection and the low population response rate.
- It is important not to draw conclusions about causation as this is a descriptive study performed as the pandemic was evolving.
- At the time of survey participation, the majority of HCP did not have the same access to a swab or antibody test that is now available.

For peer review only

INTRODUCTION

Background: The severe acute respiratory syndrome coronavirus 2 (SARS-CoV-2) pandemic has caused unprecedented challenges to all healthcare systems(1). These include patient protection, managing the burden of disease on service capacity and mitigating adverse effects on healthcare professionals (HCP: physicians, sonographers, nurses and allied HCP)(2).

Guidance internationally has focused on clinician redeployment to front-line settings, prioritization of services, provision and rationalization of personal protective equipment (PPE)(3, 4). During the pandemic, access to PPE for HCP has remained a key concern. Despite HCP being prioritized in most countries, PPE shortages have been described universally(1), and were a particular concern in the early months of the pandemic. Lack of sufficient and adequate PPE is important in Obstetrics and Gynecology where HCP work in a variety of settings, including ultrasound scanning, which may not ordinarily be considered high-risk but involves close patient proximity for extended periods(5). These areas are easily over-looked but may pose a high-risk of SARS-CoV-2 infection(6).

A further concern has been the availability of SARS-CoV-2 testing amongst HCP. Initially, provision for this was variable, with for example relatively easy access in Hong Kong compared to virtually none in the UK. This is important as, in the absence of testing, HCP risk becoming COVID-19 vectors within hospitals and the wider community. Lack of testing may also lead to unnecessary isolation from work. There is limited data regarding HCP testing availability and time HCP have taken off work due to suspected or confirmed SARS-CoV-2(7).

Less attention has been focused on the implications of infectious diseases on HCP psychological health, which previous pandemics have shown to be significant(8, 9). HCP are confronted with ongoing resource, shift pattern, PPE and testing uncertainty(1, 10), as well as COVID-19 exposure risk and the implications of this on their families(11). Protecting the psychological health of the medical workforce is critical, particularly as anxiety, depression and burnout are recognized complications for HCP working in high stress environments(12).

For peer review only

Objectives: We aim to assess the scale, experience and psychological wellbeing of clinicians who have experienced the evolving SARS-CoV-2 pandemic working with ultrasound in Obstetrics and Gynecology internationally between 7th-21st May 2020.

For peer review only

METHODS

Study design: We used a cross-sectional survey design with two main sections assessing physical aspects, including underlying medical conditions, comorbidities and personal SARS-CoV-2 experience, and psychological wellbeing. Participants were initially asked to provide informed consent prior to continuing to the online survey. If they did not give consent by answering 'yes' to the first question, they were not directed to the survey. Single-best answer and open box questions were proposed by a senior gynaecologist. These were then piloted, vetted and modified by the authors before being translated from English to French, Spanish and Italian in order to increase survey accessibility. We estimated that the time taken to complete the questionnaire was 10 minutes.

Setting: The online platform SurveyMonkey (2020) was selected to upload and disseminate the questionnaire, which was encrypted, and multiple responses were disabled. The survey design ensured the data collected was completely anonymous for all respondents.

Participants: All HCP's on the ISUOG mailing list were invited to participate in this study between 7th and 21st May 2020. There were 1,237,953 confirmed cases of SARS-CoV-2 infection and 63,313 deaths in the time the survey was open(13). Various countries had instigated local and national lockdowns at this time, including the UK. Members were sent an email containing information describing the study and individual links to the questionnaire in English, Spanish, Italian and French. We made it clear to the participants that their participation was voluntary and that responses would be both anonymous and untraceable.

During the survey period, two reminders were sent out. As this was a survey study of a relatively new condition at the time, a power calculation was not performed. Exclusion criteria included retired doctors as this was deemed to not be reflective of the working environment during the SARS-CoV-2 pandemic. A copy of the questionnaire in English can be viewed in Supplementary Material 1.

For peer review only

Patient and Public Involvement statement: Given the HCP participant population and the acute nature of the SARS-CoV-2 pandemic, patient and public involvement was not performed.

Variables and data sources

Physical aspects: This section was divided into sub-sections: Introduction, Demographics, Underlying medical conditions, Medications, Smoking and Body Mass Index (BMI), Role, Shift pattern and PPE, SARS-CoV-2 diagnosis, Personal SARS-CoV-2 pathway, Household, Colleagues and Support.

Participants were asked details about their job and organizational attributes, as well as on a variety of personal comorbidities relevant to SARS-CoV-2 infection (14). They were then questioned on their personal experience of SARS-CoV-2 at home and in the workplace.

Psychological Wellbeing: Hospital Anxiety and Depression Scale (HADS): Participants were given the option to complete the validated Hospital Anxiety and Depression Scale (HADS), which is a 14-item (7 questions related to anxiety and depression each) questionnaire (15). Each subscale measures symptom severity (the score ranges between 0 and 21, a score greater or equal to 11 indicates moderate-to-severe symptoms). If respondents chose not to complete the HADS, the survey ended.

Ethical approval: Ethical approval for the distribution of this online survey was received from the ethics committee at KU Leuven, Belgium (S-64016). A copy of the protocol can be viewed in Supplementary Material 2.

Outcome measures: The primary outcome was the overall prevalence of SARS-CoV-2, depression and anxiety amongst HCPs in relation to country and PPE availability.

Statistical analyses: Univariate analysis was used to investigate the association between country of origin, type of HCP and PPE with results presented as percentages (%), mean, 95% confidence intervals, medians and ranges depending on the variable. Missing data was not included in the analysis.

For peer review only

HCPs and areas of work were divided into groups. For HCPs, there were three main categories: Consultants/Attending physicians (Gynaecologists, Obstetricians, Obstetricians and Gynaecologists or Radiologists/Sonologists aged 35 years and older), Trainees/Residents (Gynaecologists, Obstetricians, Obstetricians and Gynaecologists or Radiologists/Sonologists aged 34 years and younger, doctors in research, doctors in training) and Sonographers (sonographers and allied healthcare professionals [Midwives and Nurses]) (Supplementary Material 3). Areas of work were defined as high-risk (gynaecology [early pregnancy unit, acute gynaecology, ultrasound], obstetrics [birth centre/birthing centre, labor ward, maternity triage/hospital obstetric triage, ultrasound], radiology [sonology-ultrasound]), moderate risk (obstetrics [antenatal clinic, antenatal ward, community clinics, postnatal ward], radiology [non-ultrasound]), or low-risk (working from home, gynecology [benign, oncology], obstetrics [maternity helplines]) (Supplementary Material 4). This categorisation approximated risk based on HCP proximity and time spent with patients, allowing meaningful data analysis.

Anxiety and depression scores were divided by case-ness in order to differentiate the proportion of the study population that had no anxiety or depression (HADS 0-7), those experiencing mild anxiety or depression (HADS 8-10) and those suffering moderate to severe anxiety or depression (HADS 11-21).

For peer review only

RESULTS

Summary

Response: The survey was sent to 35,509 HCPs in 124 countries. 3287 (9.3%) HCPs clicked the survey link and 3237 (98.5%) consented to complete it. 1960 (60.5%) responded in English, 881 (27.2%) in Spanish, 267 (8.2%) in Italian and 129 (4.0%) in French. The response rate for ISUOG members was 23.1% (2441/10589) whist non-ISUOG members was 3.2% (796/24920) (Supplementary Material 5). 2237 of 3237 (69.1%) HCPs from 115 countries who started the survey completed it in full. The age range of respondents was from 18 to 82, and 66% of respondents were female. 1058/2237 participants (47.3%) completed the HADS. Survey participation and demographics are summarised in Table 1.

Table 1: Demographics of total survey participation (n = 3237).

	**Total (n = 3237)**
**Total ISUOG mailing list (n)**	35509
**Response of total ISUOG mailing list (n, %)**	3237 (9%)
Countries (n)	124
Number of respondents in English (n)	1960
Number of respondents in Spanish (n)	881
Number of respondents in Italian (n)	267
Number of respondents in French (n)	129
ISUOG members (n, %)	2441 (23%)
**Completed survey**	**2237**
**ISUOG members (n, %)**	1795 (80%)
**Countries (n)**	115
**Age (mean, range)**	47.2 (18-82)
**Gender (n, %)**	
Female	1474 (66%)
Male	755 (34%)
Intersex	1 (0%)
Prefer not to say	3 (0%)
**Prevalence suspected/confirmed SARS-CoV-2 based on symptoms +/- PCR testing (n, %)**	290 (13%)
**Is PPE used by respondents (n, %)**	
Not yet	279 (12%)
Yes - for aerosol generating procedures only	133 (6%)
Yes - for all patients	1392 (62%)
Yes - for suspected/positive patients only	429 (19%)
**What PPE is used by respondents (n, %)**	
None	76 (3%)

For peer review only

Gloves only	49 (2%)
Gloves + surgical mask	497 (22%)
Gloves + surgical mask + visor	152 (7%)
Gloves + FFP3 mask	95 (4%)
Gloves + FFP3 mask + visor	51 (2%)
Gloves + FFP3 mask + visor + gown + surgical hat	90 (4%)
Patient wearing mask	1841 (82%)
**Concerns regarding a lack of PPE (n, %)**	
No	579 (26%)
Gloves	278 (12%)
Gown	629 (28%)
N95/FFP3 mask	1279 (57%)
Surgical hat	233 (10%)
Surgical mask	820 (37%)
Visor	582 (26%)
Surgical mask (patient)	609 (27%)
**Type of PPE that has been re-used or recycled (n, %)**	
No	865 (39%)
Gloves	34 (2%)
Gown	287 (13%)
N95/FFP3 mask	886 (40%)
Surgical hat	92 (4%)
Surgical mask	565 (25%)
Visor	621 (28%)
**Anxiety (mean, 95% CI)***	6.9 (6.7-7.2)
0-7 (none)	620 (59%)
8-10 (mild)	229 (22%)
11-21 (moderate to severe)	209 (20%)
**Depression (mean, 95% CI)***	5.3 (5.1-5.5)
0-7 (none)	760 (72%)
8-10 (mild)	205 (19%)
11-21 (moderate to severe)	93 (9%)

*Numbers of HADS respondents lower than main survey (n = 1058).

Direct comparison of data between the characteristics of ISUOG members and non-ISUOG members confirmed findings were similar (Supplementary Material 6). It was therefore deemed appropriate to combine these populations for the analysis.

For peer review only

COVID-19 prevalence: Confirmed or suspected SARS-CoV-2 prevalence was 13.0% (290/2237). Amongst this group, 11.4% had a history of heart/lung disease in, 13.4% were taking antihypertensives and 4.5% were smokers (Supplementary Material 7).

PPE use and provision: 12.5% of all HCPs reported not using PPE whilst 87.5% reported variable use. 74.1% had concerns about provision or shortages of one or more items of PPE, in particular N95/FFP3 masks (57.2%). PPE was reported to be recycled or re-used by 61.3%.

Anxiety and depression: 21.6% of participants met the criteria for mild anxiety and 19.8% for moderate to severe anxiety. 19.4% met the criteria for mild and 8.8% for moderate to severe depression. 27.2% and 12.3% of those with suspected/confirmed SARS-CoV-2 reported moderate to severe anxiety and depression, compared to 18.4% and 8.1% of those without (Supplementary Material 8).

4 out of 2237 participants were excluded from the univariate analyses, having completed the survey following retirement.

Univariate analysis by country (n=2233)

A breakdown of findings by countries that had most respondents are presented in Table 2.

Table 2: Breakdown of findings by country (n = 2233).

	Italy	Spain	India	Philippines	UK	US	Other^
**Number of respondents (n, %)**	174 (8%)	136 (6%)	115 (5%)	114 (5%)	103 (5%)	102 (5%)	1489 (67%)
**Prevalence suspected/ confirmed SARS-CoV-2 based on symptoms +/- PCR testing (n, %)**	26 (15%)	20 (15%)	3 (3%)	10 (9%)	32 (31%)	14 (14%)	185 (12%)
**Number who had SARS-CoV-2 antibody test (n, %)**	92 (53%)	82 (60%)	1 (1%)	21 (18%)	1 (1%)	16 (16%)	155 (10%)
**Number who had positive SARS-CoV-2 antibody (n, %)**	6 (7%)	11 (13%)	0 (0%)	0 (0%)	0 (0%)	4 (25%)	16 (10%)
**Is PPE used by respondents (n, %)**							
Not yet	17 (10%)	28 (21%)	31 (27%)	6 (5%)	7 (7%)	5 (5%)	185 (12%)
Yes – for aerosol generating procedures only	8 (5%)	6 (4%)	9 (8%)	7 (6%)	6 (6%)	3 (3%)	94 (6%)
Yes – for all patients	107 (61%)	33 (24%)	65 (57%)	96 (84%)	76 (74%)	83 (81%)	932 (63%)

For peer review only

Yes – for suspected/positive patients only	42 (24%)	69 (51%)	10 (9%)	5 (4%)	14 (14%)	11 (11%)	278 (19%)
**What PPE is used by respondents (n, %)**							
None	0 (0%)	1 (1%)	1 (1%)	1 (1%)	1 (1%)	0 (0%)	72 (5%)
Gloves only	0 (0%)	0 (0%)	0 (0%)	0 (0%)	2 (2%)	0 (0%)	47 (3%)
Gloves + surgical mask	79 (45%)	42 (31%)	7 (6%)	0 (0%)	41 (40%)	45 (44%)	283 (19%)
Gloves + surgical mask + visor	6 (3%)	11 (8%)	6 (5%)	1 (1%)	17 (17%)	12 (12%)	99 (7%)
Gloves + FFP3 mask	12 (7%)	10 (7%)	8 (7%)	0 (0%)	0 (0%)	6 (6%)	59 (4%)
Gloves + FFP3 mask + visor	1 (1%)	4 (3%)	6 (5%)	2 (2%)	0 (0%)	3 (3%)	35 (2%)
Gloves + FFP3 mask + visor + gown + surgical hat	3 (2%)	5 (4%)	13 (11%)	23 (20%)	0 (0%)	1 (1%)	45 (3%)
Patient wearing mask	173 (99%)	133 (98%)	109 (95%)	111 (97%)	33 (32%)	91 (89%)	1191 (80%)
**Concerns regarding a lack of PPE (n, %)**							
No	35 (20%)	14 (10%)	52 (45%)	20 (18%)	46 (45%)	24 (24%)	388 (26%)
Gloves	25 (14%)	19 (14%)	5 (4%)	12 (11%)	1 (1%)	9 (9%)	207 (14%)
Gown	53 (30%)	45 (33%)	23 (20%)	54 (47%)	25 (24%)	23 (23%)	406 (27%)
N95/FFP3 mask	118 (68%)	110 (81%)	51 (44%)	84 (74%)	33 (32%)	61 (60%)	822 (55%)
Surgical hat	15 (9%)	10 (7%)	10 (9%)	15 (13%)	4 (4%)	8 (8%)	171 (11%)
Surgical mask	84 (48%)	62 (46%)	10 (9%)	21 (18%)	24 (23%)	45 (44%)	574 (39%)
Visor	51 (29%)	51 (38%)	11 (10%)	19 (17%)	19 (18%)	24 (24%)	407 (27%)
Surgical mask (patient)	44 (25%)	47 (35%)	10 (9%)	32 (28%)	13 (13%)	31 (30%)	432 (29%)
**Type of PPE that has been re-used or recycled (n, %)**							
No	55 (32%)	31 (23%)	40 (35%)	25 (22%)	62 (60%)	12 (12%)	640 (43%)
Gloves	2 (1%)	3 (2%)	5 (4%)	2 (2%)	0 (0%)	1 (1%)	21 (1%)
Gown	15 (9%)	17 (13%)	23 (20%)	70 (61%)	3 (3%)	4 (4%)	155 (10%)
N95/FFP3 mask	82 (47%)	82 (60%)	59 (51%)	66 (58%)	10 (10%)	68 (67%)	519 (35%)
Surgical hat	2 (1%)	7 (5%)	1 (1%)	16 (14%)	1 (1%)	4 (4%)	61 (4%)
Surgical mask	83 (48%)	60 (44%)	7 (6%)	13 (11%)	14 (14%)	65 (64%)	323 (22%)
Visor	23 (13%)	48 (35%)	31 (27%)	56 (49%)	28 (27%)	28 (27%)	407 (27%)
**Mean days of self-isolation (range)**	11.3 (0-30)	11.6 (0-30)	17.3 (0-30)	16.9 (0-30)	8.8 (0-30)	8.8 (0-30)	8.4 (0-30)
**Mean days of household self-isolating (range)**	21.1 (14-30)	10.5 (2-15)	18 (10-30)	15.3 (7-30)	11.1 (2-14)	15 (2-30)	12.2 (1-30)
**Number of respondents satisfied with local unit SARS-CoV-2 response (%)**	89 (51%)	97 (71%)	89 (77%)	89 (78%)	75 (73%)	74 (73%)	1018 (68%)
**Mean % level of respondent satisfaction with government SARS-CoV-2 response (95% CI)**	38 (34-41)	23 (19-26)	61 (56-65)	48 (44-52)	43 (38-48)	37 (31-43)	53 (52-55)
**Anxiety (mean, 95% CI)***	6.3 (5.6-7.1)	6.9 (6.1-7.7)	7.4 (6.1-8.8)	7.1 (5.6-8.6)	7.2 (6.2-8.2)	7.3 (6.1-8.4)	6.9 (6.6-7.2)
0-7 (none)	74 (71%)	51 (61%)	22 (51%)	18 (50%)	36 (58%)	35 (59%)	384 (57%)

For peer review only

8-10 (mild)	13 (12%)	21 (25%)	13 (30%)	10 (28%)	12 (19%)	8 (14%)	152 (23%)
11-21 (moderate to severe)	17 (16%)	12 (14%)	8 (19%)	8 (22%)	14 (23%)	16 (27%)	134 (20%)
**Depression (mean, 95% CI)***	5.8 (5.2-6.5)	5 (4.2-5.7)	5.1 (4.1-6.1)	5.3 (4.2-6.5)	5.3 (4.3-6.2)	4.8 (3.8-5.7)	5.3 (5-5.6)
0-7 (none)	74 (71%)	67 (80%)	31 (72%)	27 (75%)	44 (71%)	46 (78%)	471 (70%)
8-10 (mild)	20 (19%)	11 (13%)	10 (23%)	8 (22%)	12 (19%)	9 (15%)	135 (20%)
11-21 (moderate to severe)	10 (10%)	6 (7%)	2 (5%)	1 (3%)	6 (10%)	4 (7%)	64 (10%)

^The 1489 within the 'Other' cohort make up data from participants of the remaining 109 countries. We provide a breakdown of the 6 countries that had the most respondents.
*Numbers of HADS respondents lower than main survey (n Italy = 104, Spain = 84, India = 43, Philippines = 36, UK = 62, US = 59).

COVID-19 prevalence: The highest prevalence of SARS-CoV-2 infection on the basis of symptoms and/or a positive polymerase chain reaction (PCR) swab was reported in the United Kingdom (UK) (31.1%), followed by Italy (14.9%), Spain (14.7%), and the United States (US) (13.7%). Respondents from India and the Philippines reported a prevalence of 2.6% and 8.8% respectively. Only 0.9% of Indian and 1.0% of UK participants had an antibody test, compared with 52.9% and 60.3% of participants in Italy and Spain respectively. 25.0% of participants from the US and 13.4% from Spain who had a SARS-CoV-2 antibody test received a positive result.

Participants in the UK reported spending an average of 8.8 days self-isolating away from the workplace – the same as the US. Mean self-isolation time was only greater than 2 weeks in India and the Philippines (17.3 and 16.9 days respectively) where SARS-CoV-2 prevalence amongst HCP in the study was lowest at the time of the study.

PPE use and provision: The use of PPE when performing ultrasound was reported by 73.0 to 95.1% of participants. The most common combination of PPE used whilst performing ultrasonography was gloves and a surgical mask, with the highest rates of use in Italy (45.4%), the UK (39.8%) and US (44.1%). No participants from the Philippines and only 6.1% from India reported using only gloves and a surgical mask. Visor use in addition to gloves and a surgical mask were highest in UK (16.5%) and US (11.8%). Full PPE (gloves, FFP3/N95 mask, visor, gown, surgical hat) was used by 4% (90/2233) of respondents. Full PPE use was highest in the Philippines (20.2%) and lowest in the UK (0%). With the exception of the UK

For peer review only

(32.0%), high rates of patients wearing a mask were noted in every other country analysed (80.0 to 99.4%).

PPE provision or supply concerns were highest in Spain (89.7%), Italy (79.9%) and the Philippines (82.5%) and lowest in the UK (55.3%) and India (54.8%). Less PPE was reported as being recycled or reused in the UK (39.8%) compared to any other country analysed. In the US, 88.2% of HCPs reported PPE recycling or re-use.

Anxiety, depression and support: The HADS revealed that in the UK 22.6%, and in the US 27.1% of participants met the criteria for moderate to severe anxiety, higher than any other country analysed, with Spanish participants having the lowest rate (14.3%). Italian, UK and 'other countries' participants had the highest rates of moderate to severe depression (9.6-9.7%) with Philippines HCPs having the lowest rates (2.8%).

Levels of satisfaction with the support offered by local units during the pandemic ranged from 68% to 78% in all countries except Italy where the level was markedly lower (51.1%). Indians had the highest satisfaction rate with their government response to the pandemic with a mean of 61%, compared with 43% in the UK, 37% in the US, 38% in Italy, and 23% in Spain.

Univariate analysis by HCP (n=2233)

Table 3 focuses on HCPs – 66.3% consultants/attending physicians, 22.3% sonographers and 11.4% trainee/resident physicians (who are the youngest cohort overall). 62.4-63.3% of physicians and 54.3-55.6% of sonographers reported changes to their duties and patient contact throughout the pandemic.

Table 3: Breakdown of findings by healthcare professional (HCP) (n = 2233).

	Consultants / Attending	Sonographer	Trainees / Residents
**Number of respondents (n, %)**	1480 (66%)	499 (22%)	254 (11%)
**Changes in shift pattern as a result of the pandemic (n, %)**			
Unable to work	34 (2%)	21 (4%)	11 (4%)
Change in duties due to SARS-CoV-2 pandemic			
Increased work	185 (13%)	64 (13%)	37 (15%)

For peer review only

Similar work	570 (39%)	228 (46%)	82 (32%)
Decreased work	691 (47%)	186 (37%)	124 (49%)
Change in patient contact due to the SARS-CoV-2 pandemic			
Increased patient contact	114 (8%)	35 (7%)	22 (9%)
Similar patient contact	530 (36%)	212 (42%)	90 (35%)
Decreased patient contact	743 (50%)	214 (43%)	119 (47%)
No patient contact	59 (4%)	17 (3%)	12 (5%)
**Level of COVID-19 risk of working environment reported by HCP's (n, %)**			
High risk of COVID-19 infection	1144 (77%)	337 (68%)	221 (87%)
Moderate risk of COVID-19 infection	128 (9%)	32 (6%)	14 (6%)
Low risk of COVID-19 infection	208 (14%)	130 (26%)	19 (7%)
**Interaction w suspected/confirmed SARS-CoV-2 patients (n, %)**	601 (41%)	121 (24%)	145 (57%)
**Is PPE used by respondents (n, %)**			
Not yet	171 (12%)	75 (15%)	33 (13%)
Yes - for aerosol generating procedures only	89 (6%)	28 (6%)	16 (6%)
Yes - for all patients	934 (63%)	334 (67%)	124 (49%)
Yes - for suspected/positive patients only	286 (19%)	62 (12%)	81 (32%)
**What PPE is used by respondents (n, %)**			
None	47 (3%)	21 (4%)	8 (3%)
Gloves only	20 (1%)	25 (5%)	4 (2%)
Gloves + surgical mask	315 (21%)	105 (21%)	77 (30%)
Gloves + surgical mask + visor	111 (8%)	35 (7%)	6 (2%)
Gloves + FFP3 mask	70 (5%)	13 (3%)	12 (5%)
Gloves + FFP3 mask + visor	41 (3%)	8 (2%)	2 (1%)
Gloves + FFP3 mask + visor + gown + surgical hat	59 (4%)	24 (5%)	7 (3%)
Patient wearing mask	1274 (86%)	333 (67%)	234 (92%)
**Concerns regarding a lack of PPE (n, %)**			
No	398 (27%)	140 (28%)	41 (16%)
Gloves	176 (12%)	63 (13%)	39 (15%)
Gown	403 (27%)	133 (27%)	93 (37%)
N95/FFP3 mask	860 (58%)	239 (48%)	180 (71%)
Surgical hat	142 (10%)	61 (12%)	30 (12%)
Surgical mask	516 (35%)	189 (38%)	115 (45%)
Visor	379 (26%)	122 (24%)	81 (32%)
Surgical mask (patient)	395 (27%)	133 (27%)	81 (32%)
**Type of PPE that has been re-used or recycled (n, %)**			
No	594 (40%)	205 (41%)	66 (26%)
Gloves	22 (1%)	9 (2%)	3 (1%)
Gown	166 (11%)	92 (18%)	29 (11%)

For peer review only

N95/FFP3 mask	596 (40%)	152 (30%)	138 (54%)
Surgical hat	59 (4%)	22 (4%)	11 (4%)
Surgical mask	357 (24%)	116 (23%)	92 (36%)
Visor	401 (27%)	138 (28%)	82 (32%)
**Prevalence suspected/confirmed SARS-CoV-2 based on symptoms +/- PCR testing (n, %)**	187 (13%)	71 (14%)	32 (13%)
**Number who had SARS-CoV-2 antibody test (n, %)**	259 (18%)	55 (11%)	54 (21%)
**Number who had positive SARS-CoV-2 antibody (n, %)**	28 (11%)	5 (9%)	4 (7%)
**Mean days of self-isolation (range)**	9.6 (0-30)	9.7 (0-30)	8.5 (0-30)
**Mean days of household self-isolating (range)**	13 (1-30)	14.3 (2-30)	8.8 (2-14)
**Number of respondents satisfied with local unit SARS-CoV-2 response (%)**	1043 (70%)	340 (68%)	148 (58%)
**Mean % level of respondent satisfaction with government SARS-CoV-2 response (95% CI)**	47 (46-49)	57 (54-59)	44 (40-47)
**Anxiety (mean, 95% CI)***	6.7 (6.4-7)	7.7 (7.1-8.3)	6.9 (6.3-7.6)
0-7 (None)	433 (61%)	112 (52%)	75 (58%)
8-10 (Mild)	151 (21%)	48 (22%)	30 (23%)
11-21 (Moderate to severe)	131 (18%)	54 (25%)	24 (19%)
**Depression (mean, 95% CI)***	5.3 (5-5.5)	5.5 (5-6.1)	5.3 (4.7-5.9)
0-7 (none)	523 (73%)	148 (69%)	89 (69%)
8-10 (mild)	126 (18%)	50 (23%)	29 (22%)
11-21 (moderate to severe)	66 (9%)	16 (7%)	11 (9%)

*Numbers of HADS respondents lower than main survey (Consultant/Attending = 715, Sonographer = 214, Trainees = 129)

COVID-19 prevalence: Suspected or confirmed SARS-CoV-2 prevalence was similar across the groups (12.6 to 14.2%). 87.0% of trainees worked in high-risk areas compared to 77.3% of consultants and 67.5% of sonographers. However, trainees self-isolated for 1 day less on average (8.5 days) compared to consultants and sonographers (9.6 and 9.7 days respectively).

PPE use and provision: The PPE reported in each HCP group as most frequently used when performing ultrasonography was gloves and a surgical mask (21.0-30.3%), with 11.6-15.0% reporting no PPE use. Only 66.7% of patients wore a mask during interactions with sonographers compared with 87.0% when interacting with physicians.

For peer review only

A larger proportion of trainees (57.1%) interacted with suspected or confirmed patients with SARS-CoV-2 compared to consultants (40.6%) and sonographers (24.2%) but were less likely to use PPE for all patients (48.8%), compared with consultants (63.1%) and sonographers (66.9%).

83.9% of trainees reported PPE concerns (compared with 73.1% of consultants and 71.9% of sonographers), with 74.0% re-using or recycling PPE (compared with 59.9% consultants and 58.9% sonographers).

Anxiety, depression and support: Rates of anxiety (39.4-47.7%) and depression (26.9-31.0%) were similar across the HCP groups, with 18.3-25.2% and 7.5-9.2% of participants meeting the criteria for moderate to severe anxiety and depression respectively.

Trainees reported lower satisfaction/support from their local unit (58.3%, compared with 70.5% and 68.1% in consultants and sonographers respectively) and government (44%, compared with 47% and 57% in consultants and sonographers respectively).

Univariate analysis by PPE (n=2233)

Table 4 focuses on common combinations of PPE used when performing an ultrasound scan.

Table 4: Breakdown of findings by combinations of personal protective equipment (PPE) (n = 2233)^.

	None	Gloves only	Gloves + surgical mask	Gloves + surgical mask + visor	Gloves + FFP3 mask	Gloves + FFP3 mask + visor	Gloves + FFP3 mask + visor + gown + surgical hat
**Number of respondents (n, %)**	76 (3%)	49 (2%)	497 (22%)	152 (7%)	95 (4%)	51 (2%)	90 (4%)
**Level of COVID-19 risk of working environment reported (n, %)**							
High risk of COVID-19 infection	50 (66%)	34 (69%)	394 (79%)	104 (68%)	60 (63%)	37 (73%)	73 (81%)
Moderate risk of COVID-19 infection	9 (12%)	3 (6%)	38 (8%)	16 (11%)	7 (7%)	6 (12%)	6 (7%)
Low risk of COVID-19 infection	17 (22%)	12 (24%)	65 (13%)	32 (21%)	28 (29%)	8 (16%)	11 (12%)
**Interaction w suspected/confirmed SARS-CoV-2 patients (n, %)**	22 (29%)	16 (33%)	226 (45%)	47 (31%)	31 (33%)	15 (29%)	34 (38%)
**Prevalence suspected/ confirmed SARS-CoV-2 based on symptoms +/- PCR testing (n)**	10 (13%)	14 (29%)	84 (17%)	17 (11%)	10 (11%)	2 (4%)	6 (7%)

For peer review only

**Number of respondents satisfied with local unit SARS-CoV-2 response (%)**	57 (75%)	33 (67%)	326 (66%)	112 (74%)	65 (68%)	38 (75%)	60 (67%)
**Mean % level of respondent satisfaction with government SARS-CoV-2 response (95% CI)**	63 (56-69)	68 (60-76)	50 (47-52)	48 (43-53)	42 (36-48)	40 (32-47)	47 (41-53)

^All possible combinations not included in this table, therefore denominator of table not 2233 (n = 1010). Main relevant PPE combinations described.

COVID-19 prevalence: 2.2% of respondents report using gloves in isolation. 29% of this group reported suspected or confirmed COVID-19.

The most commonly used combination of PPE when performing an ultrasound scan was gloves and a surgical mask (used by 22.3% of respondents). 45.4% (226/497) of participants using this combination of PPE reported interacting with confirmed SARS-Co-V-2 patients, with 16.9% of this group reporting suspected or confirmed COVID-19.

The use of gloves with an FFP3/N95 mask instead of a surgical mask when performing ultrasound was reported by 4.3% (without visor) and 2.3% (with visor) of HCP. 10.5% of those using gloves and FFP3/N95 mask reported suspected or confirmed COVID-19, compared with 3.9% of those who also used a visor.

4.0% of all HCPs reported using 'full' PPE, 81.1% of whom work in a COVID-19 high-risk setting. 'Full' PPE advised for use by clinicians when interacting with a suspected or confirmed SARS-CoV-2 patient includes gloves, gown, N95/FFP3 mask, a surgical hat and visor. 37.8% of those using 'full' PPE interacted at least once with suspected or confirmed SARS-CoV-2 patients. 6.7% of this group reported suspected or confirmed SARS-CoV-2.

82.4% HCPs reported patients wearing a mask during clinical interactions. This was within a high-risk area of work in 76.5% cases.

Anxiety, depression and support: HCPs that reported PPE shortages had higher rates of anxiety (45.4%) and depression (31.5%) compared to those without shortages (29.6% and 18.5% respectively) (Supplementary Material 9).

For peer review only

Lower satisfaction/support from their local unit ranged between 66-75% with mean level of government satisfaction ranging between 40-68% between the PPE subgroups.

For peer review only

DISCUSSION

Summary: We found that trainees and sonographers are generally more exposed to SARS-CoV-2, with sonographers seeing more patients who did not wear a mask and trainees working the most in higher risk areas. Trainees are thus more likely to interact with suspected or confirmed SARS-CoV-2 patients and have greater PPE concerns with poorer satisfaction and support. The most common PPE combination in use by HCPs were gloves and a surgical mask, with 18% of patients reported to not wear a mask. Prevalence of SARS-CoV-2 was lower amongst HCPs when visors, filtering face masks or 'full' PPE were used. Up to 1 in 5 respondents met the criteria for either moderate to severe anxiety or depression. Our findings suggest that suspected or confirmed SARS-CoV-2 prevalence amongst HCPs working with ultrasound in Obstetrics and Gynaecology was at least 13.0% at the time of the survey, with a higher prevalence in the UK

Strengths: The main strengths of our study are the relatively large sample size, the assessment of HCP responses internationally and the ability to compare responses between different HCPs. We translated the survey into four languages to include as many potential participants as possible. Furthermore, the response rate of ISUOG members was greater than non-ISUOG members, as expected given the target population. We compared responses between ISUOG and non-ISUOG members to ensure the data could be reliably grouped for the analysis.

Weaknesses: The key weakness of the study was the relatively low response rate and the method of sample selection that introduces possible selection bias when interpreting the results. As this is a descriptive study discussing early experience and wellbeing of HCP working with ultrasound in obstetrics and gynaecology during the evolving pandemic, it is important not to draw conclusions about causation. For example, we cannot link use of PPE to levels of anxiety and depression in this study. We combined suspected and confirmed SARS-CoV-2 as, at the time of writing, the majority of HCP did not have the same access to a swab or antibody test that is now available.

COVID-19 prevalence: Although international prevalence varies, at the time of writing confirmed global SARS-CoV-2 cases had surpassed 90 million. The suspected or confirmed SARS-CoV-2 prevalence of 13.0% in this international study is consistent with other

For peer review only

published reports on SARS-CoV-2 and HCP, where prevalence ranges from 1.6-22%(16-22). These studies have now been conducted across multiple specialities in many countries, with prevalence determined by PCR as well as antibody testing. One UK study within the field of Obstetrics and Gynaecology identified an HCP SARS-CoV-2 prevalence of 22%(18). Given that the advice for use of PPE is consistent within Obstetrics and Gynaecology(6), the higher prevalence may relate to respondents not using PPE, or not using it correctly. The prevalence suggested by our dataset may be associated with PPE supply concerns, the need to recycle or re-use PPE, a lack of guidance regarding when to use various levels of PPE, or due to many respondents experiencing high-risk interactions with patients, for example scanning in close proximity for prolonged periods of time.

PPE use and provision: More trainee doctors reported working in higher risk areas, as well as being more likely to interact with suspected or confirmed SARS-CoV-2 patients than consultants or sonographers. They also had higher levels of concern regarding PPE, re-using and recycling more PPE, with a poorer perception of unit and governmental satisfaction and support. It is well documented that HCP burnout relates to anxiety and depression and that burnout is particularly prevalent amongst trainees(23). Fears of infection and death during the pandemic may exacerbate this problem(24) and may also relate to inadequate support or protection. Sonographers also reported seeing more patients who did not wear a mask. The survey shows a worrying pattern where HCP's with the least agency (trainees and sonographers) were not only more likely to see more high-risk patients but were less likely to be protected. It is perhaps not surprising they expressed greater concerns than other colleagues. Consensus statements, national and international guidance have been published on advised levels of PPE in relation to SARS-CoV-2, with increasing requirements based on clinical risk. At the top end of the scale, disposable gloves, disposable fluid-repellent coverall/gown, filtering face piece respirator and eye/face protection should be used when performing aerosol generating procedures on possible or confirmed cases of SARS-CoV-2(6, 25, 26). 22.3% of study participants reported using gloves and a surgical mask, the combination recommended by the ISUOG consensus statement for HCPs performing ultrasound scans(6). Others reported using various combinations of PPE, some of which is recommended for high-risk, aerosol generating patient-HCP interactions. However, a proportion also reported not using PPE. This variation in PPE may be due to local policy,

For peer review only

dependent on wards or clinical areas where HCP duties extend beyond ultrasound, or a result of PPE supply issues. The use of 'full' PPE, or the addition of eye/face protection and/or filtering face piece respirators to gloves is associated with lower reports of suspected or confirmed SARS-CoV-2 when compared with gloves and surgical mask alone. This corroborates literature findings(19, 22).

Anxiety and depression: In our study, almost half the respondents reported some level of anxiety and depression, with 19.8% and 8.8% experiencing moderate to severe anxiety and depression respectively. Given the design of this study we must be careful not to relate this to COVID and be aware that there may be selection bias. There are aspects of the Obstetrics and Gynaecology environment which may encourage higher pre-existing levels of anxiety and depression amongst HCPs prior to the SARS-CoV-2 pandemic. The INDIGO study reports two thirds of Obstetrics and Gynecology trainees and consultants in the UK have been personally traumatised by work-related events unrelated to SARS-CoV-2, with 31% of them affected by post-traumatic stress disorder symptoms(27). 14.1% and 7.3% of paediatricians reported mild to severe anxiety and depression using HADS in a survey published prior to the pandemic(28), a specialty that reflects similar intensity.

This study provides insight into the experience and wellbeing of clinicians working in the field of ultrasound in Obstetrics and Gynaecology during the early phase of the SARS-CoV-2 pandemic. We hope the report highlights the importance of provision of PPE, the need for consistency in advice regarding appropriate PPE use, as well as the risk HCPs all take on a daily basis, particularly trainees and sonographers who are generally more exposed to SARS-CoV-2. As many patients may harbour infection with no or minimal symptoms, there is a real need to ensure HCPs are adequately protected at all times.

For peer review only

AUTHORS' CONTRIBUTIONS

TB, CK, HS and DT participated in the conception and design of the study. TB, CK and HS created the English version of the survey. JP, UM and CL translated survey to Spanish, French and Italian. JC performed the statistical analysis. TB, CK, HS, JC and DT interpreted the results. TB, CK and HS wrote the initial version of the manuscript. All authors critically revised the manuscript and approved the final version.

FUNDING STATEMENT

TB is supported by the NIHR Imperial Biomedical Research Centre, grant number IS-BRC-1215-20013. CK is supported by Imperial Health Charity, grant number RFPrD1920/116. DT is Fundamental Clinical Researcher of FWO (Research Foundation – Flanders). JC and DT are supported by Research Foundation – Flanders (FWO) grant number G0B4716N and Internal Funds KU Leuven grant number C24/15/037.

DATA AVAILABILITY STATEMENT

All data relevant to the study are included in the article or uploaded as supplementary information. No additional data available.

CONFLICT OF INTEREST STATEMENT

All authors have no conflicts of interest to declare.

For peer review only

REFERENCES

1. The L. COVID-19: protecting health-care workers. Lancet (London, England). 2020;395(10228):922.
2. Adams JG, Walls RM. Supporting the Health Care Workforce During the COVID-19 Global Epidemic. JAMA. 2020.
3. Abu-Rustum RS, Akolekar R, Sotiriadis A, Salomon LJ, Da Silva Costa F, Wu Q, et al. ISUOG Consensus Statement on organization of routine and specialist obstetric ultrasound services in the context of COVID-19. Ultrasound in Obstetrics & Gynecology.n/a(n/a).
4. Bourne T, Kyriacou C, Coomarasamy A, Al-Memar M, Leonardi M, Kirk E, et al. ISUOG Consensus Statement on rationalization of early-pregnancy care and provision of ultrasonography in context of SARS-CoV-2. Ultrasound in Obstetrics & Gynecology.n/a(n/a).
5. Wilson AN, Ravaldi C, Scoullar MJL, Vogel JP, Szabo RA, Fisher JRW, et al. Caring for the carers: Ensuring the provision of quality maternity care during a global pandemic. Women Birth. 2020.
6. Abramowicz JS, Basseal JM, Brezinka C, Dall'Asta A, Deng J, Harrison G, et al. ISUOG Safety Committee Position Statement on use of personal protective equipment and hazard mitigation in relation to SARS-CoV-2 for practitioners undertaking obstetric and gynecological ultrasound. Ultrasound Obstet Gynecol. 2020.
7. Lai J, Ma S, Wang Y, Cai Z, Hu J, Wei N, et al. Factors Associated With Mental Health Outcomes Among Health Care Workers Exposed to Coronavirus Disease 2019. JAMA Netw Open. 2020;3(3):e203976.
8. Lee AM, Wong JG, McAlonan GM, Cheung V, Cheung C, Sham PC, et al. Stress and psychological distress among SARS survivors 1 year after the outbreak. Can J Psychiatry. 2007;52(4):233-40.
9. Bansal P, Bingemann TA, Greenhawt M, Mosnaim G, Nanda A, Oppenheimer J, et al. Clinician Wellness During the COVID-19 Pandemic: Extraordinary Times and Unusual Challenges for the Allergist/Immunologist. J Allergy Clin Immunol Pract. 2020.
10. Albott CS, Wozniak JR, McGlinch BP, Wall MH, Gold BS, Vinogradov S. Battle Buddies: Rapid Deployment of a Psychological Resilience Intervention for Healthcare Workers during the COVID-19 Pandemic. Anesth Analg. 2020.
11. Shanafelt T, Ripp J, Trockel M. Understanding and Addressing Sources of Anxiety Among Health Care Professionals During the COVID-19 Pandemic. JAMA. 2020.
12. Dewey C, Hingle S, Goelz E, Linzer M. Supporting Clinicians During the COVID-19 Pandemic. Ann Intern Med. 2020.
13. WHO. WHO Coronavirus Disease (COVID-19) Dashboard: Word Helath Organization; 2020 [Available from: https://covid19.who.int/.
14. Yang J, Zheng Y, Gou X, Pu K, Chen Z, Guo Q, et al. Prevalence of comorbidities and its effects in patients infected with SARS-CoV-2: a systematic review and meta-analysis. Int J Infect Dis. 2020;94:91-5.
15. Zigmond AS, Snaith RP. The hospital anxiety and depression scale. Acta Psychiatr Scand. 1983;67(6):361-70.
16. Kluytmans-van den Bergh MFQ, Buiting AGM, Pas SD, Bentvelsen RG, van den Bijllaardt W, van Oudheusden AJG, et al. Prevalence and Clinical Presentation of Health Care Workers With Symptoms of Coronavirus Disease 2019 in 2 Dutch Hospitals During an Early Phase of the Pandemic. JAMA Netw Open. 2020;3(5):e209673.

For peer review only

17. Sikkema RS, Pas SD, Nieuwenhuijse DF, O'Toole Á, Verweij J, van der Linden A, et al. COVID-19 in health-care workers in three hospitals in the south of the Netherlands: a cross-sectional study. Lancet Infect Dis. 2020.
18. Khalil A, Hill R, Wright A, Ladhani S, O'Brien P. SARS-CoV-2-specific antibody detection in healthcare workers in a UK maternity Hospital: Correlation with SARS-CoV-2 RT-PCR results. Clin Infect Dis. 2020.
19. Korth J, Wilde B, Dolff S, Anastasiou OE, Krawczyk A, Jahn M, et al. SARS-CoV-2-specific antibody detection in healthcare workers in Germany with direct contact to COVID-19 patients. J Clin Virol. 2020;128:104437.
20. Rivett L, Sridhar S, Sparkes D, Routledge M, Jones NK, Forrest S, et al. Screening of healthcare workers for SARS-CoV-2 highlights the role of asymptomatic carriage in COVID-19 transmission. Elife. 2020;9.
21. Chen Y, Tong X, Wang J, Huang W, Yin S, Huang R, et al. High SARS-CoV-2 antibody prevalence among healthcare workers exposed to COVID-19 patients. J Infect. 2020;81(3):420-6.
22. Chatterjee P, Anand T, Singh KJ, Rasaily R, Singh R, Das S, et al. Healthcare workers & SARS-CoV-2 infection in India: A case-control investigation in the time of COVID-19. Indian J Med Res. 2020;151(5):459-67.
23. Bourne T, Shah H, Falconieri N, Timmerman D, Lees C, Wright A, et al. Burnout, well-being and defensive medical practice among obstetricians and gynaecologists in the UK: cross-sectional survey study. BMJ Open. 2019;9(11):e030968.
24. Hu D, Kong Y, Li W, Han Q, Zhang X, Zhu LX, et al. Frontline nurses' burnout, anxiety, depression, and fear statuses and their associated factors during the COVID-19 outbreak in Wuhan, China: A large-scale cross-sectional study. EClinicalMedicine. 2020:100424.
25. PHE. COVID-19 personal protective equipment (PPE). https://www.gov.uk/government/publications/wuhan-novel-coronavirus-infection-prevention-and-control/covid-19-personal-protective-equipment-ppe. 2020.
26. WHO. Rational use of personal protective equipment for coronavirus disease (COVID-19) and considerations during severe shortages. https://www.who.int/publications/i/item/rational-use-of-personal-protective-equipment-for-coronavirus-disease-(covid-19)-and-considerations-during-severe-shortages. 2020.
27. Slade P, Balling K, Sheen K, Goodfellow L, Rymer J, Spiby H, et al. Work-related post-traumatic stress symptoms in obstetricians and gynaecologists: findings from INDIGO, a mixed-methods study with a cross-sectional survey and in-depth interviews. BJOG. 2020;127(5):600-8.
28. van Steijn ME, Scheepstra KWF, Yasar G, Olff M, de Vries MC, van Pampus MG. Occupational well-being in pediatricians-a survey about work-related posttraumatic stress, depression, and anxiety. Eur J Pediatr. 2019;178(5):681-93.

For peer review only

TABLES AND SUPPLEMENTARY MATERIAL

Table 1: Demographics of total survey participation (n = 3237).
Table 2: Breakdown of findings by country (n = 2233).
Table 3: Breakdown of findings by healthcare professional (HCP) (n = 2233).
Table 4: Breakdown of findings by combinations of personal protective equipment (PPE) (n = 2233).

Supplement 1: The ISUOG SARS-CoV-2 Questionnaire for healthcare professionals working with ultrasound in Obstetrics and Gynecology.
Supplement 2: Original study protocol.
Supplement 3: Breakdown of HCP roles for analysis.
Supplement 4: Breakdown of risk by area of work for analysis.
Supplement 5: Flowchart summarizing survey participants, differentiating ISUOG members and non-ISUOG members (n = 3287).
Supplement 6: Demographics of survey participation, differentiated based on ISUOG membership (n = 3237).
Supplement 7: Comparison of comorbidity in those with (n = 290) and without suspected or confirmed SARS-CoV-2 (n = 290) (Total n = 2237).
Supplement 8: Comparison of HADS outcome score in those with (n = 162) and without suspected or confirmed SARS-CoV-2 (n = 896) (Total n = 1058).
Supplement 9: Comparison of HADS outcome score in those with (n = 788) and without PPE shortages (n = 270) (Total n = 1058).

For peer review only

SUPPLEMENTARY MATERIAL

The experiences and wellbeing of healthcare professionals working in the field of ultrasound in Obstetrics and Gynecology as the SARS-CoV-2 pandemic was evolving: a cross-sectional survey study

Authors

Tom Bourne[1,2], Chris Kyriacou[1], Harsha Shah[1], Jolien Ceusters[3], Jessica Preisler[4,5], Ulrike Metzger[6], Chiara Landolfo[7], Christoph Lees[8], Dirk Timmerman[2,9]

Affiliations

1. Early pregnancy and Acute Gynaecology Unit, Department of Obstetrics and Gynaecology, Queen Charlotte's and Chelsea Hospital, Imperial College, London, UK.

2. Department of Development and Regeneration, KU Leuven, Leuven, Belgium.

3. Department of Oncology, Laboratory of Tumour Immunology and Immunotherapy, KU Leuven, Leuven, Belgium.

4. Facultad de Medicina, Clínica Alemana – Universidad del Desarrollo, Santiago, Chile

5. Hospital Clínico de la Universidad de Chile, Santiago, Chile.

6. Centre d'Échographie de l'Odéon, Paris, France.

7. Dipartimento Scienze della Salute della Donna, del Bambino e di Sanità Pubblica, Fondazione Policlinico Universitario Agostino Gemelli, IRCCS, Rome, Italy.

8. Centre for Fetal Care, Department of Obstetrics and Gynaecology, Queen Charlotte's and Chelsea Hospital, Imperial College, London, UK.

9. Department of Obstetrics and Gynaecology, University Hospitals Leuven, Leuven, Belgium.

Correspondence to:

Professor Tom Bourne

Queen Charlotte's and Chelsea Hospital, Imperial College London

Du Cane Road, London, W12 0HS, UK

t.bourne@imperial.ac.uk

For peer review only

Supplement 1: The ISUOG SARS-CoV-2 Questionnaire for healthcare professionals working with ultrasound in Obstetrics and Gynecology.

Informed consent

This questionnaire is fully anonymous - answers cannot be traced back to participants. The anonymised data will be collected for analysis in order to provide a report and publication at a later date.

1. **Please click 'yes' to participate in this survey. Otherwise, click 'no': (please choose one option)**

Yes
No

Introduction

2. **What title best describes your job: (please choose one option)**

Sonographer – Obstetrics
Sonographer – Gynecology
Sonographer – Obstetrics and Gynecology
Obstetrician
Gynecologist
Obstetrician and Gynecologist
Radiologist
Doctor in training – Obstetrics
Doctor in training – Gynecology
Doctor in training – Obstetrics and Gynecology
Doctor in training – Radiologist
Doctor in research – Obstetrics
Doctor in research – Gynecology
Doctor in research – Obstetrics and Gynecology
Doctor in research – Radiology
Nurse – Obstetrics
Nurse – Gynecology
Nurse – Obstetrics and Gynecology
Nurse – Early pregnancy only
Midwife – Obstetrics
Midwife – Early pregnancy only
Retired

3. **In what country do you practice? (please state below)**

Afghanistan
Albania
Algeria
Andorra
Angola
Antigua and Barbuda

For peer review only

Argentina
Armenia
Australia
Austria
Azerbaijan
The Bahamas
Bahrain
Bangladesh
Barbados
Belarus
Belgium
Belize
Benin
Bhutan
Bolivia
Bosnia and Herzegovina
Botswana
Brazil
Brunei
Bulgaria
Burkina Faso
Burundi
Cabo Verde
Cambodia
Cameroon
Canada
Central African Republic
Chad
Chile
China
Colombia
Comoros
Congo, Democratic Republic of the
Congo, Republic of the
Costa Rica
Côte d'Ivoire
Croatia
Cuba
Cyprus
Czech Republic
Denmark
Djibouti
Dominica
Dominican Republic
East Timor (Timor-Leste)
Ecuador
Egypt

For peer review only

El Salvador
Equatorial Guinea
Eritrea
Estonia
Eswatini
Ethiopia
Fiji
Finland
France
Gabon
The Gambia
Georgia
Germany
Ghana
Greece
Grenada
Guatemala
Guinea
Guinea-Bissau
Guyana
Haiti
Honduras
Hungary
Iceland
India
Indonesia
Iran
Iraq
Ireland
Israel
Italy
Jamaica
Japan
Jordan
Kazakhstan
Kenya
Kiribati
Korea, North
Korea, South
Kosovo
Kuwait
Kyrgyzstan
Laos
Latvia
Lebanon
Lesotho
Liberia

For peer review only

Libya
Liechtenstein
Lithuania
Luxembourg
Madagascar
Malawi
Malaysia
Maldives
Mali
Malta
Marshall Islands
Mauritania
Mauritius
Mexico
Micronesia, Federated States of
Moldova
Monaco
Mongolia
Montenegro
Morocco
Mozambique
Myanmar (Burma)
Namibia
Nauru
Nepal
Netherlands
New Zealand
Nicaragua
Niger
Nigeria
North Macedonia
Norway
Oman
Pakistan
Palau
Panama
Papua New Guinea
Paraguay
Peru
Philippines
Poland
Portugal
Qatar
Romania
Russia
Rwanda
Saint Kitts and Nevis

For peer review only

1 Saint Lucia
2 Saint Vincent and the Grenadines
3 Samoa
4 San Marino
5 Sao Tome and Principe
6 Saudi Arabia
7 Senegal
8 Serbia
9 Seychelles
10 Sierra Leone
11 Singapore
12 Slovakia
13 Slovenia
14 Solomon Islands
15 Somalia
16 South Africa
17 Spain
18 Sri Lanka
19 Sudan
20 Sudan, South
21 Suriname
22 Sweden
23 Switzerland
24 Syria
25 Taiwan
26 Tajikistan
27 Tanzania
28 Thailand
29 Togo
30 Tonga
31 Trinidad and Tobago
32 Tunisia
33 Turkey
34 Turkmenistan
35 Tuvalu
36 Uganda
37 Ukraine
38 United Arab Emirates
39 United Kingdom
40 United States
41 Uruguay
42 Uzbekistan
43 Vanuatu
44 Vatican City
45 Venezuela
46 Vietnam
47 Yemen

For peer review only

Zambia
Zimbabwe

4. Are you an ISUOG member? (please choose one option)

Yes
No

5. If you answered no, which society or professional body did you receive this survey from?

Open box answer

<u>Demographics</u>

6. How old are you? (please state number of years)

Numbers offered

7. What sex were you assigned at birth? (please choose one option)

Male
Female
Intersex
Prefer not to say

8. What gender do you most identify with? (please choose one option)

Male
Female
Prefer not to say
Other (please specify)

<u>Underlying medical conditions</u>

9. Do you have any underlying medical conditions that place you at increased risk during the SARS-CoV-2 pandemic? (please tick all that apply)

None
Heart disease
Hypertension
Diabetes
Asthma
Other lung disease
Kidney disease
Cancer

For peer review only

Medication

10. Are you taking any of the following medications? (please tick all that apply)

None
Immunosuppressant medication - steroids
Immunosuppressant medication - methotrexate
Immunosuppressant medication - biological agents
Aspirin
Indomethacin
Any other non-steroidal anti-inflammatory drugs (NSAIDS) e.g. ibuprofen, diclofenac, naproxen
Angiotensin converting enzyme inhibitors (ACEi) blood pressure medication - any ending in -pril (e.g. enalapril)
Any other blood pressure medication e.g. angiotensin receptor blockers (ARB), alpha blockers, alpha-2 receptor agonists, beta blockers, combined alpha and beta blockers, calcium channel blockers (CCB), diuretics, other

Smoking and BMI

11. Do you smoke? (please choose one option)

Yes
Previously – now stopped
No

12. What was your last measured weight? (please choose one option)

In kilograms (kg) – numbers offered

13. What is your height? (please choose one option)

In centimetres (cm) – numbers offered

Role, shift pattern, PPE

14. How has your role and shift pattern been affected by the SARS-CoV-2 pandemic? (please choose one option)

Unable to work for the duration of the pandemic
Reduced duties with no patient contact
Reduced duties with reduced patient contact
Reduced duties with similar patient contact
Reduced duties with increased patient contact
Similar duties with no patient contact
Similar duties with reduced patient contact
Similar duties with similar patient contact
Similar duties with increased patient contact
Increased duties with no patient contact
Increased duties with reduced patient contact
Increased duties with similar patient contact

For peer review only

Increased duties with increased patient contact

15. Where are you working during the SARS-CoV-2 pandemic? (please tick all that apply)

Obstetrics – Labor ward
Obstetrics – Birth center
Obstetrics – Antenatal ward
Obstetrics – Postnatal ward
Obstetrics – Maternity triage
Obstetrics – Antenatal clinic
Obstetrics – Maternity helplines
Obstetrics – Community clinics
Obstetrics – Ultrasound
Gynecology – Acute gynecology
Gynecology – Benign gynecology
Gynecology – Oncology
Gynecology – Ultrasound
Early pregnancy unit – Ultrasound and assessment
Radiology – Ultrasound
Radiology – non-ultrasound
Obstetrics/Gynecology/Radiology – From home

16. Do you interact with patients confirmed or suspected to have SARS-CoV-2? (please choose one option)

Yes – every day
Yes – every week
Yes – every so often
I don't know
Not yet
No

17. Have you been using personal protective equipment (PPE) since the SARS-CoV-2 outbreak? (please choose one option)

No
Not yet
Yes – for aerosol generating procedures only
Yes – for suspected/positive patients only
Yes – for all patients

18. When you perform an ultrasound scan please state what PPE you use: (please tick all that apply)

None
Gloves
Surgical mask
N95/FFP3 mask
Gown
Visor

For peer review only

Surgical hat

19. Have there been any concerns regarding PPE provision or shortages since the SARS-CoV-2 outbreak? (note – Full PPE includes a N95/FFP3 mask, visor, surgical hat, gown and gloves) (please tick all that apply)

No issues with provision
Yes – supply issues with gloves
Yes – supply issues with surgical masks
Yes – supply issues with N95/FFP3 masks
Yes – supply issues with gowns
Yes – supply issues with visors
Yes – supply issues with surgical hats
Yes – supply issues with providing patients with a surgical mask

20. Have you been asked to re-use or recycle any PPE? (please tick all that apply option)

No
Yes - Gloves
Yes - Surgical mask
Yes - N95/FFP3 mask
Yes - Gown
Yes - Visor
Yes - Surgical hat

21. Do you ask patients to wear a surgical mask? (please choose one option)

Yes
No

<u>**SARS-CoV-2 diagnosis**</u>

22. Have you had or do you have SARS-CoV-2 based on clinical symptoms and/or a SARS-CoV-2 swab (PCR) diagnosis? (please choose one option)

No – no test or symptoms suggestive of SARS-CoV-2
Yes – suspected based on symptoms but have not had PCR swab testing
Yes – suspected based on symptoms, had PCR testing, which was negative
Yes – confirmed on PCR swab testing but have had no symptoms
Yes – suspected based on symptoms and confirmed on PCR swab testing

23. With regards to having a SARS-CoV-2 swab test, please choose one option:

Not been offered
Offered as experiencing symptoms
Offered as in contact with confirmed or probable SARS-CoV-2 patients
Offered as living with someone who has probable or confirmed SARS-CoV-2
Not applicable

For peer review only

24. What symptoms have you experienced or are you experiencing? (please tick all that apply)

None
High fever
Persistent dry cough
Productive cough
Shortness of breath/Difficulty breathing
Fatigue
Headache
Loss of sense of smell and/or taste
Sore throat
Chest pain
Diarrhoea
Myalgia
Loss of appetite
Hoarse voice
Abdominal pain

Personal SARS-CoV-2 pathway

25. At what point were you offered a SARS-CoV-2 swab? (please state number of days into your illness with day 1 being the day you started to have symptoms. If you have not had symptoms – please choose 'not had symptoms'. If you were not offered a test, please choose 'not offered a test')

Not had symptoms
Not offered a test
Not applicable
Numbers offered

26. How severe was/is your infection? (please choose one option)

Mild COVID-19 (T< 37.8 oC, tiredness, muscle aches, headache, no breathlessness, normal appetite)
Moderate COVID-19 (T> 37.8 oC, troublesome cough, breathless on exercise, tired but no chest or abdominal pain)
Severe COVID-19 (T> 37.8 oC , noticeably breathless even when sitting still, unable to complete sentences when talking, chest or abdominal pain, reduced appetite)
Critical COVID-19 (development of severe acute respiratory syndrome in need of ventilator or other respiratory support)
Not applicable

27. How long were you or have you been symptomatic for? (please state number of days. If you have not had symptoms – please choose 'not had symptoms')

Not had symptoms
Numbers offered

For peer review only

28. How long did you stay at home before going back to work, or if current infection, how long have you been at home for? (please state number of days. If you have not had symptoms – please choose 'not had symptoms')

Not had symptoms
Numbers offered

29. Did you or do you feel under pressure from your employers to return to work before making a full recovery? (please choose one option)

Yes
No
Not applicable

30. If you have been unwell with presumed or confirmed SARS-CoV-2, how long were you advised to stay at home by your employers before going back to work? (please state number of days. If you have not had symptoms – please choose 'not had symptoms')

Not had symptoms
Numbers offered

31. Have you had a SARS-CoV-2 antibody test? (please choose one option)

No – no test or symptoms suggestive of SARS-CoV-2
No – not offered
No – offered and declined
Yes – negative
Yes – positive

<u>Household</u>

32. Have you needed to, or are you currently self-isolating due to a member of your household having suspected or confirmed SARS-CoV-2? (please choose one option)

Yes
No

33. How long did you need or have you needed to self-isolate due to a member of your household having suspected or confirmed SARS-CoV-2? (please state number of days. If your household has been well - please choose 'household has been well')

Household has been well
Numbers offered

For peer review only

Colleagues

34. How many colleagues do you work with in your unit? (please state number of each group. Select 'unsure' if you do not know)

Sonographers
Consultants
Doctors in training
Doctors in research
Nurses
Midwifes
Unsure
Numbers offered

35. To your knowledge, how many colleagues are you aware of in your unit that have had a presumed diagnosis of SARS-CoV-2 based on symptoms or a positive swab? (please state number of each group. Select 'unsure' if you have no information)

Sonographers
Consultants
Doctors in training
Doctors in research
Nurses
Midwifes
Unsure
Numbers offered

36. To your knowledge, how many colleagues have needed to self-isolate or are self-isolating due to a member of their household having suspected or confirmed SARS-CoV-2? (please state number of each group. Select 'unsure' if you have no information)

Sonographers
Consultants
Doctors in training
Doctors in research
Nurses
Midwifes
Unsure
Numbers offered

37. What is the longest period of time any of your colleagues have taken off in isolation because they or a household member have suspected or confirmed SARS-CoV-2? (please state number of days. Select 'unsure' if you have no information)

Unsure
Numbers offered

For peer review only

Support

38. Have you felt or do you feel adequately supported by your unit during the SARS-CoV-2 pandemic? (please choose one option)

Yes
No

39. How do you rate the response and support that was provided by your country's Ministry of Health or Public Health officials? (please answer using the scale below)

0 - Extremely poor
50 - Adequate
100 - Excellent

40. Please tell us, if you wish, your views regarding how well clinician safety has been protected by your employers (please state in the text box below. If you choose not to answer, please write 'not applicable')

Open box answer

Hospital Anxiety and Depression Scale (HADS)

41. Thank you for filling out this questionnaire.

Optional: Hospital Anxiety and Depression Scale (HADS). If you are willing to proceed, please click yes. Otherwise, click no to end the survey.

Yes
No

Hospital Anxiety and Depression Scale (HADS)

42. Choose the reply that is closest to how you have been feeling in the past week. Don't take too long over your replies: your immediate is best.

I feel tense or 'wound up':

Most of the time
A lot of the time
From time to time, occasionally
Not at all

43. Choose the reply that is closest to how you have been feeling in the past week. Don't take too long over your replies: your immediate is best.

I still enjoy the things I used to enjoy:

Definitely as much
Not quite as much

For peer review only

Only a little
Hardly at all

44. Choose the reply that is closest to how you have been feeling in the past week. Don't take too long over your replies: your immediate is best.

I get a sort of frightened feeling as if something awful is about to happen:
Very definitely and quite badly
Yes, but not too badly
A little, but it doesn't worry me
Not at all

45. Choose the reply that is closest to how you have been feeling in the past week. Don't take too long over your replies: your immediate is best.

I can laugh and see the funny side of things:
As much as I always could
Not quite so much now
Definitely not so much now
Not at all

46. Choose the reply that is closest to how you have been feeling in the past week. Don't take too long over your replies: your immediate is best.

Worrying thoughts go through my mind:
A great deal of the time
A lot of the time
From time to time, but not too often
Only occasionally

47. Choose the reply that is closest to how you have been feeling in the past week. Don't take too long over your replies: your immediate is best.

I feel cheerful:
Not at all
Not often
Sometimes
Most of the time

48. Choose the reply that is closest to how you have been feeling in the past week. Don't take too long over your replies: your immediate is best.

I can sit at ease and feel relaxed:
Definitely
Usually
Not often
Not at all

For peer review only

49. Choose the reply that is closest to how you have been feeling in the past week. Don’t take too long over your replies: your immediate is best.

I feel as if I am slowed down:
Nearly all the time
Very often
Sometimes
Not at all

50. Choose the reply that is closest to how you have been feeling in the past week. Don’t take too long over your replies: your immediate is best.

I get a sort of frightened feeling like 'butterflies' in the stomach:
Not at all
Occasionally
Quite often
Very often

51. Choose the reply that is closest to how you have been feeling in the past week. Don’t take too long over your replies: your immediate is best.

I have lost interest in my appearance:
Definitely
I don't take as much care as I should
I may not take quite as much care
I take just as much care as ever

52. Choose the reply that is closest to how you have been feeling in the past week. Don’t take too long over your replies: your immediate is best.

I feel restless as I have to be on the move:
Very much indeed
Quite a lot
Not very much
Not at all

53. Choose the reply that is closest to how you have been feeling in the past week. Don’t take too long over your replies: your immediate is best.

I look forward with enjoyment to things:
As much as I ever did
Rather less than I used to
Definitely less than I used to
Hardly at all

For peer review only

54. Choose the reply that is closest to how you have been feeling in the past week. Don't take too long over your replies: your immediate is best.

I get sudden feelings of panic:
Very often indeed
Quite often
Not very often
Not at all

55. Choose the reply that is closest to how you have been feeling in the past week. Don't take too long over your replies: your immediate is best.

I can enjoy a good book or radio or TV program:
Often
Sometimes
Not often
Very seldom

56. Thank you for filling out this questionnaire. Please complete last question to end. Please tell us, if you wish, how you have felt emotionally during the SARS-CoV-2 pandemic? (please state in the text box below. If you choose not to answer, please write 'not applicable')
Open box answer

For peer review only

Supplement 2: Original study protocol

Title: An international survey of the impact of COVID19 on healthcare professionals carrying out ultrasonography, service provision and mental state in Obstetrics and Gynecology

Date: 19th April 2020

Main Sponsor: UZ Leuven Belgium
Carried out in collaboration with: International Society of Ultrasound in Obstetrics and Gynecology (ISUOG)
Version number: 1
Principal Investigators**:** Professor Tom Bourne and Professor Dirk Timmerman
Co-Investigators: Dr Christopher Kyriacou, Dr Harsha Shah
Protocol to be assessed by: EC Onderzoek UZ Leuven, Belgium.

Design: Prospective cohort observational survey study.

Aim: To assess the services and psychological impact of the COVID-19 pandemic to healthcare professionals carrying out ultrasonography within the Obstetrics and Gynecology specialty.

Hypothesis:

1. Service provision to support healthcare professionals during the COVID-19 pandemic varies considerably in every unit globally;
2. Healthcare professionals will have experienced COVID-19 first-hand;
3. The COVID-19 pandemic has caused significant psychological distress to healthcare professionals which can be assessed using a validated psychological tool.

Outcome measures:

1. A questionnaire to healthcare professionals detailing medical history, role, area of work, personal protective equipment (PPE), COVID-19 patient contact and personal COVID-19 experience will allow data to be collected in relation to the physical impact of the COVID-19 pandemic;
2. Use of the validated Hospital Anxiety and Depression Scale (HADS) will allow psychological morbidity of healthcare professionals to be assessed.

Eligibility: Members of ISUOG that fill in the questions via an online survey platform. The questionnaire will in English, Spanish, Greek, Hindu, Mandarin and Cantonese.

Duration: The survey will be conducted over a period of two weeks in order to allow time for the questions to be answered, two reminder emails will be sent – one at a week before closure and one 24 hours before closure

Consent: Consent will be implied when the questionnaire is filled in and there will be a specific question asking for consent to take part in the survey, however all information is anonymized and cannot be traced to an individual. No data will be requested that is in breach of General Data Protection Regulation (GDPR).

For peer review only

Background: The current coronavirus (SARS-CoV-2) pandemic carries a number of new challenges to local, national and international healthcare agencies in order to protect patients and healthcare professionals (physicians, sonographers, allied healthcare professionals). Guidance varies from nation to nation with issues arising from redeployment of clinicians, prioritization of particular services and provision of PPE. This is important as those carrying out ultrasound scans are in close proximity to patients and are at high risk of SARS-CoV-2 infection[1].
Anxiety and depression are recognized complications for healthcare professionals working in a high-stress environment, particularly one that now encompasses a novel disease for which we have yet to fully understand[2].

Methods: This is a prospective cohort study and will recruit via the dissemination of a survey online through the ISUOG mailing list. Consent will be presumed when the form is filled in and submitted. This will allow healthcare professionals globally to answer. The questionnaire has been devised to assess the service provision in units around the world as well as question personal COVID-19 experience. Following on from this will be an optional HADS. The HADS is a 14-part questionnaire with 7 questions exploring symptoms of anxiety and depression, respectively. Each question is answered with a scale of 0-3 to represent severity of symptoms, with an independent maximum score of 21 for depression and anxiety. A score $\geq$11 is deemed moderate, whilst $\geq$16 represents severe symptoms[3].

Statistics and data analysis: Sample size will be dependent on the number of online responders. We hope that there will be significant uptake from a number of countries globally. The recruitment will be online so no breach to social distancing rules will apply. To compare differences in binary variables we will use the likelihood ratio, chi-squared or Fisher's exact test and will consider a p value <0.05 as statistically significant. Ninety-five per cent confidence intervals for rates of psychological morbidity will be created using the Wilson method.

Dissemination: Following the collection and analysis of the data obtained from the questionnaire, we aim to present the findings to the ISUOG membership and publish in peer-reviewed journals – preferably Ultrasound in Obstetrics and Gynecology. The need to understand the experience of the pandemic by frontline workers is extremely important, not only to support them now, but to prepare for any future international healthcare emergency.

References

1. Abramowicz JS, Basseal JM, Brezinka C, Dall'Asta A, Deng J, Harrison G, Marsal K, Lee JCS, Lim A, Miloro P, Poon LC, Salvesen KJ, Sande R, ter Haar G, Westerway SC, Xie MX, Lees C. ISUOG Safety Committee Position Statement on use of personal protective equipment and hazard mitigation in relation to SARS-CoV-2 for practitioners undertaking obstetric and gynecological ultrasound. *ISUOG*. 2020 March 28. Available from: https://www.isuog.org/uploads/assets/b8dde768-08a2-424c-a4615551637515e9/ISUOG-Safety-Committee-statement-on-COVID19-and-PPE.pdf
2. Neto MLR, Almeida HG, Esmeraldo JD, Nobre CB, Pinheiro WR, de Oliveira CRT, Sousa IDC, Lima OMML, Lima NNR, Moreira MM, Lima CKT, Junior JG, da Silva CGL.

For peer review only

When health professionals look death in the eye: the mental health of professionals who deal daily with the 2019 coronavirus outbreak. *Psychiatry Res*. 2020 April 13;288:112972. doi:10.1016/j.psychres.2020.112972.

3. Zigmond AS, Snaith RP. The hospital anxiety and depression scale. Acta psychiatrica scandinavica. 1983 Jun;67(6):361-70.

For peer review only

Supplement 3: Breakdown of HCP roles for analysis.

Job title	
Doctor in research – Gynecology	Split up based on age: trainees/residents and consultants/attending
Doctor in research – Obstetrics	Split up based on age: trainees/residents and consultants/attending
Doctor in research – Obstetrics and Gynecology	Split up based on age: trainees/residents and consultants/attending
Doctor in research – Radiology/Sonologist	Split up based on age: trainees/residents and consultants/attending
Doctor in training – Gynecology	Split up based on age: trainees/residents and consultants/attending
Doctor in training – Obstetrics	Split up based on age: trainees/residents and consultants/attending
Doctor in training – Obstetrics and Gynecology	Split up based on age: trainees/residents and consultants/attending
Doctor in training – Radiologist/Sonologist	Split up based on age: trainees/residents and consultants/attending
Gynecologist	Split up based on age: trainees/residents and consultants/attending
Midwife – Early pregnancy only	Sonographer
Midwife – Obstetrics	Sonographer
Nurse – Early pregnancy only	Sonographer
Nurse – Gynecology	Sonographer
Nurse – Obstetrics	Sonographer
Nurse – Obstetrics and Gynecology	Sonographer
Obstetrician	Split up based on age: trainees/residents and consultants/attending
Obstetrician and Gynecologist	Split up based on age: trainees/residents and consultants/attending
Radiologist/Sonologist	Split up based on age: trainees/residents and consultants/attending
Retired	Exclude from analyses
Sonographer – Gynecology	Sonographer
Sonographer – Obstetrics	Sonographer
Sonographer – Obstetrics and Gynecology	Sonographer

For peer review only

1 **Supplement 4: Breakdown of risk by area of work for analysis.**

Areas of work	Risk of COVID-19 infection
Early pregnancy unit - Ultrasound and assessment	High risk
Gynecology - Acute Gynecology	High risk
Gynecology - Benign Gynecology	Low risk
Gynecology – Oncology	Low risk
Gynecology - Ultrasound	High risk
Obstetrics/Gynecology/Radiology - From home	Low risk
Obstetrics - Antenatal clinic	Moderate risk
Obstetrics - Antenatal ward	Moderate risk
Obstetrics - Birth center/Birthing center	High risk
Obstetrics - Community clinics	Moderate risk
Obstetrics - Labor ward	High risk
Obstetrics - Maternity helplines	Low risk
Obstetrics - Maternity triage/Hospital obstetric triage	High risk
Obstetrics - Postnatal ward	Moderate risk
Obstetrics – Ultrasound	High risk
Radiology - non-Ultrasound	Moderate risk
Radiology - Ultrasound	High risk

2
3

For peer review only

Supplement 5: Flowchart summarizing survey participants, differentiating ISUOG members and non-ISUOG members (n = 3287).

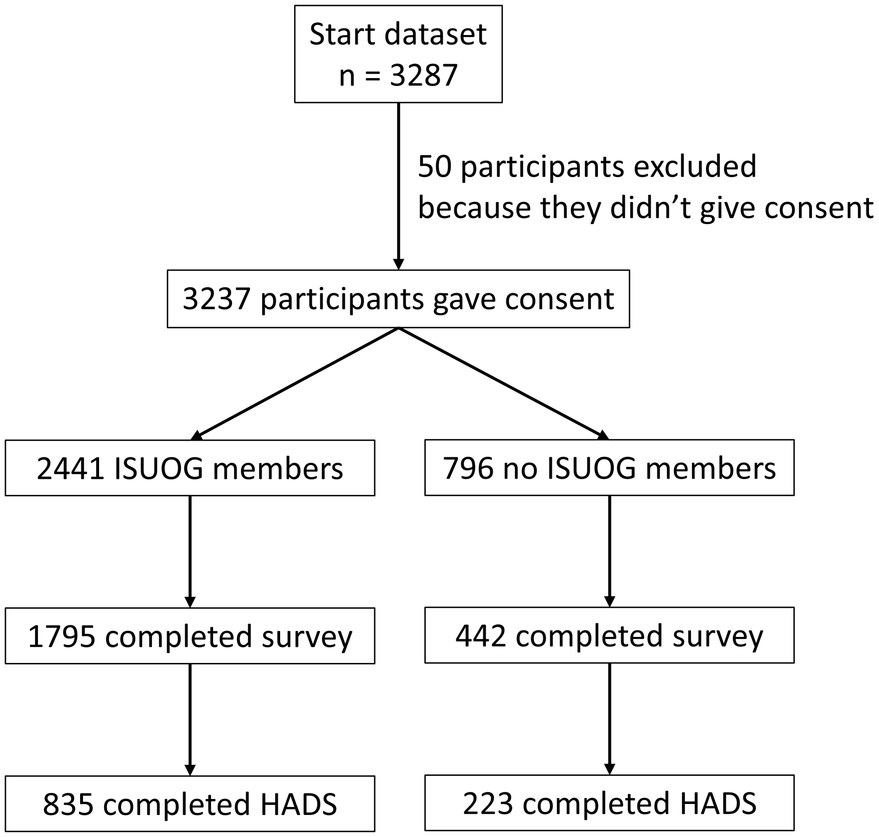

Response rate:
ISUOG members - 23.1% (2441/10589)
Non-ISUOG members - 3.2% (796/24920).

view only

1 **Supplement 6:** Demographics of survey participation, differentiated based on ISUOG
2 membership (n = 3237).

	ISUOG members (n = 2441)	Others (n = 796)
**Total ISUOG mailing list (n)**	10589	24920
**Response of total ISUOG mailing list (n, %)**	2441 (23%)	796 (3%)
Countries (n)	120	78
Number of respondents in English (n)	1603	357
Number of respondents in Spanish (n)	564	317
Number of respondents in Italian (n)	195	72
Number of respondents in French (n)	79	50
**Completed survey**	**1795**	**442**
**Countries (n)**	110	65
**Age (mean, range)**	47.2 (18-82)	47.7 (25-78)
**Gender (n, %)**		
Female	1176 (66%)	298 (68%)
Male	613 (34%)	142 (32%)
Intersex	1 (0%)	0 (0%)
Prefer not to say	3 (0%)	0 (0%)
**Prevalence suspected or confirmed SARS-CoV-2 based on symptoms +/- PCR testing (%)**	230 (13%)	60 (14%)
**Is PPE used by respondents (n, %)**		
Not yet	217 (12%)	62 (14%)
Yes - for aerosol generating procedures only	110 (6%)	23 (5%)
Yes - for all patients	1142 (64%)	250 (57%)
Yes - for suspected/positive patients only	324 (18%)	105 (24%)
**What PPE is used by respondents (n, %)**		
None	59 (3%)	17 (4%)
Gloves only	38 (2%)	11 (3%)
Gloves + surgical mask	393 (22%)	104 (24%)
Gloves + surgical mask + visor	116 (6%)	36 (8%)
Gloves + FFP3 mask	83 (5%)	12 (3%)
Gloves + FFP3 mask + visor	39 (2%)	12 (3%)
Gloves + FFP3 mask + visor + gown + surgical hat	70 (4%)	20 (5%)
Patient wearing mask	1482 (83%)	359 (82%)
**Concerns regarding a lack of PPE (n, %)**		
No	475 (26%)	104 (24%)
Gloves	223 (12%)	55 (13%)
Gown	499 (28%)	130 (30%)
N95/FFP3 mask	1014 (57%)	265 (60%)
Surgical hat	187 (10%)	46 (10%)
Surgical mask	657 (37%)	163 (37%)

For peer review only

Visor	458 (26%)	124 (28%)
Surgical mask (patient)	479 (27%)	130 (30%)
**Type of PPE that has been re-used or recycled (n, %)**		
No	690 (38%)	175 (40%)
Gloves	27 (2%)	7 (2%)
Gown	229 (13%)	58 (13%)
N95/FFP3 mask	722 (40%)	164 (37%)
Surgical hat	74 (4%)	18 (4%)
Surgical mask	435 (24%)	130 (30%)
Visor	493 (27%)	128 (29%)
**Anxiety (mean, 95% CI)***	7 (6.7-7.3)	6.6 (6.1-7.1)
0-7 (none)	480 (57%)	140 (63%)
8-10 (mild)	180 (22%)	49 (22%)
11-21 (moderate to severe)	175 (21%)	34 (15%)
**Depression (mean, 95% CI)***	5.4 (5.2-5.7)	4.9 (4.4-5.4)
0-7 (none)	587 (70%)	173 (78%)
8-10 (mild)	171 (20%)	34 (15%)
11-21 (moderate to severe)	77 (9%)	16 (7%)

*Numbers of HADS respondents lower than for main survey (n = 835 ISUOG members, n = 223 Others)

For peer review only

1 **Supplement 7: Comparison of comorbidity in those with (n = 290) and without suspected**
2 **or confirmed SARS-CoV-2 (n = 1947) (Total n = 2237).**

	**Suspected or confirmed SARS-CoV-2**	**Without suspected or confirmed SARS-CoV-2**
Heart disease (n, %)	3 (1%)	43 (2%)
Lung disease (n %)	30 (10%)	160 (8%)
Use of antihypertensives (n, %)	39 (13%)	283 (15%)
Smokers (n, %)	13 (4%)	125 (6%)

3
4

For peer review only

Supplement 8: Comparison of HADS outcome score in those with (n = 162) and without suspected or confirmed SARS-CoV-2 (n = 896) (Total n = 1058).

	**Suspected or confirmed SARS-CoV-2**	**Without suspected or confirmed SARS-CoV-2**
Anxiety (mean, 95% CI)	7.4 (6.7-8.0)	6.8 (6.6-7.1)
0-7 (none)	86 (53%)	534 (60%)
8-10 (mild)	32 (20%)	197 (22%)
11-21 (moderate to severe)	44 (27%)	165 (18%)
Depression (mean, 95% CI)	5.8 (5.2-6.4)	5.2 (5.0-5.5)
0-7 (none)	105 (65%)	655 (73%)
8-10 (mild)	37 (23%)	168 (19%)
11-21 (moderate to severe)	20 (12%)	73 (8%)

For peer review only

1 **Supplement 9: Comparison of HADS outcome score in those with (n = 788) and without**
2 **PPE shortages (n = 270) (Total n = 1058).**

	**PPE shortages**	**No PPE shortages**
Anxiety (mean, 95% CI)	7.4 (7.1-7.7)	5.5 (5.0-6.0)
0-7 (none)	430 (55%)	190 (70%)
8-10 (mild)	183 (23%)	46 (17%)
11-21 (moderate to severe)	175 (22%)	34 (13%)
Depression (mean, 95% CI)	5.7 (5.5-6.0)	4.1 (3.7-4.5)
0-7 (none)	540 (69%)	220 (81%)
8-10 (mild)	167 (21%)	38 (14%)
11-21 (moderate to severe)	81 (10%)	12 (4%)

3

For peer review only

STROBE 2007 (v4) Statement—Checklist of items that should be included in reports of *cross-sectional studies*

Section/Topic	Item #	Recommendation	Reported on page #
**Title and abstract**	1	(*a*) Indicate the study's design with a commonly used term in the title or the abstract	1-2
		(*b*) Provide in the abstract an informative and balanced summary of what was done and what was found	2-3
**Introduction**			
Background/rationale	2	Explain the scientific background and rationale for the investigation being reported	4
Objectives	3	State specific objectives, including any prespecified hypotheses	5
**Methods**			
Study design	4	Present key elements of study design early in the paper	6
Setting	5	Describe the setting, locations, and relevant dates, including periods of recruitment, exposure, follow-up, and data collection	6
Participants	6	(*a*) Give the eligibility criteria, and the sources and methods of selection of participants	6
Variables	7	Clearly define all outcomes, exposures, predictors, potential confounders, and effect modifiers. Give diagnostic criteria, if applicable	7
Data sources/ measurement	8*	For each variable of interest, give sources of data and details of methods of assessment (measurement). Describe comparability of assessment methods if there is more than one group	7
Bias	9	Describe any efforts to address potential sources of bias	*6-8*
Study size	10	Explain how the study size was arrived at	6
Quantitative variables	11	Explain how quantitative variables were handled in the analyses. If applicable, describe which groupings were chosen and why	7-8
Statistical methods	12	(*a*) Describe all statistical methods, including those used to control for confounding	7-8
		(*b*) Describe any methods used to examine subgroups and interactions	7-8
		(*c*) Explain how missing data were addressed	7-8
		(*d*) If applicable, describe analytical methods taking account of sampling strategy	7-8
		(*e*) Describe any sensitivity analyses	7-8
**Results**			

For peer review only

Participants	13*	(a) Report numbers of individuals at each stage of study—eg numbers potentially eligible, examined for eligibility, confirmed eligible, included in the study, completing follow-up, and analysed	9-11
		(b) Give reasons for non-participation at each stage	9-11
		(c) Consider use of a flow diagram	Supplementary information
Descriptive data	14*	(a) Give characteristics of study participants (eg demographic, clinical, social) and information on exposures and potential confounders	9-11
		(b) Indicate number of participants with missing data for each variable of interest	9-11
Outcome data	15*	Report numbers of outcome events or summary measures	9-19
Main results	16	(*a*) Give unadjusted estimates and, if applicable, confounder-adjusted estimates and their precision (eg, 95% confidence interval). Make clear which confounders were adjusted for and why they were included	9-19
		(*b*) Report category boundaries when continuous variables were categorized	9-19
		(*c*) If relevant, consider translating estimates of relative risk into absolute risk for a meaningful time period	9-19
Other analyses	17	Report other analyses done—eg analyses of subgroups and interactions, and sensitivity analyses	9-19
**Discussion**			
Key results	18	Summarise key results with reference to study objectives	20
Limitations	19	Discuss limitations of the study, taking into account sources of potential bias or imprecision. Discuss both direction and magnitude of any potential bias	20
Interpretation	20	Give a cautious overall interpretation of results considering objectives, limitations, multiplicity of analyses, results from similar studies, and other relevant evidence	20-22
Generalisability	21	Discuss the generalisability (external validity) of the study results	20
**Other information**			
Funding	22	Give the source of funding and the role of the funders for the present study and, if applicable, for the original study on which the present article is based	23

*Give information separately for cases and controls in case-control studies and, if applicable, for exposed and unexposed groups in cohort and cross-sectional studies.

Note: An Explanation and Elaboration article discusses each checklist item and gives methodological background and published examples of transparent reporting. The STROBE checklist is best used in conjunction with this article (freely available on the Web sites of PLoS Medicine at http://www.plosmedicine.org/, Annals of Internal Medicine at http://www.annals.org/, and Epidemiology at http://www.epidem.com/). Information on the STROBE Initiative is available at www.strobe-statement.org.

For peer review only

BMJ Open

The experiences and wellbeing of healthcare professionals working in the field of ultrasound in Obstetrics and Gynecology as the SARS-CoV-2 pandemic was evolving: a cross-sectional survey study

Journal:	*BMJ Open*
Manuscript ID	bmjopen-2021-051700.R1
Article Type:	Original research
Date Submitted by the Author:	22-Nov-2021
Complete List of Authors:	Bourne, Tom; Imperial College London, Early Pregnancy and Acute Gynaecology Unit, Department of Obstetrics and Gynaecology, Queen Charlotte's and Chelsea Hospital; KU Leuven, Department of Development and Regeneration<br>Kyriacou, Christopher; Imperial College London, Early Pregnancy and Acute Gynaecology Unit, Department of Obstetrics and Gynaecology, Queen Charlotte's and Chelsea Hospital<br>Shah, Harsha; Imperial College London, Early Pregnancy and Acute Gynaecology Unit, Department of Obstetrics and Gynaecology, Queen Charlotte's and Chelsea Hospital<br>Ceusters, Jolien; KU Leuven, Department of Oncology, Laboratory of Tumour Immunology and Immunotherapy<br>Preisler, Jessica; Universidad del Desarrollo, Facultad de Medicina, Clínica Alemana ; Hospital Clínico de la Universidad de Chile José Joaquín Aguirre, Facultad de Medicina, Clínica Alemana<br>Metzger, Ulrike; Centre d'Échographie de l'Odéon<br>Landolfo, Chiara; Fondazione Policlinico Universitario Agostino Gemelli IRCCS, Dipartimento Scienze della Salute della Donna, del Bambino e di Sanità Pubblica<br>Lees, Christoph; Imperial College London, Centre for Fetal Care, Department of Obstetrics and Gynaecology, Queen Charlotte's and Chelsea Hospital<br>Timmerman, Dirk; KU Leuven, Department of Development and Regeneration; KU Leuven University Hospitals Leuven, Department of Obstetrics and Gynaecology
**Primary Subject Heading**:	Obstetrics and gynaecology
Secondary Subject Heading:	Radiology and imaging, Public health, Obstetrics and gynaecology, Nursing, Global health
Keywords:	Ultrasound < RADIOLOGY & IMAGING, ULTRASONOGRAPHY, GYNAECOLOGY, Ultrasonography < OBSTETRICS, Public health < INFECTIOUS DISEASES

For peer review only - http://bmjopen.bmj.com/site/about/guidelines.xhtml

I, the Submitting Author has the right to grant and does grant on behalf of all authors of the Work (as defined in the below author licence), an exclusive licence and/or a non-exclusive licence for contributions from authors who are: i) UK Crown employees; ii) where BMJ has agreed a CC-BY licence shall apply, and/or iii) in accordance with the terms applicable for US Federal Government officers or employees acting as part of their official duties; on a worldwide, perpetual, irrevocable, royalty-free basis to BMJ Publishing Group Ltd ("BMJ") its licensees and where the relevant Journal is co-owned by BMJ to the co-owners of the Journal, to publish the Work in this journal and any other BMJ products and to exploit all rights, as set out in our licence.

The Submitting Author accepts and understands that any supply made under these terms is made by BMJ to the Submitting Author unless you are acting as an employee on behalf of your employer or a postgraduate student of an affiliated institution which is paying any applicable article publishing charge ("APC") for Open Access articles. Where the Submitting Author wishes to make the Work available on an Open Access basis (and intends to pay the relevant APC), the terms of reuse of such Open Access shall be governed by a Creative Commons licence – details of these licences and which Creative Commons licence will apply to this Work are set out in our licence referred to above.

Other than as permitted in any relevant BMJ Author's Self Archiving Policies, I confirm this Work has not been accepted for publication elsewhere, is not being considered for publication elsewhere and does not duplicate material already published. I confirm all authors consent to publication of this Work and authorise the granting of this licence.

For peer review only

TITLE PAGE

The experiences and wellbeing of healthcare professionals working in the field of ultrasound in Obstetrics and Gynecology as the SARS-CoV-2 pandemic was evolving: a cross-sectional survey study

Authors

Tom Bourne[1,2], Christopher Kyriacou[1], Harsha Shah[1], Jolien Ceusters[3], Jessica Preisler[4,5], Ulrike Metzger[6], Chiara Landolfo[7], Christoph Lees[8], Dirk Timmerman[2,9]

Affiliations

1. Early pregnancy and Acute Gynaecology Unit, Department of Obstetrics and Gynaecology, Queen Charlotte's and Chelsea Hospital, Imperial College, London, UK.
2. Department of Development and Regeneration, KU Leuven, Leuven, Belgium.
3. Department of Oncology, Laboratory of Tumour Immunology and Immunotherapy, KU Leuven, Leuven, Belgium.
4. Facultad de Medicina, Clínica Alemana – Universidad del Desarrollo, Santiago, Chile
5. Hospital Clínico de la Universidad de Chile, Santiago, Chile.
6. Centre d'Échographie de l'Odéon, Paris, France.
7. Dipartimento Scienze della Salute della Donna, del Bambino e di Sanità Pubblica, Fondazione Policlinico Universitario Agostino Gemelli, IRCCS, Rome, Italy.
8. Centre for Fetal Care, Department of Obstetrics and Gynaecology, Queen Charlotte's and Chelsea Hospital, Imperial College, London, UK.
9. Department of Obstetrics and Gynaecology, University Hospitals Leuven, Leuven, Belgium.

Correspondence to:

Professor Tom Bourne

Queen Charlotte's and Chelsea Hospital, Imperial College London

Du Cane Road, London, W12 0HS, UK

t.bourne@imperial.ac.uk

Manuscript word count: 4187

For peer review only

ABSTRACT

Objectives: Assess experience of healthcare professionals (HCPs) working with ultrasound in Obstetrics and Gynecology during the evolving SARS-CoV-2 pandemic given the new and unprecedented challenges involving viral exposure, personal protective equipment (PPE) and wellbeing.

Design: Prospective cross-sectional survey study.

Setting: Online international survey. Single-best, open box and Hospital and Anxiety Depression Scale (HADS) questions.

Participants: The survey was sent to 35,509 HCPs in 124 countries and was open from 7th-21st May 2020. 2237/3237 (69.1%) HCPs from 115 countries who consented to participate completed the survey. 1058 (47.3%) completed the HADS.

Primary outcome measures: Overall prevalence of SARS-CoV-2, depression and anxiety amongst HCPs in relation to country and PPE availability.

Analyses: Univariate analyses were used to investigate associations without generating erroneous causal conclusions.

Results: Confirmed/suspected SARS-CoV-2 prevalence was 13.0%. PPE provision concerns were raised by 74.1% of participants; highest amongst trainees/resident physicians (83.9%), and amongst HCPs in Spain (89.7%). Most participants worked in self-perceived high-risk areas for SARS-CoV-2 (67.5-87.0%), with proportionately more trainees interacting with suspected/confirmed infected patients (57.1% versus 24.2-40.6%) and sonographers seeing more patients who did not wear a mask (33.3% versus 13.9-7.9%). The most frequent PPE combination used were gloves and a surgical mask (22.3%). UK and US respondents reported spending less time self-isolating (8.8 days) and lower satisfaction with their national pandemic response (37.0-43.0%). 19.8% and 8.8% of respondents met the criteria for moderate to severe anxiety and depression respectively.

Conclusions: Reported SARS-CoV-2 HCP prevalence is consistent with literature findings. Most respondents used gloves and a surgical mask, with a greater SARS-CoV-2 prevalence compared with those using ‘full’ PPE. HCPs with the least agency (trainees and sonographers) were not only more likely to see high-risk patients but less likely to be protected. A fifth of respondents reported moderate to severe anxiety.

Keywords: SARS-CoV-2, healthcare professional, ultrasound, anxiety, depression.

For peer review only

STRENGTHS AND LIMITATIONS OF THIS STUDY

- Strengths of our study include the sample size, the assessment of HCP responses internationally and the ability to compare responses between different HCPs.
- Four languages enabled greater participant inclusion and responses between ISUOG and non-ISUOG members were compared prior to grouping for analysis.
- Key weaknesses include possible selection bias due to the method of sample selection and the low population response rate.
- It is important not to draw conclusions about causation as this is a descriptive study performed as the pandemic was evolving.
- At the time of survey participation, the majority of HCP did not have the same access to a swab or antibody test that is now available.

For peer review only

INTRODUCTION

Background: The severe acute respiratory syndrome coronavirus 2 (SARS-CoV-2) pandemic has caused unprecedented challenges to all healthcare systems(1). These include patient protection, managing the burden of disease on service capacity and mitigating adverse effects on healthcare professionals (HCP: physicians, sonographers, nurses and allied HCP)(2).

Guidance internationally has focused on clinician redeployment to front-line settings, prioritization of services, provision and rationalization of personal protective equipment (PPE)(3, 4). During the pandemic, access to PPE for HCP has remained a key concern. Despite HCP being prioritized in most countries, PPE shortages have been described universally(1), and were a particular concern in the early months of the pandemic. Lack of sufficient and adequate PPE is important in Obstetrics and Gynecology where HCP work in a variety of settings, including ultrasound scanning, which may not ordinarily be considered high-risk but involves close patient proximity for extended periods(5). These areas are easily over-looked but may pose a high-risk of SARS-CoV-2 infection(6).

A further concern has been the availability of SARS-CoV-2 testing amongst HCP. Initially, provision for this was variable, with for example relatively easy access in Hong Kong compared to virtually none in the UK. This is important as, in the absence of testing, HCP risk becoming COVID-19 vectors within hospitals and the wider community. Lack of testing may also lead to unnecessary isolation from work. There is limited data regarding HCP testing availability and time HCP have taken off work due to suspected or confirmed SARS-CoV-2(7).

Less attention has been focused on the implications of infectious diseases on HCP psychological health, which previous pandemics have shown to be significant(8, 9). HCP are confronted with ongoing resource, shift pattern, PPE and testing uncertainty(1, 10), as well as COVID-19 exposure risk and the implications of this on their families(11). Protecting the psychological health of the medical workforce is critical, particularly as anxiety, depression and burnout are recognized complications for HCP working in high stress environments(12).

For peer review only

Objectives: We aim to assess the scale, experience and psychological wellbeing of clinicians who have experienced the evolving SARS-CoV-2 pandemic working with ultrasound in Obstetrics and Gynecology internationally between 7th-21st May 2020.

For peer review only

METHODS

Study design: We used a cross-sectional survey design with two main sections assessing physical aspects, including underlying medical conditions, comorbidities and personal SARS-CoV-2 experience, and psychological wellbeing (Supplementary Material 1). Participants were initially asked to provide informed consent prior to continuing to the online survey. If they did not give consent by answering 'yes' to the first question, they were not directed to the survey. Single-best answer and open box questions were proposed by a senior gynaecologist. These were then piloted, vetted and modified by the authors before being translated from English to French, Spanish and Italian in order to increase survey accessibility. We estimated that the time taken to complete the questionnaire was 10 minutes.

Setting: The online platform SurveyMonkey (2020) was selected to upload and disseminate the questionnaire, which was encrypted, and multiple responses were disabled. The survey design ensured the data collected was completely anonymous for all respondents.

Participants: All HCPs on the ISUOG mailing list were invited to participate in this study between 7th and 21st May 2020. There were 1,237,953 confirmed cases of SARS-CoV-2 infection and 63,313 deaths in the time the survey was open(13). Various countries had instigated local and national lockdowns at this time, including the UK. Members were sent an email containing information describing the study and individual links to the questionnaire in English, Spanish, Italian and French. We made it clear to the participants that their participation was voluntary and that responses would be both anonymous and untraceable.

During the survey period, two reminders were sent out. As this was a survey study of a relatively new condition at the time, a power calculation was not performed. Exclusion criteria included retired doctors as this was deemed to not be reflective of the working environment during the SARS-CoV-2 pandemic. A copy of the questionnaire in English can be viewed in Supplementary Material 1.

For peer review only

Patient and Public Involvement statement: Given the HCP participant population and the acute nature of the SARS-CoV-2 pandemic, patient and public involvement was not performed.

Variables and data sources

Physical aspects: This section was divided into sub-sections: Introduction, Demographics, Underlying medical conditions, Medications, Smoking and Body Mass Index (BMI), Role, Shift pattern and PPE, SARS-CoV-2 diagnosis, Personal SARS-CoV-2 pathway, Household, Colleagues and Support (Supplementary Material 1: questions 2 to 40).

Participants were asked details about their job and organizational attributes, as well as on a variety of personal comorbidities relevant to SARS-CoV-2 infection (14). They were then questioned on their personal experience of SARS-CoV-2 at home and in the workplace.

Psychological Wellbeing: Hospital Anxiety and Depression Scale (HADS): Participants were given the option to complete the validated Hospital Anxiety and Depression Scale (HADS), which is a 14-item (7 questions related to anxiety and depression each) questionnaire (15) (Supplementary Material 1: questions 41 to 56). Each subscale measures symptom severity (the score ranges between 0 and 21, a score greater or equal to 11 indicates moderate-to-severe symptoms). If respondents chose not to complete the HADS, the survey ended.

Ethical approval: Ethical approval for the distribution of this online survey was received from the ethics committee at KU Leuven, Belgium (S-64016). A copy of the protocol can be viewed in Supplementary Material 2.

Outcome measures: The primary outcome was the overall prevalence of SARS-CoV-2, depression and anxiety amongst HCPs in relation to country and PPE availability.

Statistical analyses: Univariate analyses were used to investigate the association between country of origin, type of HCP and PPE without generating erroneous causal conclusions. Results were presented as percentages (%), mean, 95% confidence intervals, medians and ranges depending on the variable. Missing data, i.e., questions that were skipped by HCPs as they completed their questionnaire, were not included in the analyses.

For peer review only

HCPs and areas of work were divided into groups to allow reasonable comparable analyses to be performed. For HCPs, there were three main categories: Consultants/Attending physicians (Gynaecologists, Obstetricians, Obstetricians and Gynaecologists or Radiologists/Sonologists aged 35 years and older), Trainees/Residents (Gynaecologists, Obstetricians, Obstetricians and Gynaecologists or Radiologists/Sonologists aged 34 years and younger, doctors in research, doctors in training) and Sonographers (sonographers and allied healthcare professionals [Midwives and Nurses]) (Supplementary Material 3).

Areas of work were defined as high-risk (gynaecology [early pregnancy unit, acute gynaecology, ultrasound], obstetrics [birth centre/birthing centre, labor ward, maternity triage/hospital obstetric triage, ultrasound], radiology [sonology-ultrasound]), moderate risk (obstetrics [antenatal clinic, antenatal ward, community clinics, postnatal ward], radiology [non-ultrasound]), or low-risk (working from home, gynecology [benign, oncology], obstetrics [maternity helplines]) (Supplementary Material 4). This categorisation approximated risk based on HCP proximity and time spent with patients, allowing meaningful data analysis.

Anxiety and depression scores were divided by case-ness in order to differentiate the proportion of the study population that had no anxiety or depression (HADS 0-7), those experiencing mild anxiety or depression (HADS 8-10) and those suffering moderate to severe anxiety or depression (HADS 11-21).

The statistical analyses were performed using R, version 3.5.1.

For peer review only

RESULTS

Summary

Response: The survey was sent to 35,509 HCPs in 124 countries. 3287 (9.3%) HCPs clicked the survey link and 3237 (98.5%) consented to complete it. 1960 (60.5%) responded in English, 881 (27.2%) in Spanish, 267 (8.2%) in Italian and 129 (4.0%) in French. The response rate for ISUOG members was 23.1% (2441/10589) whist non-ISUOG members was 3.2% (796/24920) (Supplementary Material 5). 2237 of 3237 (69.1%) HCPs from 115 countries who started the survey completed it in full. Mean age of respondents was 47.2 years, with a range from 18 to 82 years. The majority (83%) were aged 31-60 years old. 1474 (66%) of respondents were female and 755 (34%) were male, with 4 intersex or undisclosed. 1058/2237 participants (47.3%) completed the HADS and the remainder (1179/2237, 52.7%) did not. Survey participation and demographics are summarised in Table 1.

For peer review only

Table 1: Demographics of total survey participation (n = 3237).

	**Total (n = 3237)**
**Total ISUOG mailing list (n)**	35509
**Response of total ISUOG mailing list (n, %)**	3237 (9%)
Countries (n)	124
Number of respondents in English (n)	1960
Number of respondents in Spanish (n)	881
Number of respondents in Italian (n)	267
Number of respondents in French (n)	129
ISUOG members (n, %)	2441 (23%)
**Completed survey**	**2237**
**ISUOG members (n, %)**	1795 (80%)
**Countries (n)**	115
**Age (mean, range)**	47.2 (18-82)
≤20 years	1 (0%)
21-30 years	117 (5%)
31-40 years	558 (25%)
41-50 years	686 (31%)
51-60 years	596 (27%)
61-70 years	251 (11%)
>70 years	24 (1%)
**Gender (n, %)**	
Female	1474 (66%)
Male	755 (34%)

Intersex	1 (0%)
Prefer not to say	3 (0%)
**Prevalence suspected/confirmed SARS-CoV-2 based on symptoms +/- PCR testing (n, %)**	290 (13%)
**Is PPE used by respondents (n, %)**	
Not yet	279 (12%)
Yes - for aerosol generating procedures only	133 (6%)
Yes - for all patients	1392 (62%)
Yes - for suspected/positive patients only	429 (19%)
**What PPE is used by respondents (n, %)**	
None	76 (3%)
Gloves only	49 (2%)
Gloves + surgical mask	497 (22%)
Gloves + surgical mask + visor	152 (7%)
Gloves + FFP3 mask	95 (4%)
Gloves + FFP3 mask + visor	51 (2%)
Gloves + FFP3 mask + visor + gown + surgical hat	90 (4%)
Patient wearing mask	1841 (82%)
**Concerns regarding a lack of PPE (n, %)**	
No	579 (26%)
Gloves	278 (12%)
Gown	629 (28%)
N95/FFP3 mask	1279 (57%)
Surgical hat	233 (10%)
Surgical mask	820 (37%)
Visor	582 (26%)
Surgical mask (patient)	609 (27%)
**Type of PPE that has been re-used or recycled (n, %)**	
No	865 (39%)
Gloves	34 (2%)
Gown	287 (13%)
N95/FFP3 mask	886 (40%)
Surgical hat	92 (4%)
Surgical mask	565 (25%)
Visor	621 (28%)
**Anxiety (mean, 95% CI)***	6.9 (6.7-7.2)
0-7 (none)	620 (59%)
8-10 (mild)	229 (22%)
11-21 (moderate to severe)	209 (20%)
**Depression (mean, 95% CI)***	5.3 (5.1-5.5)

For peer review only

0-7 (none)	760 (72%)
8-10 (mild)	205 (19%)
11-21 (moderate to severe)	93 (9%)

*Numbers of HADS respondents lower than main survey (n = 1058).

Direct comparison of data between the characteristics of ISUOG members and non-ISUOG members confirmed findings were similar (Supplementary Material 6). It was therefore deemed appropriate to combine these populations for the analysis.

COVID-19 prevalence: Confirmed or suspected SARS-CoV-2 prevalence was 13.0% (290/2237). Amongst this group, 11.4% had a history of heart/lung disease in, 13.4% were taking antihypertensives and 4.5% were smokers (Supplementary Material 7).

PPE use and provision: 12.5% of all HCPs reported not using PPE whilst 87.5% reported variable use. 74.1% had concerns about provision or shortages of one or more items of PPE, in particular N95/FFP3 masks (57.2%). PPE was reported to be recycled or re-used by 61.3%.

Anxiety and depression: 21.6% of participants met the criteria for mild anxiety and 19.8% for moderate to severe anxiety. 19.4% met the criteria for mild and 8.8% for moderate to severe depression. 27.2% and 12.3% of those with suspected/confirmed SARS-CoV-2 reported moderate to severe anxiety and depression, compared to 18.4% and 8.1% of those without (Supplementary Material 8). A greater proportion of female participants scored for moderate to severe anxiety (156/754, 20.7%) and depression (79/754, 10.5%) compared to male participants (41/303, 13.5% and 14/303, 4.6%) (Supplementary Material 9).

4 out of 2237 participants were excluded from the univariate analyses, having completed the survey following retirement.

Univariate analysis by country (n=2233)

A breakdown of findings by countries that had most respondents are presented in Table 2.

For peer review only

1 **Table 2: Breakdown of findings by country (n = 2233).**

	Italy	Spain	India	Philippines	UK	US	Other^
**Number of respondents (n, %)**	174 (8%)	136 (6%)	115 (5%)	114 (5%)	103 (5%)	102 (5%)	1489 (67%)
**Prevalence suspected/ confirmed SARS-CoV-2 based on symptoms +/- PCR testing (n, %)**	26 (15%)	20 (15%)	3 (3%)	10 (9%)	32 (31%)	14 (14%)	185 (12%)
**Number who had SARS-CoV-2 antibody test (n, %)**	92 (53%)	82 (60%)	1 (1%)	21 (18%)	1 (1%)	16 (16%)	155 (10%)
**Number who had positive SARS-CoV-2 antibody (n, %)**	6 (7%)	11 (13%)	0 (0%)	0 (0%)	0 (0%)	4 (25%)	16 (10%)
**Is PPE used by respondents (n, %)**							
Not yet	17 (10%)	28 (21%)	31 (27%)	6 (5%)	7 (7%)	5 (5%)	185 (12%)
Yes – for aerosol generating procedures only	8 (5%)	6 (4%)	9 (8%)	7 (6%)	6 (6%)	3 (3%)	94 (6%)
Yes – for all patients	107 (61%)	33 (24%)	65 (57%)	96 (84%)	76 (74%)	83 (81%)	932 (63%)
Yes – for suspected/positive patients only	42 (24%)	69 (51%)	10 (9%)	5 (4%)	14 (14%)	11 (11%)	278 (19%)
**What PPE is used by respondents (n, %)**							
None	0 (0%)	1 (1%)	1 (1%)	1 (1%)	1 (1%)	0 (0%)	72 (5%)
Gloves only	0 (0%)	0 (0%)	0 (0%)	0 (0%)	2 (2%)	0 (0%)	47 (3%)
Gloves + surgical mask	79 (45%)	42 (31%)	7 (6%)	0 (0%)	41 (40%)	45 (44%)	283 (19%)
Gloves + surgical mask + visor	6 (3%)	11 (8%)	6 (5%)	1 (1%)	17 (17%)	12 (12%)	99 (7%)
Gloves + FFP3 mask	12 (7%)	10 (7%)	8 (7%)	0 (0%)	0 (0%)	6 (6%)	59 (4%)
Gloves + FFP3 mask + visor	1 (1%)	4 (3%)	6 (5%)	2 (2%)	0 (0%)	3 (3%)	35 (2%)
Gloves + FFP3 mask + visor + gown + surgical hat	3 (2%)	5 (4%)	13 (11%)	23 (20%)	0 (0%)	1 (1%)	45 (3%)
Patient wearing mask	173 (99%)	133 (98%)	109 (95%)	111 (97%)	33 (32%)	91 (89%)	1191 (80%)
**Concerns regarding a lack of PPE (n, %)**							
No	35 (20%)	14 (10%)	52 (45%)	20 (18%)	46 (45%)	24 (24%)	388 (26%)
Gloves	25 (14%)	19 (14%)	5 (4%)	12 (11%)	1 (1%)	9 (9%)	207 (14%)
Gown	53 (30%)	45 (33%)	23 (20%)	54 (47%)	25 (24%)	23 (23%)	406 (27%)
N95/FFP3 mask	118 (68%)	110 (81%)	51 (44%)	84 (74%)	33 (32%)	61 (60%)	822 (55%)
Surgical hat	15 (9%)	10 (7%)	10 (9%)	15 (13%)	4 (4%)	8 (8%)	171 (11%)
Surgical mask	84 (48%)	62 (46%)	10 (9%)	21 (18%)	24 (23%)	45 (44%)	574 (39%)
Visor	51 (29%)	51 (38%)	11 (10%)	19 (17%)	19 (18%)	24 (24%)	407 (27%)
Surgical mask (patient)	44 (25%)	47 (35%)	10 (9%)	32 (28%)	13 (13%)	31 (30%)	432 (29%)
**Type of PPE that has been re-used or recycled (n, %)**							
No	55 (32%)	31 (23%)	40 (35%)	25 (22%)	62 (60%)	12 (12%)	640 (43%)
Gloves	2 (1%)	3 (2%)	5 (4%)	2 (2%)	0 (0%)	1 (1%)	21 (1%)
Gown	15 (9%)	17 (13%)	23 (20%)	70 (61%)	3 (3%)	4 (4%)	155 (10%)

For peer review only

N95/FFP3 mask	82 (47%)	82 (60%)	59 (51%)	66 (58%)	10 (10%)	68 (67%)	519 (35%)
Surgical hat	2 (1%)	7 (5%)	1 (1%)	16 (14%)	1 (1%)	4 (4%)	61 (4%)
Surgical mask	83 (48%)	60 (44%)	7 (6%)	13 (11%)	14 (14%)	65 (64%)	323 (22%)
Visor	23 (13%)	48 (35%)	31 (27%)	56 (49%)	28 (27%)	28 (27%)	407 (27%)
**Mean days of self-isolation (range)**	11.3 (0-30)	11.6 (0-30)	17.3 (0-30)	16.9 (0-30)	8.8 (0-30)	8.8 (0-30)	8.4 (0-30)
**Mean days of household self-isolating (range)**	21.1 (14-30)	10.5 (2-15)	18 (10-30)	15.3 (7-30)	11.1 (2-14)	15 (2-30)	12.2 (1-30)
**Number of respondents satisfied with local unit SARS-CoV-2 response (%)**	89 (51%)	97 (71%)	89 (77%)	89 (78%)	75 (73%)	74 (73%)	1018 (68%)
**Mean % level of respondent satisfaction with government SARS-CoV-2 response (95% CI)**	38 (34-41)	23 (19-26)	61 (56-65)	48 (44-52)	43 (38-48)	37 (31-43)	53 (52-55)
**Anxiety (mean, 95% CI)***	6.3 (5.6-7.1)	6.9 (6.1-7.7)	7.4 (6.1-8.8)	7.1 (5.6-8.6)	7.2 (6.2-8.2)	7.3 (6.1-8.4)	6.9 (6.6-7.2)
0-7 (none)	74 (71%)	51 (61%)	22 (51%)	18 (50%)	36 (58%)	35 (59%)	384 (57%)
8-10 (mild)	13 (12%)	21 (25%)	13 (30%)	10 (28%)	12 (19%)	8 (14%)	152 (23%)
11-21 (moderate to severe)	17 (16%)	12 (14%)	8 (19%)	8 (22%)	14 (23%)	16 (27%)	134 (20%)
**Depression (mean, 95% CI)***	5.8 (5.2-6.5)	5 (4.2-5.7)	5.1 (4.1-6.1)	5.3 (4.2-6.5)	5.3 (4.3-6.2)	4.8 (3.8-5.7)	5.3 (5-5.6)
0-7 (none)	74 (71%)	67 (80%)	31 (72%)	27 (75%)	44 (71%)	46 (78%)	471 (70%)
8-10 (mild)	20 (19%)	11 (13%)	10 (23%)	8 (22%)	12 (19%)	9 (15%)	135 (20%)
11-21 (moderate to severe)	10 (10%)	6 (7%)	2 (5%)	1 (3%)	6 (10%)	4 (7%)	64 (10%)

^The 1489 within the 'Other' cohort make up data from participants of the remaining 109 countries. We provide a breakdown of the 6 countries that had the most respondents.
*Numbers of HADS respondents lower than main survey (n Italy = 104, Spain = 84, India = 43, Philippines = 36, UK = 62, US = 59).

COVID-19 prevalence: The highest prevalence of SARS-CoV-2 infection based on symptoms and/or a positive polymerase chain reaction (PCR) swab was reported in the United Kingdom (UK) (31.1%), followed by Italy (14.9%), Spain (14.7%), and the United States (US) (13.7%). Respondents from India and the Philippines reported a prevalence of 2.6% and 8.8% respectively. Only 0.9% of Indian and 1.0% of UK participants had an antibody test, compared with 52.9% and 60.3% of participants in Italy and Spain respectively. 25.0% of participants from the US and 13.4% from Spain who had a SARS-CoV-2 antibody test received a positive result.

Participants in the UK reported spending an average of 8.8 days self-isolating away from the workplace – the same as the US. Mean self-isolation time was only greater than 2 weeks in

For peer review only

India and the Philippines (17.3 and 16.9 days respectively) where SARS-CoV-2 prevalence amongst HCP in the study was lowest at the time of the study.

PPE use and provision: The use of PPE when performing ultrasound was reported by 73.0 to 95.1% of participants. The most common combination of PPE used whilst performing ultrasonography was gloves and a surgical mask, with the highest rates of use in Italy (45.4%), the UK (39.8%) and US (44.1%). No participants from the Philippines and only 6.1% from India reported using only gloves and a surgical mask. Visor use in addition to gloves and a surgical mask were highest in UK (16.5%) and US (11.8%). Full PPE (gloves, FFP3/N95 mask, visor, gown, surgical hat) was used by 4% (90/2233) of respondents. Full PPE use was highest in the Philippines (20.2%) and lowest in the UK (0%). Apart from the UK (32.0%), high rates of patients wearing a mask were noted in every other country analysed (80.0 to 99.4%).

PPE provision or supply concerns were highest in Spain (89.7%), Italy (79.9%) and the Philippines (82.5%) and lowest in the UK (55.3%) and India (54.8%). Less PPE was reported as being recycled or reused in the UK (39.8%) compared to any other country analysed. In the US, 88.2% of HCPs reported PPE recycling or re-use.

Anxiety, depression and support: The HADS revealed that in the UK 22.6%, and in the US 27.1% of participants met the criteria for moderate to severe anxiety, higher than any other country analysed, with Spanish participants having the lowest rate (14.3%). Italian, UK and 'other countries' participants had the highest rates of moderate to severe depression (9.6-9.7%) with Philippines HCPs having the lowest rates (2.8%).

Levels of satisfaction with the support offered by local units during the pandemic ranged from 68% to 78% in all countries except Italy where the level was markedly lower (51.1%). Indians had the highest satisfaction rate with their government response to the pandemic with a mean of 61%, compared with 43% in the UK, 37% in the US, 38% in Italy, and 23% in Spain.

For peer review only

Univariate analysis by HCP (n=2233)

Table 3 focuses on HCPs – 66.3% consultants/attending physicians, 22.3% sonographers and 11.4% trainee/resident physicians (who are the youngest cohort overall). 62.4-63.3% of physicians and 54.3-55.6% of sonographers reported changes to their duties and patient contact throughout the pandemic.

Table 3: Breakdown of findings by healthcare professional (HCP) (n = 2233).

	Consultants / Attending	Sonographer	Trainees / Residents
**Number of respondents (n, %)**	1480 (66%)	499 (22%)	254 (11%)
**Changes in shift pattern as a result of the pandemic (n, %)**			
Unable to work	34 (2%)	21 (4%)	11 (4%)
Change in duties due to SARS-CoV-2 pandemic			
Increased work	185 (13%)	64 (13%)	37 (15%)
Similar work	570 (39%)	228 (46%)	82 (32%)
Decreased work	691 (47%)	186 (37%)	124 (49%)
Change in patient contact due to the SARS-CoV-2 pandemic			
Increased patient contact	114 (8%)	35 (7%)	22 (9%)
Similar patient contact	530 (36%)	212 (42%)	90 (35%)
Decreased patient contact	743 (50%)	214 (43%)	119 (47%)
No patient contact	59 (4%)	17 (3%)	12 (5%)
**Level of COVID-19 risk of working environment reported by HCPs (n, %)**			
High risk of COVID-19 infection	1144 (77%)	337 (68%)	221 (87%)
Moderate risk of COVID-19 infection	128 (9%)	32 (6%)	14 (6%)
Low risk of COVID-19 infection	208 (14%)	130 (26%)	19 (7%)
**Interaction w suspected/confirmed SARS-CoV-2 patients (n, %)**	601 (41%)	121 (24%)	145 (57%)
**Is PPE used by respondents (n, %)**			
Not yet	171 (12%)	75 (15%)	33 (13%)
Yes - for aerosol generating procedures only	89 (6%)	28 (6%)	16 (6%)
Yes - for all patients	934 (63%)	334 (67%)	124 (49%)
Yes - for suspected/positive patients only	286 (19%)	62 (12%)	81 (32%)
**What PPE is used by respondents (n, %)**			
None	47 (3%)	21 (4%)	8 (3%)
Gloves only	20 (1%)	25 (5%)	4 (2%)
Gloves + surgical mask	315 (21%)	105 (21%)	77 (30%)
Gloves + surgical mask + visor	111 (8%)	35 (7%)	6 (2%)
Gloves + FFP3 mask	70 (5%)	13 (3%)	12 (5%)
Gloves + FFP3 mask + visor	41 (3%)	8 (2%)	2 (1%)

For peer review only

Gloves + FFP3 mask + visor + gown + surgical hat	59 (4%)	24 (5%)	7 (3%)
Patient wearing mask	1274 (86%)	333 (67%)	234 (92%)
**Concerns regarding a lack of PPE (n, %)**			
No	398 (27%)	140 (28%)	41 (16%)
Gloves	176 (12%)	63 (13%)	39 (15%)
Gown	403 (27%)	133 (27%)	93 (37%)
N95/FFP3 mask	860 (58%)	239 (48%)	180 (71%)
Surgical hat	142 (10%)	61 (12%)	30 (12%)
Surgical mask	516 (35%)	189 (38%)	115 (45%)
Visor	379 (26%)	122 (24%)	81 (32%)
Surgical mask (patient)	395 (27%)	133 (27%)	81 (32%)
**Type of PPE that has been re-used or recycled (n, %)**			
No	594 (40%)	205 (41%)	66 (26%)
Gloves	22 (1%)	9 (2%)	3 (1%)
Gown	166 (11%)	92 (18%)	29 (11%)
N95/FFP3 mask	596 (40%)	152 (30%)	138 (54%)
Surgical hat	59 (4%)	22 (4%)	11 (4%)
Surgical mask	357 (24%)	116 (23%)	92 (36%)
Visor	401 (27%)	138 (28%)	82 (32%)
**Prevalence suspected/confirmed SARS-CoV-2 based on symptoms +/- PCR testing (n, %)**	187 (13%)	71 (14%)	32 (13%)
**Number who had SARS-CoV-2 antibody test (n, %)**	259 (18%)	55 (11%)	54 (21%)
**Number who had positive SARS-CoV-2 antibody (n, %)**	28 (11%)	5 (9%)	4 (7%)
**Mean days of self-isolation (range)**	9.6 (0-30)	9.7 (0-30)	8.5 (0-30)
**Mean days of household self-isolating (range)**	13 (1-30)	14.3 (2-30)	8.8 (2-14)
**Number of respondents satisfied with local unit SARS-CoV-2 response (%)**	1043 (70%)	340 (68%)	148 (58%)
**Mean % level of respondent satisfaction with government SARS-CoV-2 response (95% CI)**	47 (46-49)	57 (54-59)	44 (40-47)
**Anxiety (mean, 95% CI)***	6.7 (6.4-7)	7.7 (7.1-8.3)	6.9 (6.3-7.6)
0-7 (None)	433 (61%)	112 (52%)	75 (58%)
8-10 (Mild)	151 (21%)	48 (22%)	30 (23%)
11-21 (Moderate to severe)	131 (18%)	54 (25%)	24 (19%)
**Depression (mean, 95% CI)***	5.3 (5-5.5)	5.5 (5-6.1)	5.3 (4.7-5.9)
0-7 (none)	523 (73%)	148 (69%)	89 (69%)
8-10 (mild)	126 (18%)	50 (23%)	29 (22%)
11-21 (moderate to severe)	66 (9%)	16 (7%)	11 (9%)

*Numbers of HADS respondents lower than main survey (Consultant/Attending = 715, Sonographer = 214, Trainees = 129)

For peer review only

COVID-19 prevalence: Suspected or confirmed SARS-CoV-2 prevalence was similar across the groups (12.6 to 14.2%). 87.0% of trainees worked in high-risk areas compared to 77.3% of consultants and 67.5% of sonographers. However, trainees self-isolated for 1 day less on average (8.5 days) compared to consultants and sonographers (9.6 and 9.7 days respectively).

PPE use and provision: The PPE reported in each HCP group as most frequently used when performing ultrasonography was gloves and a surgical mask (21.0-30.3%), with 11.6-15.0% reporting no PPE use. Only 66.7% of patients wore a mask during interactions with sonographers compared with 87.0% when interacting with physicians.

A larger proportion of trainees (57.1%) interacted with suspected or confirmed patients with SARS-CoV-2 compared to consultants (40.6%) and sonographers (24.2%) but were less likely to use PPE for all patients (48.8%), compared with consultants (63.1%) and sonographers (66.9%).

83.9% of trainees reported PPE concerns (compared with 73.1% of consultants and 71.9% of sonographers), with 74.0% re-using or recycling PPE (compared with 59.9% consultants and 58.9% sonographers).

Anxiety, depression and support: Rates of anxiety (39.4-47.7%) and depression (26.9-31.0%) were similar across the HCP groups, with 18.3-25.2% and 7.5-9.2% of participants meeting the criteria for moderate to severe anxiety and depression respectively. Those working in high-risk areas appear more inclined to complete the HADS (Supplementary Material 10). However, rates of mild to severe anxiety are overall similar, with more depression reported by those working in lower risk areas.

Trainees reported lower satisfaction/support from their local unit (58.3%, compared with 70.5% and 68.1% in consultants and sonographers respectively) and government (44%, compared with 47% and 57% in consultants and sonographers respectively).

For peer review only

Univariate analysis by PPE (n=2233)

Table 4 focuses on common combinations of PPE used when performing an ultrasound scan.

Table 4: Breakdown of findings by combinations of personal protective equipment (PPE) (n = 2233)^.

	None	Gloves only	Gloves + surgical mask	Gloves + surgical mask + visor	Gloves + FFP3 mask	Gloves + FFP3 mask + visor	Gloves + FFP3 mask + visor + gown + surgical hat
**Number of respondents (n, %)**	76 (3%)	49 (2%)	497 (22%)	152 (7%)	95 (4%)	51 (2%)	90 (4%)
**Level of COVID-19 risk of working environment reported (n, %)**							
High risk of COVID-19 infection	50 (66%)	34 (69%)	394 (79%)	104 (68%)	60 (63%)	37 (73%)	73 (81%)
Moderate risk of COVID-19 infection	9 (12%)	3 (6%)	38 (8%)	16 (11%)	7 (7%)	6 (12%)	6 (7%)
Low risk of COVID-19 infection	17 (22%)	12 (24%)	65 (13%)	32 (21%)	28 (29%)	8 (16%)	11 (12%)
**Interaction w suspected/confirmed SARS-CoV-2 patients (n, %)**	22 (29%)	16 (33%)	226 (45%)	47 (31%)	31 (33%)	15 (29%)	34 (38%)
**Prevalence suspected/ confirmed SARS-CoV-2 based on symptoms +/- PCR testing (n)**	10 (13%)	14 (29%)	84 (17%)	17 (11%)	10 (11%)	2 (4%)	6 (7%)
**Number of respondents satisfied with local unit SARS-CoV-2 response (%)**	57 (75%)	33 (67%)	326 (66%)	112 (74%)	65 (68%)	38 (75%)	60 (67%)
**Mean % level of respondent satisfaction with government SARS-CoV-2 response (95% CI)**	63 (56-69)	68 (60-76)	50 (47-52)	48 (43-53)	42 (36-48)	40 (32-47)	47 (41-53)

^All possible combinations not included in this table, therefore denominator of table not 2233 (n = 1010). Main relevant PPE combinations described.

COVID-19 prevalence: 2.2% of respondents report using gloves in isolation. 29% of this group reported suspected or confirmed COVID-19.

The most used combination of PPE when performing an ultrasound scan was gloves and a surgical mask (used by 22.3% of respondents). 45.4% (226/497) of participants using this combination of PPE reported interacting with confirmed SARS-Co-V-2 patients, with 16.9% of this group reporting suspected or confirmed COVID-19.

The use of gloves with an FFP3/N95 mask instead of a surgical mask when performing ultrasound was reported by 4.3% (without visor) and 2.3% (with visor) of HCP. 10.5% of

For peer review only

those using gloves and FFP3/N95 mask reported suspected or confirmed COVID-19, compared with 3.9% of those who also used a visor.

4.0% of all HCPs reported using 'full' PPE, 81.1% of whom work in a COVID-19 high-risk setting. 'Full' PPE advised for use by clinicians when interacting with a suspected or confirmed SARS-CoV-2 patient includes gloves, gown, N95/FFP3 mask, a surgical hat and visor. 37.8% of those using 'full' PPE interacted at least once with suspected or confirmed SARS-CoV-2 patients. 6.7% of this group reported suspected or confirmed SARS-CoV-2.

82.4% HCPs reported patients wearing a mask during clinical interactions. This was within a high-risk area of work in 76.5% cases.

Anxiety, depression and support: HCPs that reported PPE shortages had higher rates of anxiety (45.4%) and depression (31.5%) compared to those without shortages (29.6% and 18.5% respectively) (Supplementary Material 11).

Lower satisfaction/support from their local unit ranged between 66-75% with mean level of government satisfaction ranging between 40-68% between the PPE subgroups.

For peer review only

DISCUSSION

Summary: We found that trainees and sonographers are generally more exposed to SARS-CoV-2, with sonographers seeing more patients who did not wear a mask and trainees working the most in higher risk areas. Trainees are thus more likely to interact with suspected or confirmed SARS-CoV-2 patients and have greater PPE concerns with poorer satisfaction and support. The most common PPE combination in use by HCPs were gloves and a surgical mask, with 18% of patients reported to not wear a mask. Prevalence of SARS-CoV-2 was lower amongst HCPs when visors, filtering face masks or 'full' PPE were used. Up to 1 in 5 respondents met the criteria for either moderate to severe anxiety or depression. Our findings suggest that suspected or confirmed SARS-CoV-2 prevalence amongst HCPs working with ultrasound in Obstetrics and Gynaecology was at least 13.0% at the time of the survey, with a higher prevalence in the UK.

Strengths: The main strengths of our study are the relatively large sample size, the assessment of HCP responses internationally and the ability to compare responses between different HCPs. We translated the survey into four languages to include as many potential participants as possible. Furthermore, the response rate of ISUOG members was greater than non-ISUOG members, as expected given the target population. We compared responses between ISUOG and non-ISUOG members to ensure the data could be reliably grouped for the analysis.

Weaknesses: The key weakness of the study was the relatively low response rate and the method of sample selection that introduces possible selection bias when interpreting the results. As this is a descriptive study discussing early experience and wellbeing of HCP working with ultrasound in obstetrics and gynaecology during the evolving pandemic, it is important not to draw conclusions about causation, one reason why univariate analyses were performed. For example, we cannot link use of PPE to levels of anxiety and depression in this study. We combined suspected and confirmed SARS-CoV-2 as, at the time of writing, the majority of HCP did not have the same access to a swab or antibody test that is now available.

For peer review only

COVID-19 prevalence: Although international prevalence varies, at the time of writing confirmed global SARS-CoV-2 cases had surpassed 90 million. The suspected or confirmed SARS-CoV-2 prevalence of 13.0% in this international study is consistent with other published reports on SARS-CoV-2 and HCP, where prevalence ranges from 1.6-22%(16-22). These studies have now been conducted across multiple specialities in many countries, with prevalence determined by PCR as well as antibody testing. One UK study within the field of Obstetrics and Gynaecology identified an HCP SARS-CoV-2 prevalence of 22%(18). Given that the advice for use of PPE is consistent within Obstetrics and Gynaecology(6), the higher prevalence may relate to respondents not using PPE, or not using it correctly. The prevalence suggested by our dataset may be associated with PPE supply concerns, the need to recycle or re-use PPE, a lack of guidance regarding when to use various levels of PPE, or due to many respondents experiencing high-risk interactions with patients, for example scanning in proximity for prolonged periods of time.

PPE use and provision: Countries with greatest concern for PPE from our survey included Spain, Italy, the Philippines and the US, with similarly high rates of PPE recycling. In the UK and India, over half of respondents expressed concern. The SARS-CoV-2 pandemic led to imposed export restrictions as domestic needs increased, demand shock, rush of PPE acquisition and supply chain failures with variation of national healthcare advice(23-26). As an example, China was responsible for mass production of clinical gowns, manufacturing 50% of the world's demand pre-pandemic(23). This all contributed to the soaring global costs of PPE and the financial consequences of obtaining the necessary materials to safely support each healthcare system. In the US, gowns were 7.5 times more expensive in 2020 compared with 2019(24). Face masks were 9 times more costly, N95 respirators were priced 8 times the pre-pandemic rate and gloves cost 2.5 times more(24). PPE concern and recycling may have been a consequence of the logistical and financial complications seen as part of the SARS-CoV-2 response. However, as each healthcare system had varying pre-pandemic funding concerns and constraints, as well as varying HCP advice, these may have also compounded PPE supply and distribution further at a time of extreme need.
More trainee doctors reported working in higher risk areas, as well as being more likely to interact with suspected or confirmed SARS-CoV-2 patients than consultants or sonographers. They also had higher levels of concern regarding PPE, re-using and recycling

For peer review only

more PPE, with a poorer perception of unit and governmental satisfaction and support. It is well documented that HCP burnout relates to anxiety and depression and that burnout is particularly prevalent amongst trainees(27). Fears of infection and death during the pandemic may exacerbate this problem(28) and may also relate to inadequate support or protection.

Sonographers also reported seeing more patients who did not wear a mask. The survey shows a worrying pattern where HCPs with the least agency (trainees and sonographers) were not only more likely to see more high-risk patients but were less likely to be protected. It is perhaps not surprising they expressed greater concerns than other colleagues. PPE issues may relate to systematic financial healthcare consequences previous described, but in this instance may also have local aetiology. Those with least agency also tend to have the least power to instigate change in their department. Concerns may never reach managers and administrators who also may not be clinically trained or orientated. Communication failure may have led to the PPE concerns expressed by HCPs.

Consensus statements, national and international guidance have been published on advised levels of PPE in relation to SARS-CoV-2, with increasing requirements based on clinical risk. At the top end of the scale, disposable gloves, disposable fluid-repellent coverall/gown, filtering face piece respirator and eye/face protection should be used when performing aerosol generating procedures on possible or confirmed cases of SARS-CoV-2(6, 29, 30). 22.3% of study participants reported using gloves and a surgical mask, the combination recommended by the ISUOG consensus statement for HCPs performing ultrasound scans(6). Others reported using various combinations of PPE, some of which is recommended for high-risk, aerosol generating patient-HCP interactions. However, a proportion also reported not using PPE. This variation in PPE may be due to local policy, dependent on wards or clinical areas where HCP duties extend beyond ultrasound, or a result of the PPE supply issues described above. The use of 'full' PPE, or the addition of eye/face protection and/or filtering face piece respirators to gloves is associated with lower reports of suspected or confirmed SARS-CoV-2 when compared with gloves and surgical mask alone. This corroborates literature findings(19, 22).

Anxiety and depression: In our study, just under half of survey respondents completed the HADS questionnaire. This may have been due to a language barrier. However, there is also

For peer review only

stigma related to HCPs seeking help and support, even though it is known HCPs experience high levels of psychological morbidity(31-34). This may have discouraged HCPs to provide information relating to anxiety and depression.

From those that completed the HADS, almost half the respondents reported some level of anxiety and depression. 19.8% and 8.8% experiencing moderate to severe anxiety and depression respectively. Greater proportions were amongst female participants, with more depression reported by those working in lower risk areas. Again, this may relate to the stigma for HCPs reporting psychological morbidity and seeking support, but also may be explained by a completion bias i.e., those with psychological morbidity may be more inclined to complete the HADS to increase awareness of these issues(31-34).

Given the design of this study we must be careful not to relate this to COVID and be aware that there may be selection bias. There are aspects of the Obstetrics and Gynaecology environment which may encourage higher pre-existing levels of anxiety and depression amongst HCPs prior to the SARS-CoV-2 pandemic. The INDIGO study reports two thirds of Obstetrics and Gynecology trainees and consultants in the UK have been personally traumatised by work-related events unrelated to SARS-CoV-2, with 31% of them affected by post-traumatic stress disorder symptoms(35). 14.1% and 7.3% of paediatricians reported mild to severe anxiety and depression using HADS in a survey published prior to the pandemic(36), a specialty that reflects similar intensity.

Our findings strongly support the need for reliable infrastructure that provide HCP counselling and psychological support without stigma in every medical and surgical specialty. In the UK, services such as NHS Practitioner Health and the British Medical Association offer confidential support and protect HCPs at the national level whilst locally, seniors provide regular debriefing sessions. However, provision of local and global support is not consistent and thus movement beyond a culture of HCP stigma is essential(31).

This study provides insight into the experience and wellbeing of clinicians working in the field of ultrasound in Obstetrics and Gynaecology during the early phase of the SARS-CoV-2 pandemic. Although further work is required to unpick the associations of the pandemic, PPE availability, SARS-CoV-2 prevalence and anxiety and depression, we hope our report highlights the importance of provision of PPE and the need for national and international consistency in advice regarding PPE requirements, regulations and use. HCPs all take risk

For peer review only

daily, particularly trainees and sonographers who are generally more exposed to SARS-CoV-2 and less unable to communicate their concerns and needs. As many patients may harbour infection with no or minimal symptoms, there is a real need to ensure HCPs are consistently and adequately supported.

For peer review only

AUTHORS' CONTRIBUTIONS

TB, CK, HS and DT participated in the conception and design of the study. TB, CK and HS created the English version of the survey. JP, UM and CL translated survey to Spanish, French and Italian. JC performed the statistical analysis. TB, CK, HS, JC, CL and DT interpreted the results. TB, CK and HS wrote the initial version of the manuscript. All authors critically revised the manuscript and approved the final version.

FUNDING STATEMENT

TB is supported by the NIHR Imperial Biomedical Research Centre, grant number IS-BRC-1215-20013. CK is supported by Imperial Health Charity, grant number RFPrD1920/116. DT is Fundamental Clinical Researcher of FWO (Research Foundation – Flanders). JC and DT are supported by Research Foundation – Flanders (FWO) grant number G0B4716N and Internal Funds KU Leuven grant number C24/15/037.

DATA AVAILABILITY STATEMENT

All data relevant to the study are included in the article or uploaded as supplementary information. No additional data available.

CONFLICT OF INTEREST STATEMENT

All authors have no conflicts of interest to declare.

For peer review only

REFERENCES

1. The L. COVID-19: protecting health-care workers. Lancet (London, England). 2020;395(10228):922.
2. Adams JG, Walls RM. Supporting the Health Care Workforce During the COVID-19 Global Epidemic. JAMA. 2020.
3. Abu-Rustum RS, Akolekar R, Sotiriadis A, Salomon LJ, Da Silva Costa F, Wu Q, et al. ISUOG Consensus Statement on organization of routine and specialist obstetric ultrasound services in the context of COVID-19. Ultrasound in Obstetrics & Gynecology.n/a(n/a).
4. Bourne T, Kyriacou C, Coomarasamy A, Al-Memar M, Leonardi M, Kirk E, et al. ISUOG Consensus Statement on rationalization of early-pregnancy care and provision of ultrasonography in context of SARS-CoV-2. Ultrasound in Obstetrics & Gynecology.n/a(n/a).
5. Wilson AN, Ravaldi C, Scoullar MJL, Vogel JP, Szabo RA, Fisher JRW, et al. Caring for the carers: Ensuring the provision of quality maternity care during a global pandemic. Women Birth. 2020.
6. Abramowicz JS, Basseal JM, Brezinka C, Dall'Asta A, Deng J, Harrison G, et al. ISUOG Safety Committee Position Statement on use of personal protective equipment and hazard mitigation in relation to SARS-CoV-2 for practitioners undertaking obstetric and gynecological ultrasound. Ultrasound Obstet Gynecol. 2020.
7. Lai J, Ma S, Wang Y, Cai Z, Hu J, Wei N, et al. Factors Associated With Mental Health Outcomes Among Health Care Workers Exposed to Coronavirus Disease 2019. JAMA Netw Open. 2020;3(3):e203976.
8. Lee AM, Wong JG, McAlonan GM, Cheung V, Cheung C, Sham PC, et al. Stress and psychological distress among SARS survivors 1 year after the outbreak. Can J Psychiatry. 2007;52(4):233-40.
9. Bansal P, Bingemann TA, Greenhawt M, Mosnaim G, Nanda A, Oppenheimer J, et al. Clinician Wellness During the COVID-19 Pandemic: Extraordinary Times and Unusual Challenges for the Allergist/Immunologist. J Allergy Clin Immunol Pract. 2020.
10. Albott CS, Wozniak JR, McGlinch BP, Wall MH, Gold BS, Vinogradov S. Battle Buddies: Rapid Deployment of a Psychological Resilience Intervention for Healthcare Workers during the COVID-19 Pandemic. Anesth Analg. 2020.
11. Shanafelt T, Ripp J, Trockel M. Understanding and Addressing Sources of Anxiety Among Health Care Professionals During the COVID-19 Pandemic. JAMA. 2020.
12. Dewey C, Hingle S, Goelz E, Linzer M. Supporting Clinicians During the COVID-19 Pandemic. Ann Intern Med. 2020.
13. WHO. WHO Coronavirus Disease (COVID-19) Dashboard: Word Helath Organization; 2020 [Available from: https://covid19.who.int/.
14. Yang J, Zheng Y, Gou X, Pu K, Chen Z, Guo Q, et al. Prevalence of comorbidities and its effects in patients infected with SARS-CoV-2: a systematic review and meta-analysis. Int J Infect Dis. 2020;94:91-5.
15. Zigmond AS, Snaith RP. The hospital anxiety and depression scale. Acta Psychiatr Scand. 1983;67(6):361-70.
16. Kluytmans-van den Bergh MFQ, Buiting AGM, Pas SD, Bentvelsen RG, van den Bijllaardt W, van Oudheusden AJG, et al. Prevalence and Clinical Presentation of Health Care Workers With Symptoms of Coronavirus Disease 2019 in 2 Dutch Hospitals During an Early Phase of the Pandemic. JAMA Netw Open. 2020;3(5):e209673.

For peer review only

17. Sikkema RS, Pas SD, Nieuwenhuijse DF, O'Toole Á, Verweij J, van der Linden A, et al. COVID-19 in health-care workers in three hospitals in the south of the Netherlands: a cross-sectional study. Lancet Infect Dis. 2020.
18. Khalil A, Hill R, Wright A, Ladhani S, O'Brien P. SARS-CoV-2-specific antibody detection in healthcare workers in a UK maternity Hospital: Correlation with SARS-CoV-2 RT-PCR results. Clin Infect Dis. 2020.
19. Korth J, Wilde B, Dolff S, Anastasiou OE, Krawczyk A, Jahn M, et al. SARS-CoV-2-specific antibody detection in healthcare workers in Germany with direct contact to COVID-19 patients. J Clin Virol. 2020;128:104437.
20. Rivett L, Sridhar S, Sparkes D, Routledge M, Jones NK, Forrest S, et al. Screening of healthcare workers for SARS-CoV-2 highlights the role of asymptomatic carriage in COVID-19 transmission. Elife. 2020;9.
21. Chen Y, Tong X, Wang J, Huang W, Yin S, Huang R, et al. High SARS-CoV-2 antibody prevalence among healthcare workers exposed to COVID-19 patients. J Infect. 2020;81(3):420-6.
22. Chatterjee P, Anand T, Singh KJ, Rasaily R, Singh R, Das S, et al. Healthcare workers & SARS-CoV-2 infection in India: A case-control investigation in the time of COVID-19. Indian J Med Res. 2020;151(5):459-67.
23. Burki T. Global shortage of personal protective equipment. Lancet Infect Dis. 2020;20(7):785-6.
24. Mena Lora A, Ali M, Spencer S, Takhsh E, Krill C, Bleasdale S. Cost of Personal Protective Equipment During the First Wave of COVID-19. Antimicrobial Stewardship & Healthcare Epidemiology2021.
25. Walters W, Collins T, Evans A. Additional cost in personal protective equipment to NHS services during COVID-19: a review of the expense incurred by Aneurin Bevan Healthboards Maxillofacial unit over a one-year period to meet current clinical guidelines. Br J Oral Maxillofac Surg. 2020;58(10):e320-e2.
26. Rowan NJ, Laffey JG. Challenges and solutions for addressing critical shortage of supply chain for personal and protective equipment (PPE) arising from Coronavirus disease (COVID19) pandemic - Case study from the Republic of Ireland. Sci Total Environ. 2020;725:138532.
27. Bourne T, Shah H, Falconieri N, Timmerman D, Lees C, Wright A, et al. Burnout, well-being and defensive medical practice among obstetricians and gynaecologists in the UK: cross-sectional survey study. BMJ Open. 2019;9(11):e030968.
28. Hu D, Kong Y, Li W, Han Q, Zhang X, Zhu LX, et al. Frontline nurses' burnout, anxiety, depression, and fear statuses and their associated factors during the COVID-19 outbreak in Wuhan, China: A large-scale cross-sectional study. EClinicalMedicine. 2020:100424.
29. PHE. COVID-19 personal protective equipment (PPE). https://www.gov.uk/government/publications/wuhan-novel-coronavirus-infection-prevention-and-control/covid-19-personal-protective-equipment-ppe. 2020.
30. WHO. Rational use of personal protective equipment for coronavirus disease (COVID-19) and considerations during severe shortages. https://www.who.int/publications/i/item/rational-use-of-personal-protective-equipment-for-coronavirus-disease-(covid-19)-and-considerations-during-severe-shortages. 2020.
31. Knaak S, Mantler E, Szeto A. Mental illness-related stigma in healthcare: Barriers to access and care and evidence-based solutions. Healthc Manage Forum. 2017;30(2):111-6.

For peer review only

32. Adams EF, Lee AJ, Pritchard CW, White RJ. What stops us from healing the healers: a survey of help-seeking behaviour, stigmatisation and depression within the medical profession. Int J Soc Psychiatry. 2010;56(4):359-70.
33. Wallace JE. Mental health and stigma in the medical profession. Health (London). 2012;16(1):3-18.
34. Wijeratne C, Johnco C, Draper B, Earl J. Doctors' reporting of mental health stigma and barriers to help-seeking. Occup Med (Lond). 2021;71(8):366-74.
35. Slade P, Balling K, Sheen K, Goodfellow L, Rymer J, Spiby H, et al. Work-related post-traumatic stress symptoms in obstetricians and gynaecologists: findings from INDIGO, a mixed-methods study with a cross-sectional survey and in-depth interviews. BJOG. 2020;127(5):600-8.
36. van Steijn ME, Scheepstra KWF, Yasar G, Olff M, de Vries MC, van Pampus MG. Occupational well-being in pediatricians-a survey about work-related posttraumatic stress, depression, and anxiety. Eur J Pediatr. 2019;178(5):681-93.

For peer review only

TABLES AND SUPPLEMENTARY MATERIAL

Table 1: Demographics of total survey participation (n = 3237).
Table 2: Breakdown of findings by country (n = 2233).
Table 3: Breakdown of findings by healthcare professional (HCP) (n = 2233).
Table 4: Breakdown of findings by combinations of personal protective equipment (PPE) (n = 2233).

Supplement 1: The ISUOG SARS-CoV-2 Questionnaire for healthcare professionals working with ultrasound in Obstetrics and Gynecology.
Supplement 2: Original study protocol.
Supplement 3: Breakdown of HCP roles for analysis.
Supplement 4: Breakdown of risk by area of work for analysis.
Supplement 5: Flowchart summarizing survey participants, differentiating ISUOG members and non-ISUOG members (n = 3287).
Supplement 6: Demographics of survey participation, differentiated based on ISUOG membership (n = 3237).
Supplement 7: Comparison of comorbidity in those with (n = 290) and without suspected or confirmed SARS-CoV-2 (n = 290) (Total n = 2237).
Supplement 8: Comparison of HADS outcome score in those with (n = 162) and without suspected or confirmed SARS-CoV-2 (n = 896) (Total n = 1058).
Supplement 9: HADS breakdown by gender (n = 1058).
Supplement 10: HADS breakdown by risk of working area (n = 1058).
Supplement 11: Comparison of HADS outcome score in those with (n = 788) and without PPE shortages (n = 270) (Total n = 1058).

For peer review only

SUPPLEMENTARY MATERIAL

The experiences and wellbeing of healthcare professionals working in the field of ultrasound in Obstetrics and Gynecology as the SARS-CoV-2 pandemic was evolving: a cross-sectional survey study

Authors

Tom Bourne[1,2], Christopher Kyriacou[1], Harsha Shah[1], Jolien Ceusters[3], Jessica Preisler[4,5], Ulrike Metzger[6], Chiara Landolfo[7], Christoph Lees[8], Dirk Timmerman[2,9]

Affiliations

1. Early pregnancy and Acute Gynaecology Unit, Department of Obstetrics and Gynaecology, Queen Charlotte's and Chelsea Hospital, Imperial College, London, UK.
2. Department of Development and Regeneration, KU Leuven, Leuven, Belgium.
3. Department of Oncology, Laboratory of Tumour Immunology and Immunotherapy, KU Leuven, Leuven, Belgium.
4. Facultad de Medicina, Clínica Alemana – Universidad del Desarrollo, Santiago, Chile
5. Hospital Clínico de la Universidad de Chile, Santiago, Chile.
6. Centre d'Échographie de l'Odéon, Paris, France.
7. Dipartimento Scienze della Salute della Donna, del Bambino e di Sanità Pubblica, Fondazione Policlinico Universitario Agostino Gemelli, IRCCS, Rome, Italy.
8. Centre for Fetal Care, Department of Obstetrics and Gynaecology, Queen Charlotte's and Chelsea Hospital, Imperial College, London, UK.
9. Department of Obstetrics and Gynaecology, University Hospitals Leuven, Leuven, Belgium.

Correspondence to:

Professor Tom Bourne
Queen Charlotte's and Chelsea Hospital, Imperial College London
Du Cane Road, London, W12 0HS, UK
t.bourne@imperial.ac.uk

For peer review only

Supplement 1: The ISUOG SARS-CoV-2 Questionnaire for healthcare professionals working with ultrasound in Obstetrics and Gynecology.

<u>Informed consent</u>

This questionnaire is fully anonymous - answers cannot be traced back to participants. The anonymised data will be collected for analysis in order to provide a report and publication at a later date.

1. **Please click 'yes' to participate in this survey. Otherwise, click 'no': (please choose one option)**

Yes
No

<u>Introduction</u>

2. **What title best describes your job: (please choose one option)**

Sonographer – Obstetrics
Sonographer – Gynecology
Sonographer – Obstetrics and Gynecology
Obstetrician
Gynecologist
Obstetrician and Gynecologist
Radiologist
Doctor in training – Obstetrics
Doctor in training – Gynecology
Doctor in training – Obstetrics and Gynecology
Doctor in training – Radiologist
Doctor in research – Obstetrics
Doctor in research – Gynecology
Doctor in research – Obstetrics and Gynecology
Doctor in research – Radiology
Nurse – Obstetrics
Nurse – Gynecology
Nurse – Obstetrics and Gynecology
Nurse – Early pregnancy only
Midwife – Obstetrics
Midwife – Early pregnancy only
Retired

3. **In what country do you practice? (please state below)**

Afghanistan
Albania
Algeria
Andorra
Angola
Antigua and Barbuda

For peer review only

1 Argentina
2 Armenia
3 Australia
4 Austria
5 Azerbaijan
6 The Bahamas
7 Bahrain
8 Bangladesh
9 Barbados
10 Belarus
11 Belgium
12 Belize
13 Benin
14 Bhutan
15 Bolivia
16 Bosnia and Herzegovina
17 Botswana
18 Brazil
19 Brunei
20 Bulgaria
21 Burkina Faso
22 Burundi
23 Cabo Verde
24 Cambodia
25 Cameroon
26 Canada
27 Central African Republic
28 Chad
29 Chile
30 China
31 Colombia
32 Comoros
33 Congo, Democratic Republic of the
34 Congo, Republic of the
35 Costa Rica
36 Côte d'Ivoire
37 Croatia
38 Cuba
39 Cyprus
40 Czech Republic
41 Denmark
42 Djibouti
43 Dominica
44 Dominican Republic
45 East Timor (Timor-Leste)
46 Ecuador
47 Egypt

For peer review only

El Salvador
Equatorial Guinea
Eritrea
Estonia
Eswatini
Ethiopia
Fiji
Finland
France
Gabon
The Gambia
Georgia
Germany
Ghana
Greece
Grenada
Guatemala
Guinea
Guinea-Bissau
Guyana
Haiti
Honduras
Hungary
Iceland
India
Indonesia
Iran
Iraq
Ireland
Israel
Italy
Jamaica
Japan
Jordan
Kazakhstan
Kenya
Kiribati
Korea, North
Korea, South
Kosovo
Kuwait
Kyrgyzstan
Laos
Latvia
Lebanon
Lesotho
Liberia

For peer review only

Libya
Liechtenstein
Lithuania
Luxembourg
Madagascar
Malawi
Malaysia
Maldives
Mali
Malta
Marshall Islands
Mauritania
Mauritius
Mexico
Micronesia, Federated States of
Moldova
Monaco
Mongolia
Montenegro
Morocco
Mozambique
Myanmar (Burma)
Namibia
Nauru
Nepal
Netherlands
New Zealand
Nicaragua
Niger
Nigeria
North Macedonia
Norway
Oman
Pakistan
Palau
Panama
Papua New Guinea
Paraguay
Peru
Philippines
Poland
Portugal
Qatar
Romania
Russia
Rwanda
Saint Kitts and Nevis

For peer review only

Saint Lucia
Saint Vincent and the Grenadines
Samoa
San Marino
Sao Tome and Principe
Saudi Arabia
Senegal
Serbia
Seychelles
Sierra Leone
Singapore
Slovakia
Slovenia
Solomon Islands
Somalia
South Africa
Spain
Sri Lanka
Sudan
Sudan, South
Suriname
Sweden
Switzerland
Syria
Taiwan
Tajikistan
Tanzania
Thailand
Togo
Tonga
Trinidad and Tobago
Tunisia
Turkey
Turkmenistan
Tuvalu
Uganda
Ukraine
United Arab Emirates
United Kingdom
United States
Uruguay
Uzbekistan
Vanuatu
Vatican City
Venezuela
Vietnam
Yemen

For peer review only

Zambia
Zimbabwe

4. **Are you an ISUOG member? (please choose one option)**

Yes
No

5. **If you answered no, which society or professional body did you receive this survey from?**

Open box answer

Demographics

6. **How old are you? (please state number of years)**

Numbers offered

7. **What sex were you assigned at birth? (please choose one option)**

Male
Female
Intersex
Prefer not to say

8. **What gender do you most identify with? (please choose one option)**

Male
Female
Prefer not to say
Other (please specify)

Underlying medical conditions

9. **Do you have any underlying medical conditions that place you at increased risk during the SARS-CoV-2 pandemic? (please tick all that apply)**

None
Heart disease
Hypertension
Diabetes
Asthma
Other lung disease
Kidney disease
Cancer

For peer review only

Medication

10. Are you taking any of the following medications? (please tick all that apply)

None
Immunosuppressant medication - steroids
Immunosuppressant medication - methotrexate
Immunosuppressant medication - biological agents
Aspirin
Indomethacin
Any other non-steroidal anti-inflammatory drugs (NSAIDS) e.g. ibuprofen, diclofenac, naproxen
Angiotensin converting enzyme inhibitors (ACEi) blood pressure medication - any ending in -pril (e.g. enalapril)
Any other blood pressure medication e.g. angiotensin receptor blockers (ARB), alpha blockers, alpha-2 receptor agonists, beta blockers, combined alpha and beta blockers, calcium channel blockers (CCB), diuretics, other

Smoking and BMI

11. Do you smoke? (please choose one option)

Yes
Previously – now stopped
No

12. What was your last measured weight? (please choose one option)

In kilograms (kg) – numbers offered

13. What is your height? (please choose one option)

In centimetres (cm) – numbers offered

Role, shift pattern, PPE

14. How has your role and shift pattern been affected by the SARS-CoV-2 pandemic? (please choose one option)

Unable to work for the duration of the pandemic
Reduced duties with no patient contact
Reduced duties with reduced patient contact
Reduced duties with similar patient contact
Reduced duties with increased patient contact
Similar duties with no patient contact
Similar duties with reduced patient contact
Similar duties with similar patient contact
Similar duties with increased patient contact
Increased duties with no patient contact
Increased duties with reduced patient contact
Increased duties with similar patient contact

For peer review only

Increased duties with increased patient contact

15. Where are you working during the SARS-CoV-2 pandemic? (please tick all that apply)

Obstetrics – Labor ward
Obstetrics – Birth center
Obstetrics – Antenatal ward
Obstetrics – Postnatal ward
Obstetrics – Maternity triage
Obstetrics – Antenatal clinic
Obstetrics – Maternity helplines
Obstetrics – Community clinics
Obstetrics – Ultrasound
Gynecology – Acute gynecology
Gynecology – Benign gynecology
Gynecology – Oncology
Gynecology – Ultrasound
Early pregnancy unit – Ultrasound and assessment
Radiology – Ultrasound
Radiology – non-ultrasound
Obstetrics/Gynecology/Radiology – From home

16. Do you interact with patients confirmed or suspected to have SARS-CoV-2? (please choose one option)

Yes – every day
Yes – every week
Yes – every so often
I don't know
Not yet
No

17. Have you been using personal protective equipment (PPE) since the SARS-CoV-2 outbreak? (please choose one option)

No
Not yet
Yes – for aerosol generating procedures only
Yes – for suspected/positive patients only
Yes – for all patients

18. When you perform an ultrasound scan please state what PPE you use: (please tick all that apply)

None
Gloves
Surgical mask
N95/FFP3 mask
Gown
Visor

For peer review only

Surgical hat

19. Have there been any concerns regarding PPE provision or shortages since the SARS-CoV-2 outbreak? (note – Full PPE includes a N95/FFP3 mask, visor, surgical hat, gown and gloves) (please tick all that apply)

No issues with provision
Yes – supply issues with gloves
Yes – supply issues with surgical masks
Yes – supply issues with N95/FFP3 masks
Yes – supply issues with gowns
Yes – supply issues with visors
Yes – supply issues with surgical hats
Yes – supply issues with providing patients with a surgical mask

20. Have you been asked to re-use or recycle any PPE? (please tick all that apply option)

No
Yes - Gloves
Yes - Surgical mask
Yes - N95/FFP3 mask
Yes - Gown
Yes - Visor
Yes - Surgical hat

21. Do you ask patients to wear a surgical mask? (please choose one option)

Yes
No

<u>SARS-CoV-2 diagnosis</u>

22. Have you had or do you have SARS-CoV-2 based on clinical symptoms and/or a SARS-CoV-2 swab (PCR) diagnosis? (please choose one option)

No – no test or symptoms suggestive of SARS-CoV-2
Yes – suspected based on symptoms but have not had PCR swab testing
Yes – suspected based on symptoms, had PCR testing, which was negative
Yes – confirmed on PCR swab testing but have had no symptoms
Yes – suspected based on symptoms and confirmed on PCR swab testing

23. With regards to having a SARS-CoV-2 swab test, please choose one option:

Not been offered
Offered as experiencing symptoms
Offered as in contact with confirmed or probable SARS-CoV-2 patients
Offered as living with someone who has probable or confirmed SARS-CoV-2
Not applicable

For peer review only

24. What symptoms have you experienced or are you experiencing? (please tick all that apply)

None
High fever
Persistent dry cough
Productive cough
Shortness of breath/Difficulty breathing
Fatigue
Headache
Loss of sense of smell and/or taste
Sore throat
Chest pain
Diarrhoea
Myalgia
Loss of appetite
Hoarse voice
Abdominal pain

<u>**Personal SARS-CoV-2 pathway**</u>

25. At what point were you offered a SARS-CoV-2 swab? (please state number of days into your illness with day 1 being the day you started to have symptoms. If you have not had symptoms – please choose 'not had symptoms'. If you were not offered a test, please choose ‘not offered a test’)

Not had symptoms
Not offered a test
Not applicable
Numbers offered

26. How severe was/is your infection? (please choose one option)

Mild COVID-19 (T< 37.8 oC, tiredness, muscle aches, headache, no breathlessness, normal appetite)
Moderate COVID-19 (T> 37.8 oC, troublesome cough, breathless on exercise, tired but no chest or abdominal pain)
Severe COVID-19 (T> 37.8 oC , noticeably breathless even when sitting still, unable to complete sentences when talking, chest or abdominal pain, reduced appetite)
Critical COVID-19 (development of severe acute respiratory syndrome in need of ventilator or other respiratory support)
Not applicable

27. How long were you or have you been symptomatic for? (please state number of days. If you have not had symptoms – please choose 'not had symptoms')

Not had symptoms
Numbers offered

For peer review only

28. How long did you stay at home before going back to work, or if current infection, how long have you been at home for? (please state number of days. If you have not had symptoms – please choose 'not had symptoms')

Not had symptoms
Numbers offered

29. Did you or do you feel under pressure from your employers to return to work before making a full recovery? (please choose one option)

Yes
No
Not applicable

30. If you have been unwell with presumed or confirmed SARS-CoV-2, how long were you advised to stay at home by your employers before going back to work? (please state number of days. If you have not had symptoms – please choose 'not had symptoms')

Not had symptoms
Numbers offered

31. Have you had a SARS-CoV-2 antibody test? (please choose one option)

No – no test or symptoms suggestive of SARS-CoV-2
No – not offered
No – offered and declined
Yes – negative
Yes – positive

Household

32. Have you needed to, or are you currently self-isolating due to a member of your household having suspected or confirmed SARS-CoV-2? (please choose one option)

Yes
No

33. How long did you need or have you needed to self-isolate due to a member of your household having suspected or confirmed SARS-CoV-2? (please state number of days. If your household has been well - please choose 'household has been well')

Household has been well
Numbers offered

Colleagues

34. **How many colleagues do you work with in your unit? (please state number of each group. Select 'unsure' if you do not know)**

Sonographers
Consultants
Doctors in training
Doctors in research
Nurses
Midwifes
Unsure
Numbers offered

35. **To your knowledge, how many colleagues are you aware of in your unit that have had a presumed diagnosis of SARS-CoV-2 based on symptoms or a positive swab? (please state number of each group. Select 'unsure' if you have no information)**

Sonographers
Consultants
Doctors in training
Doctors in research
Nurses
Midwifes
Unsure
Numbers offered

36. **To your knowledge, how many colleagues have needed to self-isolate or are self-isolating due to a member of their household having suspected or confirmed SARS-CoV-2? (please state number of each group. Select 'unsure' if you have no information)**

Sonographers
Consultants
Doctors in training
Doctors in research
Nurses
Midwifes
Unsure
Numbers offered

37. **What is the longest period of time any of your colleagues have taken off in isolation because they or a household member have suspected or confirmed SARS-CoV-2? (please state number of days. Select 'unsure' if you have no information)**

Unsure
Numbers offered

For peer review only

Support

38. **Have you felt or do you feel adequately supported by your unit during the SARS-CoV-2 pandemic? (please choose one option)**

Yes
No

39. **How do you rate the response and support that was provided by your country's Ministry of Health or Public Health officials? (please answer using the scale below)**

0 - Extremely poor
50 - Adequate
100 - Excellent

40. **Please tell us, if you wish, your views regarding how well clinician safety has been protected by your employers (please state in the text box below. If you choose not to answer, please write 'not applicable')**

Open box answer

Hospital Anxiety and Depression Scale (HADS)

41. **Thank you for filling out this questionnaire.**

Optional: Hospital Anxiety and Depression Scale (HADS). If you are willing to proceed, please click yes. Otherwise, click no to end the survey.

Yes
No

Hospital Anxiety and Depression Scale (HADS)

42. **Choose the reply that is closest to how you have been feeling in the past week. Don't take too long over your replies: your immediate is best.**

I feel tense or 'wound up':

Most of the time
A lot of the time
From time to time, occasionally
Not at all

43. **Choose the reply that is closest to how you have been feeling in the past week. Don't take too long over your replies: your immediate is best.**

I still enjoy the things I used to enjoy:

Definitely as much
Not quite as much

For peer review only

Only a little
Hardly at all

44. Choose the reply that is closest to how you have been feeling in the past week. Don't take too long over your replies: your immediate is best.

I get a sort of frightened feeling as if something awful is about to happen:
Very definitely and quite badly
Yes, but not too badly
A little, but it doesn't worry me
Not at all

45. Choose the reply that is closest to how you have been feeling in the past week. Don't take too long over your replies: your immediate is best.

I can laugh and see the funny side of things:
As much as I always could
Not quite so much now
Definitely not so much now
Not at all

46. Choose the reply that is closest to how you have been feeling in the past week. Don't take too long over your replies: your immediate is best.

Worrying thoughts go through my mind:
A great deal of the time
A lot of the time
From time to time, but not too often
Only occasionally

47. Choose the reply that is closest to how you have been feeling in the past week. Don't take too long over your replies: your immediate is best.

I feel cheerful:
Not at all
Not often
Sometimes
Most of the time

48. Choose the reply that is closest to how you have been feeling in the past week. Don't take too long over your replies: your immediate is best.

I can sit at ease and feel relaxed:
Definitely
Usually
Not often
Not at all

For peer review only

49. Choose the reply that is closest to how you have been feeling in the past week. Don’t take too long over your replies: your immediate is best.

I feel as if I am slowed down:
Nearly all the time
Very often
Sometimes
Not at all

50. Choose the reply that is closest to how you have been feeling in the past week. Don’t take too long over your replies: your immediate is best.

I get a sort of frightened feeling like 'butterflies' in the stomach:
Not at all
Occasionally
Quite often
Very often

51. Choose the reply that is closest to how you have been feeling in the past week. Don’t take too long over your replies: your immediate is best.

I have lost interest in my appearance:
Definitely
I don't take as much care as I should
I may not take quite as much care
I take just as much care as ever

52. Choose the reply that is closest to how you have been feeling in the past week. Don’t take too long over your replies: your immediate is best.

I feel restless as I have to be on the move:
Very much indeed
Quite a lot
Not very much
Not at all

53. Choose the reply that is closest to how you have been feeling in the past week. Don’t take too long over your replies: your immediate is best.

I look forward with enjoyment to things:
As much as I ever did
Rather less than I used to
Definitely less than I used to
Hardly at all

For peer review only

54. **Choose the reply that is closest to how you have been feeling in the past week. Don't take too long over your replies: your immediate is best.**

I get sudden feelings of panic:
Very often indeed
Quite often
Not very often
Not at all

55. **Choose the reply that is closest to how you have been feeling in the past week. Don't take too long over your replies: your immediate is best.**

I can enjoy a good book or radio or TV program:
Often
Sometimes
Not often
Very seldom

56. **Thank you for filling out this questionnaire. Please complete last question to end. Please tell us, if you wish, how you have felt emotionally during the SARS-CoV-2 pandemic? (please state in the text box below. If you choose not to answer, please write 'not applicable')**

Open box answer

For peer review only

Supplement 2: Original study protocol

Title: An international survey of the impact of COVID19 on healthcare professionals carrying out ultrasonography, service provision and mental state in Obstetrics and Gynecology

Date: 19th April 2020

Main Sponsor: UZ Leuven Belgium
Carried out in collaboration with: International Society of Ultrasound in Obstetrics and Gynecology (ISUOG)
Version number: 1
Principal Investigators**:** Professor Tom Bourne and Professor Dirk Timmerman
Co-Investigators: Dr Christopher Kyriacou, Dr Harsha Shah
Protocol to be assessed by: EC Onderzoek UZ Leuven, Belgium.

Design: Prospective cohort observational survey study.

Aim: To assess the services and psychological impact of the COVID-19 pandemic to healthcare professionals carrying out ultrasonography within the Obstetrics and Gynecology specialty.

Hypothesis:

1. Service provision to support healthcare professionals during the COVID-19 pandemic varies considerably in every unit globally;
2. Healthcare professionals will have experienced COVID-19 first-hand;
3. The COVID-19 pandemic has caused significant psychological distress to healthcare professionals which can be assessed using a validated psychological tool.

Outcome measures:

1. A questionnaire to healthcare professionals detailing medical history, role, area of work, personal protective equipment (PPE), COVID-19 patient contact and personal COVID-19 experience will allow data to be collected in relation to the physical impact of the COVID-19 pandemic;
2. Use of the validated Hospital Anxiety and Depression Scale (HADS) will allow psychological morbidity of healthcare professionals to be assessed.

Eligibility: Members of ISUOG that fill in the questions via an online survey platform. The questionnaire will in English, Spanish, Greek, Hindu, Mandarin and Cantonese.

Duration: The survey will be conducted over a period of two weeks in order to allow time for the questions to be answered, two reminder emails will be sent – one at a week before closure and one 24 hours before closure

Consent: Consent will be implied when the questionnaire is filled in and there will be a specific question asking for consent to take part in the survey, however all information is anonymized and cannot be traced to an individual. No data will be requested that is in breach of General Data Protection Regulation (GDPR).

For peer review only

Background: The current coronavirus (SARS-CoV-2) pandemic carries a number of new challenges to local, national and international healthcare agencies in order to protect patients and healthcare professionals (physicians, sonographers, allied healthcare professionals). Guidance varies from nation to nation with issues arising from redeployment of clinicians, prioritization of particular services and provision of PPE. This is important as those carrying out ultrasound scans are in close proximity to patients and are at high risk of SARS-CoV-2 infection[1].
Anxiety and depression are recognized complications for healthcare professionals working in a high-stress environment, particularly one that now encompasses a novel disease for which we have yet to fully understand[2].

Methods: This is a prospective cohort study and will recruit via the dissemination of a survey online through the ISUOG mailing list. Consent will be presumed when the form is filled in and submitted. This will allow healthcare professionals globally to answer. The questionnaire has been devised to assess the service provision in units around the world as well as question personal COVID-19 experience. Following on from this will be an optional HADS. The HADS is a 14-part questionnaire with 7 questions exploring symptoms of anxiety and depression, respectively. Each question is answered with a scale of 0-3 to represent severity of symptoms, with an independent maximum score of 21 for depression and anxiety. A score ≥11 is deemed moderate, whilst ≥16 represents severe symptoms[3].

Statistics and data analysis: Sample size will be dependent on the number of online responders. We hope that there will be significant uptake from a number of countries globally. The recruitment will be online so no breach to social distancing rules will apply. To compare differences in binary variables we will use the likelihood ratio, chi-squared or Fisher's exact test and will consider a p value <0.05 as statistically significant. Ninety-five per cent confidence intervals for rates of psychological morbidity will be created using the Wilson method.

Dissemination: Following the collection and analysis of the data obtained from the questionnaire, we aim to present the findings to the ISUOG membership and publish in peer-reviewed journals – preferably Ultrasound in Obstetrics and Gynecology. The need to understand the experience of the pandemic by frontline workers is extremely important, not only to support them now, but to prepare for any future international healthcare emergency.

References

1. Abramowicz JS, Basseal JM, Brezinka C, Dall'Asta A, Deng J, Harrison G, Marsal K, Lee JCS, Lim A, Miloro P, Poon LC, Salvesen KJ, Sande R, ter Haar G, Westerway SC, Xie MX, Lees C. ISUOG Safety Committee Position Statement on use of personal protective equipment and hazard mitigation in relation to SARS-CoV-2 for practitioners undertaking obstetric and gynecological ultrasound. *ISUOG*. 2020 March 28. Available from: https://www.isuog.org/uploads/assets/b8dde768-08a2-424c-a4615551637515e9/ISUOG-Safety-Committee-statement-on-COVID19-and-PPE.pdf
2. Neto MLR, Almeida HG, Esmeraldo JD, Nobre CB, Pinheiro WR, de Oliveira CRT, Sousa IDC, Lima OMML, Lima NNR, Moreira MM, Lima CKT, Junior JG, da Silva CGL.

For peer review only

When health professionals look death in the eye: the mental health of professionals who deal daily with the 2019 coronavirus outbreak. *Psychiatry Res*. 2020 April 13;288:112972. doi:10.1016/j.psychres.2020.112972.

3. Zigmond AS, Snaith RP. The hospital anxiety and depression scale. Acta psychiatrica scandinavica. 1983 Jun;67(6):361-70.

For peer review only

1 **Supplement 3: Breakdown of HCP roles for analysis.**

**Job title**	
Doctor in research – Gynecology	Split up based on age: trainees/residents and consultants/attending
Doctor in research – Obstetrics	Split up based on age: trainees/residents and consultants/attending
Doctor in research – Obstetrics and Gynecology	Split up based on age: trainees/residents and consultants/attending
Doctor in research – Radiology/Sonologist	Split up based on age: trainees/residents and consultants/attending
Doctor in training – Gynecology	Split up based on age: trainees/residents and consultants/attending
Doctor in training – Obstetrics	Split up based on age: trainees/residents and consultants/attending
Doctor in training – Obstetrics and Gynecology	Split up based on age: trainees/residents and consultants/attending
Doctor in training – Radiologist/Sonologist	Split up based on age: trainees/residents and consultants/attending
Gynecologist	Split up based on age: trainees/residents and consultants/attending
Midwife – Early pregnancy only	Sonographer
Midwife – Obstetrics	Sonographer
Nurse – Early pregnancy only	Sonographer
Nurse – Gynecology	Sonographer
Nurse – Obstetrics	Sonographer
Nurse – Obstetrics and Gynecology	Sonographer
Obstetrician	Split up based on age: trainees/residents and consultants/attending
Obstetrician and Gynecologist	Split up based on age: trainees/residents and consultants/attending
Radiologist/Sonologist	Split up based on age: trainees/residents and consultants/attending
Retired	Exclude from analyses
Sonographer – Gynecology	Sonographer
Sonographer – Obstetrics	Sonographer
Sonographer – Obstetrics and Gynecology	Sonographer

For peer review only

1 **Supplement 4: Breakdown of risk by area of work for analysis.**

Areas of work	Risk of COVID-19 infection
Early pregnancy unit - Ultrasound and assessment	High risk
Gynecology - Acute Gynecology	High risk
Gynecology - Benign Gynecology	Low risk
Gynecology – Oncology	Low risk
Gynecology - Ultrasound	High risk
Obstetrics/Gynecology/Radiology - From home	Low risk
Obstetrics - Antenatal clinic	Moderate risk
Obstetrics - Antenatal ward	Moderate risk
Obstetrics - Birth center/Birthing center	High risk
Obstetrics - Community clinics	Moderate risk
Obstetrics - Labor ward	High risk
Obstetrics - Maternity helplines	Low risk
Obstetrics - Maternity triage/Hospital obstetric triage	High risk
Obstetrics - Postnatal ward	Moderate risk
Obstetrics – Ultrasound	High risk
Radiology - non-Ultrasound	Moderate risk
Radiology - Ultrasound	High risk

2
3

For peer review only

Supplement 5: Flowchart summarizing survey participants, differentiating ISUOG members and non-ISUOG members (n = 3287).

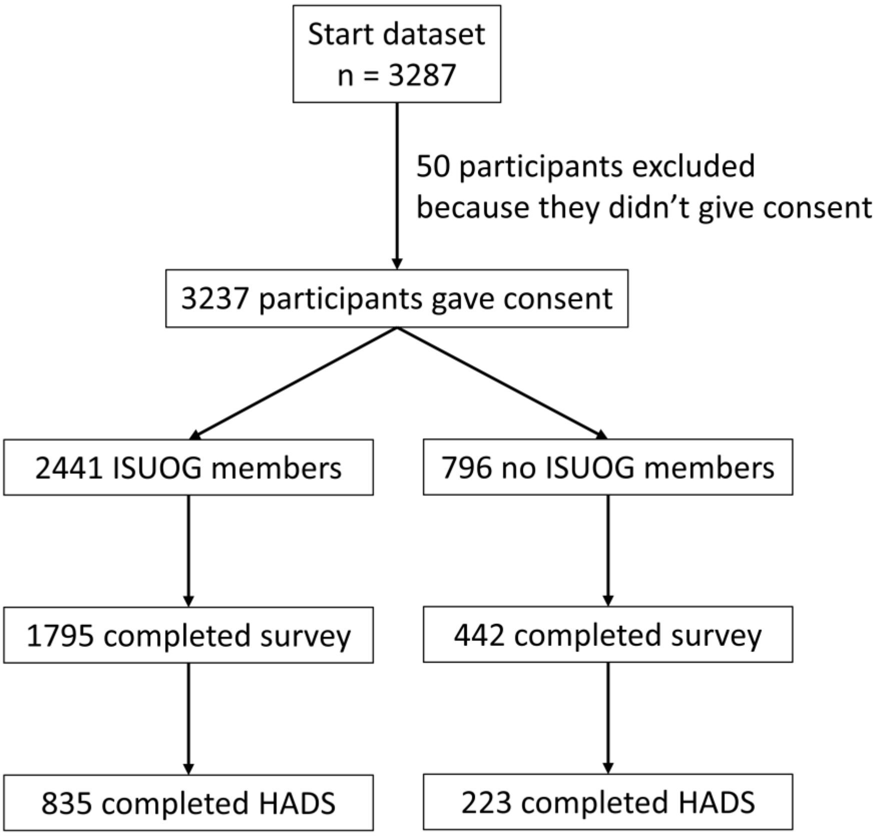

Response rate:
ISUOG members - 23.1% (2441/10589)
Non-ISUOG members - 3.2% (796/24920).

view only

Supplement 6: Demographics of survey participation, differentiated based on ISUOG membership (n = 3237).

	ISUOG members (n = 2441)	Others (n = 796)
**Total ISUOG mailing list (n)**	10589	24920
**Response of total ISUOG mailing list (n, %)**	2441 (23%)	796 (3%)
Countries (n)	120	78
Number of respondents in English (n)	1603	357
Number of respondents in Spanish (n)	564	317
Number of respondents in Italian (n)	195	72
Number of respondents in French (n)	79	50
**Completed survey**	**1795**	**442**
**Countries (n)**	110	65
**Age (mean, range)**	47.2 (18-82)	47.7 (25-78)
≤20 years	1 (0%)	0 (0%)
21-30 years	95 (5%)	22 (5%)
31-40 years	454 (25%)	104 (24%)
41-50 years	549 (31%)	137 (31%)
51-60 years	475 (26%)	121 (28%)
61-70 years	201 (11%)	50 (11%)
>70 years	18 (1%)	6 (1%)
**Gender (n, %)**		
Female	1176 (66%)	298 (68%)
Male	613 (34%)	142 (32%)
Intersex	1 (0%)	0 (0%)
Prefer not to say	3 (0%)	0 (0%)
**Prevalence suspected or confirmed SARS-CoV-2 based on symptoms +/- PCR testing (%)**	230 (13%)	60 (14%)
**Is PPE used by respondents (n, %)**		
Not yet	217 (12%)	62 (14%)
Yes - for aerosol generating procedures only	110 (6%)	23 (5%)
Yes - for all patients	1142 (64%)	250 (57%)
Yes - for suspected/positive patients only	324 (18%)	105 (24%)
**What PPE is used by respondents (n, %)**		
None	59 (3%)	17 (4%)
Gloves only	38 (2%)	11 (3%)
Gloves + surgical mask	393 (22%)	104 (24%)
Gloves + surgical mask + visor	116 (6%)	36 (8%)
Gloves + FFP3 mask	83 (5%)	12 (3%)
Gloves + FFP3 mask + visor	39 (2%)	12 (3%)
Gloves + FFP3 mask + visor + gown + surgical hat	70 (4%)	20 (5%)
Patient wearing mask	1482 (83%)	359 (82%)

For peer review only

**Concerns regarding a lack of PPE (n, %)**		
No	475 (26%)	104 (24%)
Gloves	223 (12%)	55 (13%)
Gown	499 (28%)	130 (30%)
N95/FFP3 mask	1014 (57%)	265 (60%)
Surgical hat	187 (10%)	46 (10%)
Surgical mask	657 (37%)	163 (37%)
Visor	458 (26%)	124 (28%)
Surgical mask (patient)	479 (27%)	130 (30%)
**Type of PPE that has been re-used or recycled (n, %)**		
No	690 (38%)	175 (40%)
Gloves	27 (2%)	7 (2%)
Gown	229 (13%)	58 (13%)
N95/FFP3 mask	722 (40%)	164 (37%)
Surgical hat	74 (4%)	18 (4%)
Surgical mask	435 (24%)	130 (30%)
Visor	493 (27%)	128 (29%)
**Anxiety (mean, 95% CI)***	7 (6.7-7.3)	6.6 (6.1-7.1)
0-7 (none)	480 (57%)	140 (63%)
8-10 (mild)	180 (22%)	49 (22%)
11-21 (moderate to severe)	175 (21%)	34 (15%)
**Depression (mean, 95% CI)***	5.4 (5.2-5.7)	4.9 (4.4-5.4)
0-7 (none)	587 (70%)	173 (78%)
8-10 (mild)	171 (20%)	34 (15%)
11-21 (moderate to severe)	77 (9%)	16 (7%)

*Numbers of HADS respondents lower than for main survey (n = 835 ISUOG members, n = 223 Others)

For peer review only

1 **Supplement 7: Comparison of comorbidity in those with (n = 290) and without suspected**
2 **or confirmed SARS-CoV-2 (n = 1947) (Total n = 2237).**

	Suspected or confirmed SARS-CoV-2	Without suspected or confirmed SARS-CoV-2
Heart disease (n, %)	3 (1%)	43 (2%)
Lung disease (n %)	30 (10%)	160 (8%)
Use of antihypertensives (n, %)	39 (13%)	283 (15%)
Smokers (n, %)	13 (4%)	125 (6%)

3
4

For peer review only

Supplement 8: Comparison of HADS outcome score in those with (n = 162) and without suspected or confirmed SARS-CoV-2 (n = 896) (Total n = 1058).

	Suspected or confirmed SARS-CoV-2	Without suspected or confirmed SARS-CoV-2
Anxiety (mean, 95% CI)	7.4 (6.7-8.0)	6.8 (6.6-7.1)
0-7 (none)	86 (53%)	534 (60%)
8-10 (mild)	32 (20%)	197 (22%)
11-21 (moderate to severe)	44 (27%)	165 (18%)
Depression (mean, 95% CI)	5.8 (5.2-6.4)	5.2 (5.0-5.5)
0-7 (none)	105 (65%)	655 (73%)
8-10 (mild)	37 (23%)	168 (19%)
11-21 (moderate to severe)	20 (12%)	73 (8%)

For peer review only

Supplement 9: HADS breakdown by gender (n = 1058).

	Female	Male	Prefer not to say
**Number of respondents (n, %)**	754 (71%)	303 (29%)	1 (0%)
**Anxiety (mean, 95% CI)***	6.9 (6.6-7.2)	5.7 (5.3-6.2)	-
0-7 (None) (n, %)	450 (60%)	202 (67%)	1 (100%)
8-10 (Mild) (n, %)	148 (20%)	60 (20%)	0 (0%)
11-21 (Moderate to severe) (n, %)	156 (21%)	41 (14%)	0 (0%)
**Depression (mean, 95% CI)***	5.4 (5.2-5.7)	5.0 (4.6-5.4)	-
0-7 (none) (n, %)	529 (70%)	230 (76%)	1 (100%)
8-10 (mild) (n, %)	146 (19%)	59 (19%)	0 (0%)
11-21 (moderate to severe) (n, %)	79 (10%)	14 (5%)	0 (0%)

For peer review only

Supplement 10: HADS breakdown by risk of working area (n = 1058).

	Low risk	Moderate risk	High risk
**Number of respondents (n, %)**	23 (2%)	28 (3%)	1007 (95%)
**Anxiety (mean, 95% CI)***	8.3 (6.3-10.3)	5.1 (3.6-6.6)	6.6 (6.3-6.8)
0-7 (None) (n, %)	13 (57%)	21 (75%)	619 (61%)
8-10 (Mild) (n, %)	3 (13%)	3 (11%)	202 (20%)
11-21 (Moderate to severe) (n, %)	7 (30%)	4 (14%)	186 (18%)
**Depression (mean, 95% CI)***	8.2 (6.0-10.3)	4.3 (2.7-5.8)	5.3 (5.0-5.5)
0-7 (none) (n, %)	12 (52%)	19 (68%)	729 (72%)
8-10 (mild) (n, %)	4 (17%)	6 (21%)	195 (19%)
11-21 (moderate to severe) (n, %)	7 (30%)	3 (11%)	83 (8%)

For peer review only

Supplement 11: Comparison of HADS outcome score in those with (n = 788) and without PPE shortages (n = 270) (Total n = 1058).

	PPE shortages	No PPE shortages
Anxiety (mean, 95% CI)	7.4 (7.1-7.7)	5.5 (5.0-6.0)
0-7 (none)	430 (55%)	190 (70%)
8-10 (mild)	183 (23%)	46 (17%)
11-21 (moderate to severe)	175 (22%)	34 (13%)
Depression (mean, 95% CI)	5.7 (5.5-6.0)	4.1 (3.7-4.5)
0-7 (none)	540 (69%)	220 (81%)
8-10 (mild)	167 (21%)	38 (14%)
11-21 (moderate to severe)	81 (10%)	12 (4%)

For peer review only

STROBE 2007 (v4) Statement—Checklist of items that should be included in reports of *cross-sectional studies*

Section/Topic	Item #	Recommendation	Reported on page #
**Title and abstract**	1	(*a*) Indicate the study's design with a commonly used term in the title or the abstract	1-2
		(*b*) Provide in the abstract an informative and balanced summary of what was done and what was found	2-3
**Introduction**			
Background/rationale	2	Explain the scientific background and rationale for the investigation being reported	4
Objectives	3	State specific objectives, including any prespecified hypotheses	5
**Methods**			
Study design	4	Present key elements of study design early in the paper	6
Setting	5	Describe the setting, locations, and relevant dates, including periods of recruitment, exposure, follow-up, and data collection	6
Participants	6	(*a*) Give the eligibility criteria, and the sources and methods of selection of participants	6
Variables	7	Clearly define all outcomes, exposures, predictors, potential confounders, and effect modifiers. Give diagnostic criteria, if applicable	7
Data sources/ measurement	8*	For each variable of interest, give sources of data and details of methods of assessment (measurement). Describe comparability of assessment methods if there is more than one group	7
Bias	9	Describe any efforts to address potential sources of bias	*6-8*
Study size	10	Explain how the study size was arrived at	6
Quantitative variables	11	Explain how quantitative variables were handled in the analyses. If applicable, describe which groupings were chosen and why	7-8
Statistical methods	12	(*a*) Describe all statistical methods, including those used to control for confounding	7-8
		(*b*) Describe any methods used to examine subgroups and interactions	7-8
		(*c*) Explain how missing data were addressed	7-8
		(*d*) If applicable, describe analytical methods taking account of sampling strategy	7-8
		(*e*) Describe any sensitivity analyses	7-8
**Results**			

For peer review only

Participants	13*	(a) Report numbers of individuals at each stage of study—eg numbers potentially eligible, examined for eligibility, confirmed eligible, included in the study, completing follow-up, and analysed	9-11
		(b) Give reasons for non-participation at each stage	9-11
		(c) Consider use of a flow diagram	Supplementary information
Descriptive data	14*	(a) Give characteristics of study participants (eg demographic, clinical, social) and information on exposures and potential confounders	9-11, supplementary information
		(b) Indicate number of participants with missing data for each variable of interest	9-11
Outcome data	15*	Report numbers of outcome events or summary measures	9-19
Main results	16	(*a*) Give unadjusted estimates and, if applicable, confounder-adjusted estimates and their precision (eg, 95% confidence interval). Make clear which confounders were adjusted for and why they were included	9-19
		(*b*) Report category boundaries when continuous variables were categorized	9-19
		(*c*) If relevant, consider translating estimates of relative risk into absolute risk for a meaningful time period	9-19
Other analyses	17	Report other analyses done—eg analyses of subgroups and interactions, and sensitivity analyses	9-19
**Discussion**			
Key results	18	Summarise key results with reference to study objectives	20
Limitations	19	Discuss limitations of the study, taking into account sources of potential bias or imprecision. Discuss both direction and magnitude of any potential bias	20-24
Interpretation	20	Give a cautious overall interpretation of results considering objectives, limitations, multiplicity of analyses, results from similar studies, and other relevant evidence	20-24
Generalisability	21	Discuss the generalisability (external validity) of the study results	20-24
**Other information**			
Funding	22	Give the source of funding and the role of the funders for the present study and, if applicable, for the original study on which the present article is based	25

*Give information separately for cases and controls in case-control studies and, if applicable, for exposed and unexposed groups in cohort and cross-sectional studies.

Note: An Explanation and Elaboration article discusses each checklist item and gives methodological background and published examples of transparent reporting. The STROBE checklist is best used in conjunction with this article (freely available on the Web sites of PLoS Medicine at http://www.plosmedicine.org/, Annals of Internal Medicine at http://www.annals.org/, and Epidemiology at http://www.epidem.com/). Information on the STROBE Initiative is available at www.strobe-statement.org.

For peer review only
